# Distinct taxonomic and functional profiles of high Arctic and alpine permafrost-affected soil microbiomes

**DOI:** 10.1186/s40793-023-00509-6

**Published:** 2023-06-16

**Authors:** Ciro Sannino, Weihong Qi, Joel Rüthi, Beat Stierli, Beat Frey

**Affiliations:** 1 Department of Agricultural, Food and Environmental Sciences, University of Perugia, Perugia, Italy; 2 Functional Genomics Center Zurich, ETH Zurich and University of Zurich, Zurich, Switzerland; 3 Swiss Institute of Bioinformatics SIB, Geneva, Switzerland; 4 Rhizosphere Processes Group, Swiss Federal Institute for Forest, Snow and Landscape Research (WSL), Birmensdorf, Switzerland

**Keywords:** High Arctic, European alps, Metagenome, Functionality, Permafrost, Active layer

## Abstract

**Background:**

Global warming is affecting all cold environments, including the European Alps and Arctic regions. Here, permafrost may be considered a unique ecosystem harboring a distinct microbiome. The frequent freeze–thaw cycles occurring in permafrost-affected soils, and mainly in the seasonally active top layers, modify microbial communities and consequently ecosystem processes. Although taxonomic responses of the microbiomes in permafrost-affected soils have been widely documented, studies about how the microbial genetic potential, especially pathways involved in C and N cycling, changes between active-layer soils and permafrost soils are rare. Here, we used shotgun metagenomics to analyze the microbial and functional diversity and the metabolic potential of permafrost-affected soil collected from an alpine site (Val Lavirun, Engadin area, Switzerland) and a High Arctic site (Station Nord, Villum Research Station, Greenland). The main goal was to discover the key genes abundant in the active-layer and permafrost soils, with the purpose to highlight the potential role of the functional genes found.

**Results:**

We observed differences between the alpine and High Arctic sites in alpha- and beta-diversity, and in EggNOG, CAZy, and NCyc datasets. In the High Arctic site, the metagenome in permafrost soil had an overrepresentation (relative to that in active-layer soil) of genes involved in lipid transport by fatty acid desaturate and ABC transporters, i.e. genes that are useful in preventing microorganisms from freezing by increasing membrane fluidity, and genes involved in cell defense mechanisms. The majority of CAZy and NCyc genes were overrepresented in permafrost soils relative to active-layer soils in both localities, with genes involved in the degradation of carbon substrates and in the degradation of N compounds indicating high microbial activity in permafrost in response to climate warming.

**Conclusions:**

Our study on the functional characteristics of permafrost microbiomes underlines the remarkably high functional gene diversity of the High Arctic and temperate mountain permafrost, including a broad range of C- and N-cycling genes, and multiple survival and energetic metabolisms. Their metabolic versatility in using organic materials from ancient soils undergoing microbial degradation determine organic matter decomposition and greenhouse gas emissions upon permafrost thawing. Attention to their functional genes is therefore essential to predict potential soil-climate feedbacks to the future warmer climate.

**Supplementary Information:**

The online version contains supplementary material available at 10.1186/s40793-023-00509-6.

## Introduction

Climate change is a growing environmental crisis with the capacity to affect the phenology, physiology, and community structures of most life forms on Earth [1, 2]. Specifically, global warming resulting from greenhouse gas emissions (e.g. CO_2_, CH_4_, and N_2_O) is affecting the functioning of ecosystems [3, 4]. The Arctic and European Alps seem to be more affected by global warming than other areas of Earth, with predictions of mean annual air temperature increases ≥ 8℃ above pre-industrial levels (before 1750) by 2100 [3, 5]. In the European Alps about 0.25 °C warming per decade until the mid of the 21st century and accelerated 0.36 °C warming per decade in the second half of the century is expected [6, 7], while in the Arctic, the rate of warming is four times the global average [8].

In regions where the mean annual temperature is under the freezing point, temperature fluctuations due to global warming can determine freeze–thaw cycles, affecting active-layer and permafrost habitats [9]. Permafrost soil is considered a closed system because it is not heterogeneous regarding the distinct geomorphological features that create challenges for microbial life [10, 11]. Indeed, while our understanding of the survival mechanisms developed by microorganisms is still limited, both prokaryotic and eukaryotic microbial communities have been demonstrated to be capable of metabolic activity at sub-zero temperatures [12–15]. Permafrost soils are diverse in terms of carbon and ice content and physico-chemical characteristics [16, 17]. Therefore, permafrost may be considered a unique ecosystem harboring a distinct microbiome [18].

Permafrost microbial diversity can be high compared with that in the overlaying active layer. The processes occurring below freezing point may be inferred from genetic changes in the microbial community and in the distribution of functional traits [19–21]. In the active layer, on the other hand, the microbial community can be altered by frequent freeze–thaw cycles, which modify soil ecosystem processes [22]. Microbial cells of the active layer undergo chemical and physical stresses (e.g. fluctuations in soil water and nutrient availability, increases in osmotic pressure), resulting in continuous changes in the dominant microbial community and consequently in its functions [21, 23, 24]. Both permafrost thawing and frequent freeze–thaw cycles lead to the release of labile carbon (C) and nutrients from the soil and a decrease in the rates of soil respiration and nitrogen (N) mineralization. Consequently, microbial metabolic activities can occur even at low temperatures, from the degradation of complex organic matter to the utilization of simpler C sources [22, 25–27]. A more thorough understanding of the functional potential of the permafrost and active-layer microbiomes may be a crucial step to predict their responses to global warming.

Studies on the Arctic microbiome have indicated that Arctic permafrost contains more genes related to anaerobic respiration and fermentation relative to the overlying active layer [28–30]. Moreover, Arctic permafrost is characterized by a high abundance of genes involved in the decomposition of organic polymers [31–34] and genes involved in stress responses, DNA repair, cell defense, and competition [34–38]. In European Alps, studies on the microbiome in permafrost areas are very limited and lack functional information [18, 21, 22, 39–41]. However, a recent metagenomic analysis of 12,000-year-old permafrost and active layers demonstrated that microorganisms existing in both permafrost and active layer of the Swiss Alps can thrive in the cold under oligotrophic conditions, highlighting their metabolic versatility in C and N cycling [42].

Chemical, physical, and lithological parameters differ between alpine and Arctic permafrost soils. Alpine permafrost soils have a deeper active layer (> 1.5 m), higher mean annual soil temperatures (> -2 °C), and lower organic C and water content [21, 43–46]. Because of these differences, alpine and Arctic permafrost soils may diverge in soil microbial diversity, function, and community structure.

In this study, the microbial functional diversity and metabolic potential of alpine and High Arctic permafrost and active-layer soils were analyzed using shotgun metagenomics. Permafrost and active-layer soils were collected from two extreme environments: Val Lavirun (LAV) is located on a rock glacier in the Swiss Alps and is characterized by very low pH (< 4.5) and pyrite formation [47]; the Villum Research Station (VRS) is located in the High Arctic in North Greenland at 81 °N and is characterized by very low temperatures and the presence of snow cover almost all year [48]. All these conditions can affect the microbial communities harbored in these extreme environments. Therefore, the main aims of the present work were to determine: (i) whether a distinct microbial and functional gene diversity and structure exists between the two locations (High Arctic and alpine soils) and between permafrost and active-layer soils; (ii) whether permafrost microbial communities which remain frozen throughout the year exhibit a higher proportion of cold-stress genes and genes related to cell defence and competition than surface communities that experiences seasonal thaw and refreezing; and (iii) whether distinct C- and N-cycling genes exist in permafrost soils than in active-layer soils.

## Materials and methods

### Study sites, soil characteristics, and soil collection

**Fig. 1:**
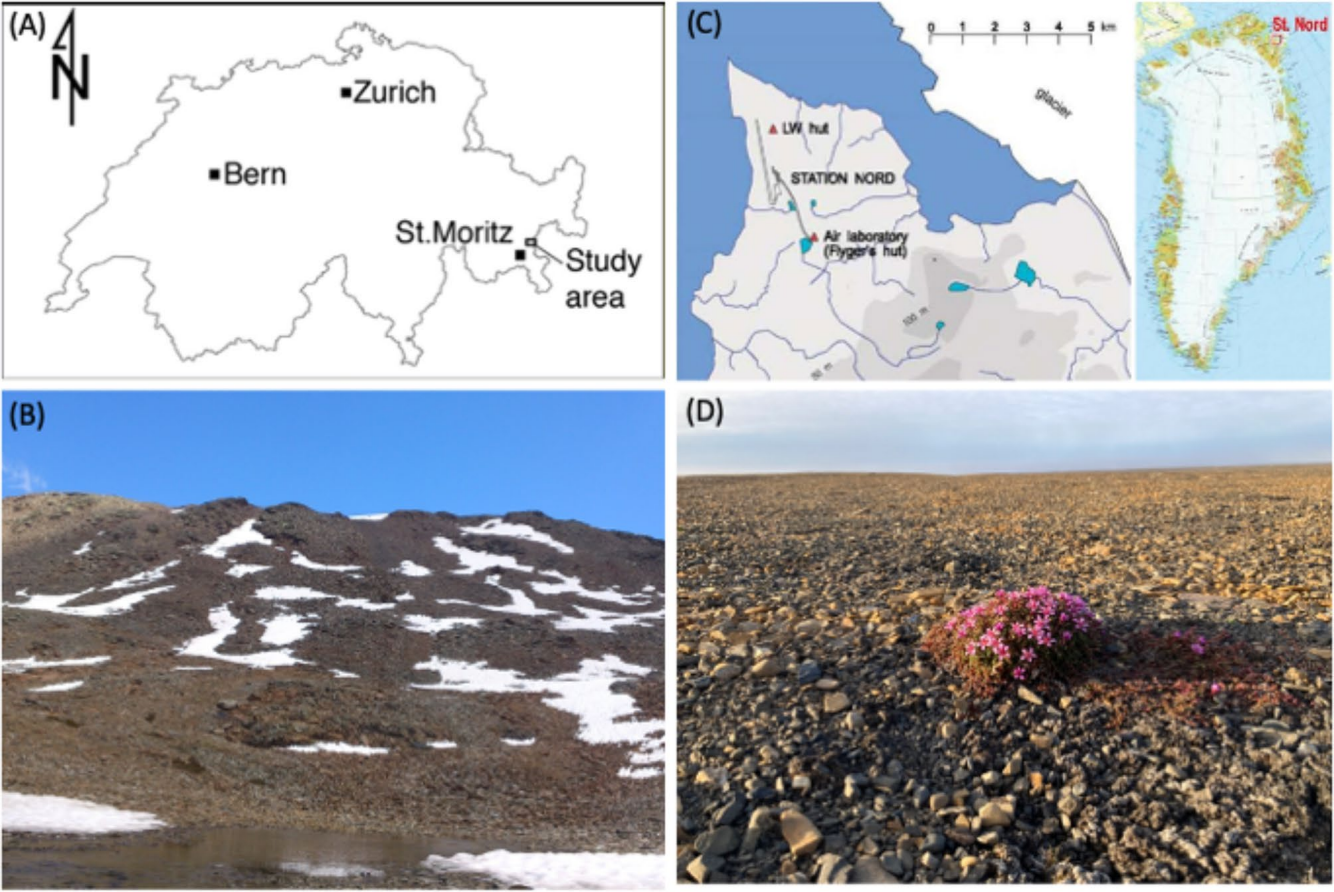
Sampling sites (**A**) Val Lavirun (LAV), eastern Swiss Alps. (**B**) Photograph of the permafrost regions below the Piz Lavirun (**C**) Villum Research Station (VRS), northern Greenland. (**D**) Photograph of the arctic tundra

Soils were collected from two geographical regions. The alpine site (46º31’1.9” N, 10º02’57.7” E, 2730 m a.s.l.) encompasses a remote area in Val Lavirun (LAV) at the origin of the mountain stream Ova Lavirun, in the Engadin area of Switzerland close to the Swiss–Italian boarder (Fig. 1; [47]. The mean annual temperature (MAT) in the region was − 1.8 °C and the mean annual precipitation (MAP) was 941 mm for the period 2016–2019 (Bernina automatic meteorological station, 46º44’10.9” N, 09º98’68.8” E, 2090 m a.s.l.; www.meteoswiss.admin.ch). Geologically, the Val Lavirun is part of the Languard nappe, which belongs to the crystalline basement of the upper Austroalpine unit of the Alps, which is mainly exposed in eastern Switzerland and Austria [49]. Two main rock types are exposed in the study area: (i) a chlorite mica schist of sedimentary origin, and (ii) a coarse-grained, leucocratic metagranite [47]. The soils in the study site are mostly composed of gravel and sand, with low pH (< 4.5) and extremely low concentration of C (< 1%) and N (≤ 0.1%). The low pH observed in the stream of Ova Lavirun is due to the oxidation of pyrite occurring naturally in the finer-grained portion of the chlorite mica-schist bedrock [47]. Due to the high altitude, the study area is typically covered by snow from November to July. The northwestern flank of the mountain features a rock glacier and continuous permafrost below 80 cm soil depth. Vegetation is scarce, basically representing barren soil with some rare individual occurrences of plants.

The High Arctic site is located in northern Greenland at the Villum Research Station (VRS, 81° 36’ N, 16° 40’ W, 24 m a.s.l.; Fig. 1). Minimum and maximum soil temperatures at 5 cm soil depth were − 16.3 and 19.0 °C for the period 2018–2019. Mean soil temperature was 5.8 ± 3.8 °C in August 2018 and 10.2 ± 3.7 °C in July 2019 [48]. Soils were covered by snow from the beginning of October 2018 until the beginning of July 2019 [48]. MAP in the region is 188 mm for the period 2016–2019 (https://eu-interact.org/field-sites/villum-research-station/). Permafrost is continuous in the area and is estimated to occur below a soil depth of 20 cm (https://eu-interact.org/field-sites/villum-research-station/). The bedrock is described as quaternary, undifferentiated cover and is dominated by carbonate minerals (https://data.geus.dk). The terrain features patterned ground forms (i.e. ice-wedge polygons) with patchy occurrences of biological soil crusts and scattered vascular plants, in particular *Saxifraga oppositifolia* (family Saxifragaceae), *Papaver radicatum* (family Papaveraceae), and *Draba* spp. (family Brassicaceae).

Soils in LAV were collected on 4 July 2018. Three soil profiles were excavated with shovels down to a depth of 100 cm. The distance between the three soil profiles was approximately 20 m. Each profile included an A horizon (0–5 cm depth; mineral A horizon enriched with organic matter and thus darker than the underlying B horizon), B horizon (5–45 cm depth), Bc horizon (> 45 cm soil depth; transitional layer with characteristics of both B and C horizons, with B horizon characteristics dominant; Bc seems to be partly affected by cryoturbation, as manifested by a disrupted horizon), and permafrost > 80 cm soil depth. Soil temperature measured along the soil profiles during the excavation were < 0 °C at a depth of 80 cm (data available on request). For this study, A, B and Bc (until 80 cm depth) were considered active soil layers. Biological soils crusts were absent in LAV. Five subsamples of bulk soil (≥ 10 g each) were collected of each profile at four different depths (A = 5 cm, B = 25 cm, Bc = 45 cm, pF = 90 cm). The collected subsamples per depth and profile were pooled and homogenized in autoclaved bags, and roots were removed when present. Soils were transported in dry ice to the WSL laboratory facilities, where they were sieved with a 4 mm mesh and stored at -20 °C. A total of 12 soil samples were further analyzed from LAV (3 profiles × 4 layers).

Soils in VRS were collected on 21 July 2018 during a field campaign. Three soil profiles located 150 m apart from each other were excavated with shovels down to a depth of 60 cm. In line with the existing information about permafrost depth at VRS (https://eu-interact.org/field-sites/villum-research-station/), temperature measured along the soil profiles during the excavation were < 0 °C at a depth of 30 cm (data available on request). The profiles comprised soil layers with the same characteristics as in LAV, with the addition of a biological soil crust. Five subsamples of bulk soil (≥ 10 g each) were collected from each profile at five different depths (biological soil crust = 0–1 cm, A = 5 cm depth, B = 15 cm depth, Bc = 25 cm depth, permafrost > 45 cm soil depth). The subsamples per depth and profile were pooled and homogenized in autoclaved bags, and roots were removed when present. Soil subsamples were sieved with a 4 mm mesh and frozen at -20 °C at the VRS facility. Soils were transported frozen to the WSL laboratory facilities, where they were stored at -20 °C before analyses. In total, 15 samples were analyzed from VRS (3 profiles × 5 layers).

Pre-sterilized equipment was used during the collection and processing of the soil samples in the field of both sites. The external layer of the profiles, exposed to air, was removed with spatulas freshly sterilized in 70% ethanol solution prior to bulk soil sampling, to eliminate debris and to prevent cross-contamination from upper soil layers.

### Soil properties

Soil physico-chemical parameters were analyzed as previously described [50, 51]. The gravimetric water content of soils was determined by weighing sample before and after drying. Soil pH was determined in water. The soil texture was analyzed according to [52] after acid digestion with H_2_O_2_. The percentage of total organic carbon (TOC) of dried, homogenized soils was measured in duplicate using a TOC analyzer (Shimadzu, Tokyo, Japan) after HCl (10%) acid digestion to remove carbonates. Total carbon (C) and nitrogen (N) were measured for dried (65 °C) and fine-grained soil samples, using an elemental analyzer (NC-2500; CE Instruments, Wigan, UK). Dissolved organic C (DOC) and dissolved N (DN) were measured in carbonate-free soil extracts treated with 3 M HCl, using a TOC/DTN analyzer (Sakalar Analytical B.V., Breda, the Netherlands). The soil extracts were prepared in milliQ water (water:soil 10:1 v/w, shaken overnight at room temperature) and filtered through DF 5895 − 150 ashless paper (Albert LabScience, Dassel, Germany). Organic matter (OM) content was determined by the weight-loss-on-ignition method [53]. Radiocarbon dating of TOC was performed using the Accelerator Mass Spectrometry facility at the Swiss Federal Institute of Technology (AMS ETH Zurich, Switzerland) as previously described [18]. Fine-ground samples (7–8 mg) were treated with acid (HCl) to remove carbonates. An equivalent of 0.5–1.0 mg of C was placed in tin cups for combustion in an elemental analyzer and subsequent graphitization. ^14^C concentrations were measured relative to the absolute atmospheric radiocarbon content of the atmosphere in 1950 AD after background correction and δ^13^C normalization. ^14^C ages (in BP, where 0 BP = AD 1950) were calculated and converted to calendar years (cal. yr BP) using the INTCAL13 calibration curve [54].

### DNA extraction, amplicon sequencing, and bioinformatic processing

Total DNA was extracted from 20 g of stored soil using the DNeasy PowerMax Soil Kit (Qiagen, Hilden, Germany), and the DNA extracts were quantified with PicoGreen (Invitrogen, Carlsbad, CA, USA), following the manufacturer’s instructions. To remove foreign DNA and prevent microbial contamination, workbench surfaces and non-autoclavable materials were cleaned with 5% sodium hypochlorite and 70% ethanol solutions prior to the DNA extractions. Triplicate PCR amplifications of the V3–V4 region of the 16S rRNA gene (prokaryotes) and of the ITS2 genomic region (fungi) were performed on 10 ng of the extracted DNA samples, using the primer pairs 341F/806R and ITS3/ITS4 under the conditions described in [18]. Negative controls for the DNA extractions (extraction buffer without soil) and PCR amplifications (high-purity water without DNA template) were included. The amplicon triplicates were pooled, purified (AMPure XP beads, Beckman Coulter, Beverly, MA, USA), and sent to the Génome Québec Innovation Centre at McGill University (Montreal, Canada), where the pooled amplicons were paired-end sequenced using the Illumina MiSeq v3 platform (Illumina Inc., San Diego, CA, USA). Raw sequences were deposited in the NCBI Sequence Read Archive under the BioProject accession identifier PRJNA917658.

Bioinformatics analyses were carried out using the Quantitative Insights Into Microbial Ecology 2 program (QIIME 2, ver. 2020.6.0, [55]) run on the WSL Hyperion cluster. The raw paired-end FASTQ files were imported into the QIIME2 program and demultiplexed using a native plugin. Thereafter, the Cutadapt plugin was processed to trim sequencing primers. The Divisive Amplicon Denoising Algorithm 2 (DADA2) plugin in QIIME2 was used for quality filtering and determine amplicon sequence variants (ASVs; [56]. Demultiplexed sequences from each sample were quality-filtered, de-noised and merged, and chimeric sequences were identified and removed [57]. We applied the parameter with truncation length of 270 bp for forward and 215 bp for reverse (for bacteria) and of 240 bp for forward and 190 bp for reverse (for fungi). Taxonomic assignment was accomplished using the Naive Bayes q2-feature-classifier [58] in QIIME2 against the SILVA ribosomal RNA gene database v138 [59] and UNITE database v8.2 [60]. Prokaryotic ASVs mapped to mitochondria and chloroplasts were filtered out of the resulting prokaryotic feature table. The fastq files were randomly subsampled to the smallest read number, resulting in 26,358 bacterial and 18,109 fungal reads per sample. The subsampling enabled a more accurate comparison of the richness of the different samples.

### Shotgun sequencing and bioinformatic processing

Library preparation using the NEB Next ultra-DNA Prep Kit (Illumina Inc., San Diego, California, USA) and shotgun sequencing of the eluted DNA samples were performed at the Genome Quebec Innovation Centre at McGill University (Montreal, Canada), using the HiSeq 2500 system (2 × 125 bp; Illumina Inc.), as previously outlined [42, 61]. For shotgun sequencing we compared samples from the A horizon (0–5 cm depth) with permafrost samples of both sites. There were two permafrost samples, one from LAV and one from VRS, with an insufficient amount of DNA (< 100 ng) for the library preparation. Consequently, a total of ten metagenomes were sequenced from the two sites, with three replicates of A horizon and two replicates of permafrost soils per site. Raw sequences were deposited in the NCBI Sequence Read Archive under the BioProject accession identifier PRJNA917667.

Pre-processing of metagenomic reads, assembly of reads into contigs, contig binning, and functional and phylogenetic annotation of contigs and bins were achieved using a customized pipeline [42, 51, 62]. Briefly, raw reads were quality checked using FastQC v0.11.8 (https://www.bioinformatics.babraham.ac.uk/projects/fastqc/). They were quality filtered and trimmed (i.e. pre-processed reads) using Trimmomatic v0.36 (Q = 20, minimum read length = 40; [63]. Pre-processed read pairs and singletons were assembled into contigs (> 200 bp) by iteratively building de Bruijn graphs using k-mers of increasing size with the de novo assembler MEGAHIT v1.2.9 (–k-min 27 –k-step 10; [64].

Protein-coding sequences contained in the assembled contigs were predicted with MetaGeneMark v3.38 [65]. To determine the potential metabolic capabilities of the soil metagenomes, protein-coding genes were assigned to functions (i.e. functional genes). About 50% of the predicted genes were assigned to general metabolic and cellular functions through EggNOG v4.5 database, which classifies the genes to clusters of orthologous groups (COGs) of proteins and organizes the COGs into general functional categories [66]. Annotation to EggNOG v4.5 was performed using eggnog-mapper v1.0.3 with the DIAMOND search mode against all protein sequences [67]. About 1% of the protein-coding genes were assigned to CAZys using the CAZy database (July 2017 release; [68]. About 0.2% of the genes were assigned to N-cycling families using the NCyc database [69]. Annotations against the CAZy and NCyc databases were performed using SWORD v1.0.4 [70] with the same parameters (-v 10^− 5^) as in [71]. In addition to the categorization by enzyme classes implemented in CAZy, a manual categorization of CAZy genes into different C substrates was performed as previously outlined [72, 73].

Pre-processed read pairs from each of the samples were mapped to the assembled contigs, using BWA aligner v0.7.15 (bwa-mem; [74]. Mapping of the reads to the assembled protein-coding gene sequences to obtain gene abundances was done using the function featureCounts from the package Subread v2.0.1 (-minOverlap 10, Q = 10, -primary; [75]. Counts of predicted genes were normalized to reads per kilobase (RPK).

Predicted protein-coding genes annotated to the functional databases (e.g., eggNOG) were assigned taxonomically using Kaiju 1.7.4 [76] with the precompiled NCBI BLAST nr + euk database (version 2021–02–24) and default settings. The helper program kaiju-addTaxonNames was utilized to convert NCBI taxon IDs to taxonomy. Additionally, the -ssu_finder function in CheckM v1.1.3 [77] was applied to identify SSU rRNA sequences in the contigs within the bins (16 S for prokaryotes and 18 S for eukaryotes). These sequences were taxonomically annotated via the SINA online aligner v1.2.12 [78] against the SILVA database (release 138; [59]. To estimate the abundance of the 16 and 18 S rDNA genes the corresponding read counts per contig were normalized to the contig length in kbp.

### Bacterial and fungal abundance

Bacterial and fungal biomass were estimated by quantitative PCR (qPCR) on a 7500 Fast Real-Time PCR System (Thermo Fisher Scientific, Waltham, MA, USA). qPCR reactions were prepared using 6.6 µl of the DNA extracts and the primer pairs 27F/519R and ITS3/ITS4, which amplify the V1–V3 region of the 16 S rRNA gene in bacteria and the ITS2 genomic region in fungi, respectively. qPCR programs were performed as described by [50, 51].

### Statistical analyses

All statistical analyses were conducted using the open-source software R version 4.0.3 [79] and graphical representations of results were created with the R package ggplot2. Differences among chemical and physical parameters, richness, Shannon-H index, and quantitative PCR data were tested using two-way factorial analysis of variance (ANOVA) followed by Tukey’s honestly significant difference (HSD) post-hoc tests. Non-metric multidimensional scaling (NMDS) ordination was performed for both bacterial and fungal communities, and the envfit function (vegan R package 4.03) was used to highlight the correlation between chemical and physical parameters and the soil microbial communities. Principal coordinate analysis (PCoA) based on Bray-Curtis distances was implemented with PRIMER (v7, PRIMER-E, UK) to visualize the functional gene structure.

The differential abundance of the functional genes annotated with the EggNOG, CAZy, and NCyc databases was determined using the R package DESeq2 [62, 80]. Pairwise comparisons were made: (i) between the active layer (A horizon) and permafrost of LAV; (ii) between the active layer (A horizon) and permafrost of VRS; (iii) between the active layer (A horizon) of LAV and the active layer (A horizon) of VRS; and (iv) between the permafrost of LAV and the permafrost of VRS. For all pairwise comparisons, median-of-ratio normalization was applied to account for differences in sequencing depth among samples, and p values were adjusted for multiple testing using the Benjamini–Hochberg method with a false discovery rate threshold of 5%. A Welch’s test (or unequal variances t-test) was used to assess differences in protein-coding gene Shannon-H diversity calculated for the functional genes annotated with the EggNOG, CAZy, and NCyc databases.

## Results

### Soil properties and microbial properties change with soil depth

Soil physico-chemical properties differed strongly between the two sites and along the soil profiles (Table 1). The alpine site (Val Lavirun, LAV) was characterized by a lower pH (4.3–4.6) and C concentration (0.2–1.3%) compared with the High Arctic site (Villum Research Station, VRS; pH: 6.9–7.4; C: 0.7–2.1%). Wide variability in soil properties was found along the soil profiles. The most significant (p < 0.05) differences were found in the deepest layers of both localities: soil temperature, C and N concentration, and DOC decreased significantly with increasing soil depth. Inconsistent trends along the soil profiles were observed for gravimetric water content, which decreased significantly with increasing soil depth in LAV but showed the opposite pattern in VRS. Soil pH decreased significantly with increasing depth in LAV samples, while no significant differences were found among VRS soil layers. Finally, ^14^C age, a measure of C turnover, in the soils was between 2,000 and 12,000 years in LAV soil samples and between 3,000 and 25,000 years in VRS samples (Table 1). Bacterial and fungal alpha-diversity and qPCR analysis differed significantly (p < 0.05) among the soil layers in LAV and VRS (Table 2).

**Table 1:** Soil physico-chemical parameters of alpine (Val Lavirun, LAV) and High Arctic soils (Villum, VRS). Values represent means ± standard deviation (n = 3). Different superscript letters indicate significant (p < 0.05) differences between individual means, assessed by two-way factorial analysis of variance (ANOVA) followed by Tukey’s HSD post-hoc tests

Sample	Depth (cm)	Soil T (°C)	pH	C (%)	N (%)	DOC (ppm)	DN (ppm)	GWC (%)	C14
**LAV**									
aL_A	5	5.6 ± 0.5^a^	4.6 ± 0.1^a^	1.3 ± 0.1^a^	0.10 ± 0.00^a^	17.0 ± 2.2^a^	1.0 ± 0.1^a^	13.7 ± 1.3^ab^	2227 ± 424^a^
aL_B	25	1.9 ± 0.3^b^	4.6 ± 0.2^a^	1.0 ± 0.1^b^	0.08 ± 0.01^b^	15.4 ± 0.5^a^	0.9 ± 0.1^ab^	14.6 ± 1.5^a^	5379 ± 920^b^
aL_B_c_	45	0.3 ± 0.2^c^	4.5 ± 0.0^ab^	0.3 ± 0.1^c^	0.04 ± 0.00^c^	11.5 ± 1.5^b^	0.7 ± 0.1^ab^	11.6 ± 1.6^ab^	7902 ± 792^c^
pF	90	-0.3 ± 0.1^d^	4.3 ± 0.0^b^	0.2 ± 0.0^c^	0.03 ± 0.00^c^	8.1 ± 0.8^b^	0.6 ± 0.1^b^	10.1 ± 1.4^b^	12,529 ± 665^d^
**VRS**									
BSC	1	9.0 ± 1.1^a^	6.9 ± 0.4^a^	2.1 ± 0.6^a^	0.25 ± 0.04^a^	20.8 ± 5.8^a^	1.8 ± 0.3^a^	8.4 ± 0.4^a^	3412 ± 3296^a^
aL_A	5	6.6 ± 1.9^a^	7.0 ± 0.3^a^	1.5 ± 0.2^ab^	0.17 ± 0.01^b^	11.4 ± 0.6^b^	1.0 ± 0.3^b^	8.3 ± 0.7^a^	8401 ± 3043^ab^
aL_B	15	3.4 ± 0.8^b^	7.0 ± 0.3^a^	1.1 ± 0.1^bc^	0.13 ± 0.00^bc^	9.8 ± 1.1^b^	0.3 ± 0.0^c^	13.5 ± 0.8^b^	12,456 ± 5245^ab^
aL_B_c_	25	0.9 ± 0.5^bc^	7.2 ± 0.2^a^	0.7 ± 0.1^c^	0.11 ± 0.01^c^	10.1 ± 0.1^b^	0.3 ± 0.0^c^	13.9 ± 1.0^b^	16,209 ± 3848^bc^
pF	45	-0.1 ± 0.0^c^	7.4 ± 0.0^a^	1.2 ± 0.1^b^	0.11 ± 0.00^c^	8.2 ± 1.2^b^	0.5 ± 0.1^bc^	18.5 ± 1.5^c^	25,038 ± 4652^c^

C, carbon; N, nitrogen; DOC, dissolved organic carbon; DN, dissolved nitrogen; GWC, gravimetric water content; C14, carbon isotope ^14^C

BSC = biological soil crust; aL = active-layer soils; pF = permafrost soils

A = A horizon enriched with organic matter and thus darker than the underlying B horizon

B = B horizon

Bc = transitional layer with characteristics of both B and C horizons, with B horizon characteristics dominant; partly affected by cryoturbation, as manifested by transitions between horizons

**Table 2:** Microbial alpha-diversity and abundance of alpine (Val Lavirun, LAV) and High Arctic soils (Villum, VRS). Values represent means ± standard deviation (n = 3). Different superscript letters indicate significant (p < 0.05) differences between individual means, assessed by two-way factorial analysis of variance (ANOVA) followed by Tukey’s HSD post-hoc tests

Sample	Depth (cm)	Bacterial richness	Bacterial Shannon-H	Fungal richness	Fungal Shannon-H	16S copies (g^− 1^ dry soil)	ITS copies (g^− 1^ dry soil)	
**LAV**								
aL_A	5	657 ± 40^ac^	3.9 ± 0.2^ab^	47 ± 12^a^	2.9 ± 0.9^a^	(1.1 ± 0.7) × 10^8a^	(2.5 ± 1.5) × 10^6ab^	
aL_B	25	342 ± 25^b^	3.0 ± 0.2^b^	47 ± 30^a^	2.2 ± 0.4^a^	(5.2 ± 3.9) × 10^7a^	(4.6 ± 2.4) × 10^6a^	
aL_B_c_	45	849 ± 195^c^	4.1 ± 0.7^a^	235 ± 73^b^	3.2 ± 1.0^a^	(3.2 ± 2.1) × 10^7a^	(7.8 ± 0.9) × 10^5b^	
pF	90	437 ± 50^ab^	2.9 ± 0.1^b^	79 ± 25^a^	2.0 ± 1.1^a^	(3.6 ± 2.5) × 10^6a^	(2.8 ± 0.3) × 10^5b^	
**VRS**								
BSC	1	2265 ± 62^a^	6.4 ± 0.1^a^	205 ± 19^a^	3.23 ± 0.42^a^	(3.3 ± 0.9) × 10^7a^	(4.9 ± 3.1) × 10^6ab^	
aL_A	5	1888 ± 61^b^	6.0 ± 0.1^bc^	103 ± 17^b^	2.42 ± 0.118^b^	(2.2 ± 1.2) × 10^7a^	(4.6 ± 4.7) × 10^6a^	
aL_B	15	1460 ± 32^c^	5.8 ± 0.0^c^	58 ± 17^bc^	2.13 ± 0.13^b^	(3.1 ± 0.5) × 10^8a^	(6.6 ± 5.7) × 10^5b^	
aL_B_c_	25	1336 ± 70^c^	5.8 ± 0.1^c^	51 ± 10^c^	2.20 ± 0.23^b^	(1.4 ± 0.2) × 10^7a^	(8.2 ± 5.8) × 10^5b^	
pF_	45	1673 ± 42^d^	6.3 ± 0.2^ab^	98 ± 21^b^	2.66 ± 0.36^ab^	(1.1 ± 0.2) × 10^7a^	(2.0 ± 1.9) × 10^5ab^	

BSC = biological soil crust; aL = active layer soils; pF = permafrost soils

A = A horizon enriched with organic matter and thus darker than the underlying B horizon

B = B horizon

Bc = transitional layer with characteristics of both B and C horizons, with B horizon characteristics dominant; partly affected by cryoturbation, as manifested by transitions between horizons

16S copies = copy numbers of bacterial 16S gene; ITS copies = copy numbers of fungal ITS region

### Different taxonomic profiles between high Arctic and alpine soils

**Fig. 2:**
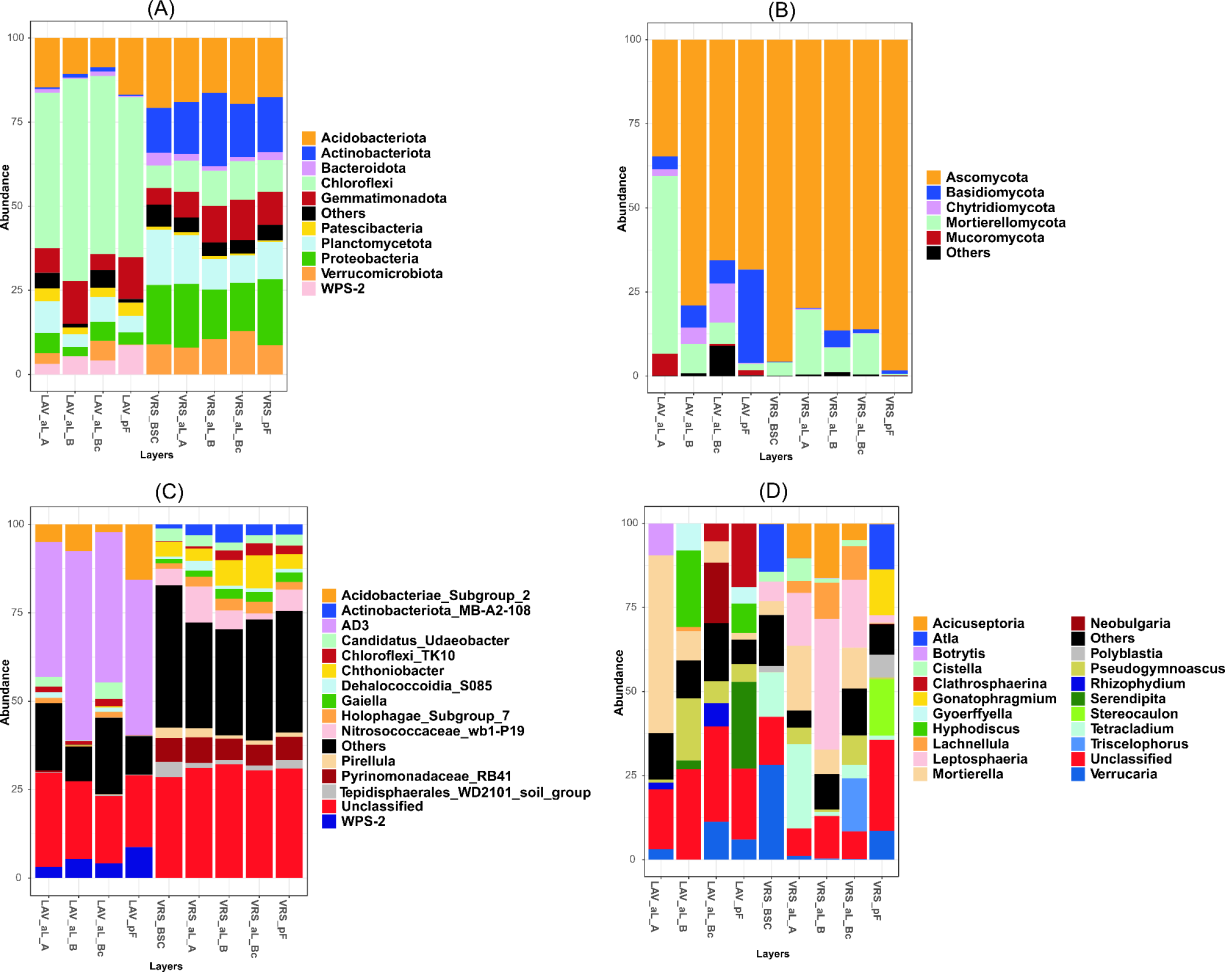
Soil bacterial and fungal diversity at the phylum and genus levels, according to amplicon sequencing (**A**) Bacterial phyla, (**B**) fungal phyla, (**C**) bacterial genera, (**D**) fungal genera. LAV, Val Lavirun; VRS, Villum Research Station BSC = biological soil crust; aL = active-layer soils. pF = permafrost soils. A = A horizon enriched with organic matter and thus darker than the underlying B horizon. B = B horizon. Bc = transitional layer with characteristics of both B and C horizons, with B horizon characteristics dominant; partly affected by cryoturbation, as manifested by a disrupted and broken horizon

Amplicon sequencing resulted in a total of 889,573 bacterial reads (414,967 in LAV and 474,606 in VRS) clustered into 7,238 bacterial ASVs. At the phylum level, the most abundant bacterial groups were Chloroflexi (with a relative abundance of 46–60%), Acidobacteriota (8–16%), and Gemmatimonadota (4–12%) in LAV soils, and Acidobacteriota (16–20%), Actinobacteriota (13–21%), Planctomycetota (8–16%), and Proteobacteria (14–19%) in VRS soils. Considering the ASVs classified at the genus level, a large number of unclassified bacteria were found in both sites (19–26% in LAV and 28–32% in VRS). Chloroflexi_AD3, with an abundance of 38–53%, was the most abundant bacterial genus found in LAV soils. In VRS soils the most abundant ASVs assigned at the family/genus level were *Chthoniobacter* (3–9%), Nitrosococcaceae wb1-P19 (1–10%), and Pyrinomonadaceae RB41 (5–7%; Fig. 2).

A total of 27,916 archaeal reads (19,844 in LAV and 8,072 in VRS) were clustered into 64 ASVs. Crenarchaeota was the most abundant phylum in both the alpine and the High Arctic site. In LAV, Crenarchaeota was the only phylum found in all soil layers. Only the uppermost (A) layer had a few ASVs ascribed to Thermoplasmatota (relative abundance of 5.4%). In VRS, the abundance of Crenarchaeota was > 75% in all soil layers (Additional File 1, Table S1).

A total of 634,279 fungal reads (266,506 in LAV and 367,773 in VRS) were clustered into 1,096 fungal ASVs. At the phylum level the most abundant fungal group in all soil samples was Ascomycota, with abundances of 34–98%, followed by Mortierellomycota (0.46–52%) and Basidiomycota (0.16–27%). The most abundant fungal genera were *Mortierella* (1.9–52%), *Hyphodiscus* (8.7–22%), *Serendipita* (0.09–25%), and *Pseudogymnoascus* (0.9–18.3%) in LAV soils, and *Leptosphaeria* (2.1–38%), *Tetracladium* (1.1–25%), and *Mortierella* (0.4–19%) in VRS soils (Fig. 2).

**Fig. 3:**
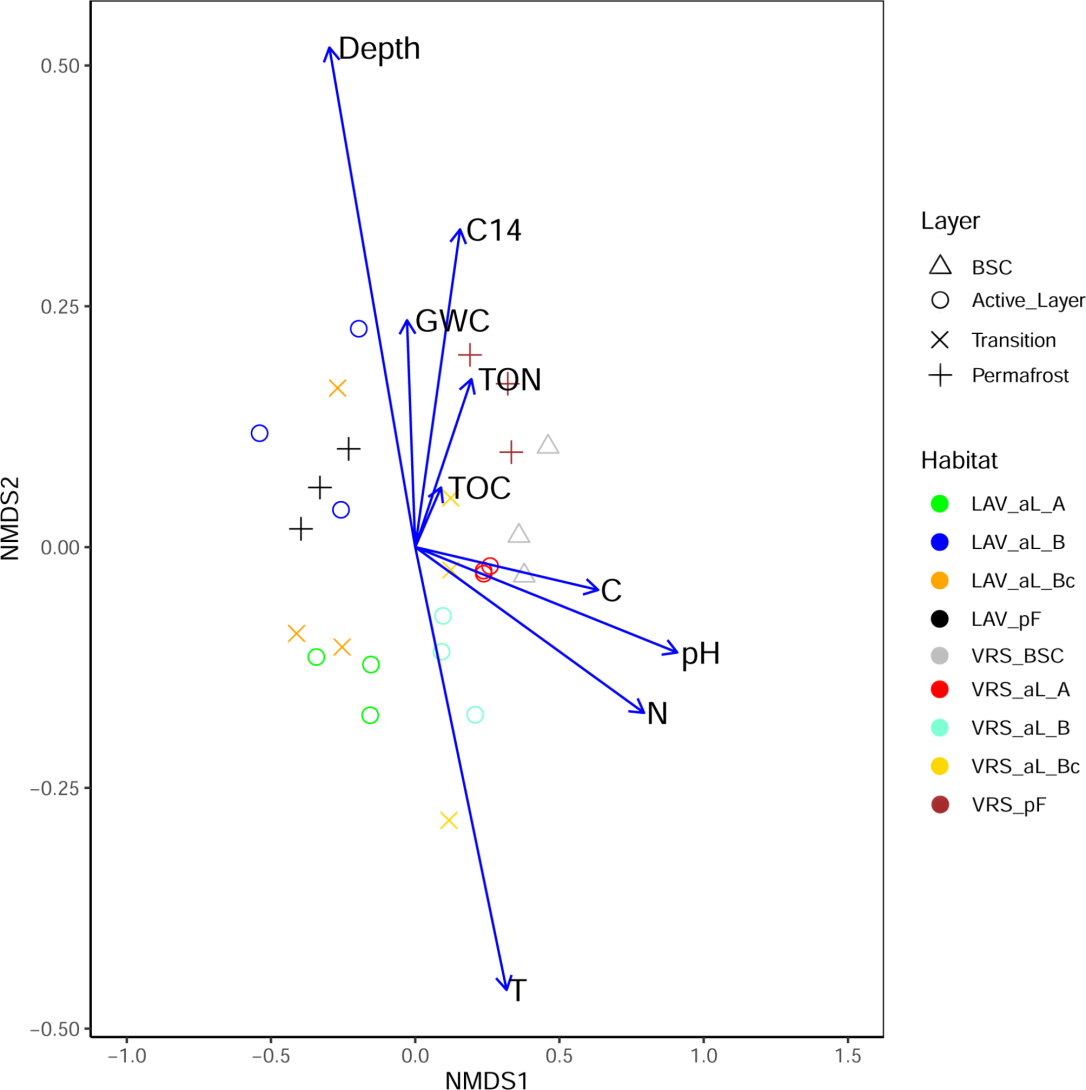
Non-metric multidimensional scaling (NMDS) plot of fungal communities of two sampling sites and soil profiles The envfit function was used to show the chemical and physical parameters affecting fungal communities. LAV, Val Lavirun; VRS, Villum Research Station Three replicates per soil depth are shown. BSC = biological soil crust; aL = active-layer soils. pF = permafrost soils. A = A horizon enriched with organic matter and thus darker than the underlying B horizon. B = B horizon. Bc = transitional layer with characteristics of both B and C horizons, with B horizon characteristics dominant; partly affected by cryoturbation, as manifested by a disrupted and broken horizon. GWC = gravimetric water content; TOC = total organic carbon; TON = total organic nitrogen; C14 = radiocarbon ^14^ C

Communities were distinct (p = 0.0001) between the two sites, while differences along the soil profile within a site were much smaller. Considering the beta-diversity analysis, the results of the NMDS-envfit ordination indicated a clear separation between LAV and VRS soils for both bacterial and fungal communities. However, while this separation could be visualized in an ordination plot for fungal communities (Fig. 3), it was not possible to visualize the bacterial communities between the two sites in NMDS or similar ordination because the individual sampling points differed too strongly between LAV and VRS. The envfit results indicated that the bacterial communities were significantly (p < 0.05) related to soil depth, temperature, pH, C, N, DOC, DN, and radiocarbon content. On the other hand, the fungal communities were significantly (p < 0.05) related only to soil depth, temperature, pH, and C and N content (Additional File 1, Table S2).

### Overall metagenome sequencing results

After quality filtering, we obtained 2,013,327,721 high-quality reads (42,071,440 to 71,249,495 reads per sample; Additional File 1, Table S3). The MEGAHIT assembly of reads into contigs produced a total of 2,549,245 contigs of 789 bp on average, ranging from 200 to 720,250 bp, with an N50 value of 897 and a GC content of 63%. In total, we found 3,952,470 predicted genes among the contigs, 2,000,785 of which we could annotate with the eggNOG database, 39,507 with the C-cycling gene database CAZy, and 6,373 with the N‐cycling gene database NCyc (Additional File 1, Table S3).

### Taxonomic composition of the metagenomes

Kaiju was used for the taxonomic classification of the contigs from the metagenomic analysis of 16 and 18 S rRNA genes, eggNOG, CAZy, and NCyc genes, and predicted genes. Overall, the taxonomic composition and the relative abundance of taxa was similar for the different databases used. Bacteria was always the most abundant group, with relative abundances > 90% in active layer and permafrost soils of both LAV and VRS (Additional File 2, Fig. S1). Generally, Archaea was more abundant in permafrost, while Eukaryota was more abundant in permafrost of LAV and in the active layer of VRS. In all annotations, viruses were always the least abundant group. In active-layer (A horizon) and permafrost soils in LAV, Chloroflexi was always the most abundant microbial group. On the other hand, Acidobacteria, Actinobacteria, and Proteobacteria dominated among the microbial groups in the A horizon and permafrost soils in VRS. A high abundance of unclassified predicted genes was found in both LAV and VRS soils. Considering fungi, high abundances of Ascomycota and Basidiomycota were only found in permafrost soil in LAV (18 S rRNA gene; Additional File 2, Fig. S1).

### Higher protein-coding gene diversity in active-layer than in permafrost soils

Overall, the soils collected from VRS, mainly active-layer soils, had a higher functional gene diversity (Shannon-H index) than LAV soils (Additional File 2, Fig. S2). Of the 20 eggNOG functional categories, RNA processing and modification (A) and chromatin structure and metabolism (B) were found only in LAV. Only the functional category (A) differed significantly in alpha-diversity (p < 0.05) between the active-layer and permafrost soils. In LAV, the functional categories cell cycle control, cell division, and chromosome partitioning (D), coenzyme transport and metabolism (H), translation, ribosomal structure, and biogenesis (J), and replication, recombination, and repair (L) had a higher alpha-diversity in permafrost soil. On the other hand, the functional categories lipid transport and metabolism (I), post-translational modification, protein turnover, and chaperones (O), inorganic ionic transport and metabolism (P), secondary metabolite biosynthesis, transport and catabolism (Q), signal transduction mechanisms (T), and intracellular trafficking, secretion, and vesicular transport (U) had a higher alpha-diversity in the active layer in LAV (Additional File 2, Fig. S2).

In VRS soils, a significantly higher Shannon-H index was found in the active layer for the functional categories transcription (K), cell motility (N), secondary metabolite biosynthesis, transport, and catabolism (Q), and intracellular trafficking, secretion, and vesicular transport (U). In the comparison of the same soil layer between the alpine and High Arctic sites, almost all functional categories differed significantly between the two localities. Only for the functional categories cell motility (N) and signal transduction (T) there were no significant differences (Additional File 2, Fig. S2).

For CAZy genes, a significantly higher alpha-diversity was found in the active layer than in permafrost. Significant differences were found for the auxiliary activities (AA; between soils from VRS; p = 0.047), carbohydrate-binding modules (CBM; between soils from VRS; p = 0.0304), and glycoside hydrolase families (GH; between soils from LAV; p = 0.0322). On the other hand, when considering differences in the same soil layer between the two localities, a significantly higher alpha-diversity was found in permafrost samples of VRS for CBM, GH, glycosyltransferase (GT), and polysaccharide lyase (PL) families (Additional File 2, Fig. S2).

The N-cycling genes annotated with the NCyc database were grouped into six main families. In LAV, significant differences were found between the soil layers only for nitrification (NT; p = 0.006654), with a higher Shannon-H index in active-layer compared with permafrost soils. In VRS, significant differences were found in assimilatory nitrate reduction (ANR; p = 0.0011) and denitrification (DNF; p = 0.024), with a higher Shannon-H index in the active layer than in permafrost. Regarding differences in the same soil layer between the two localities, as for CAZy families, significant (p < 0.05) differences in NCyc families were found only between permafrost samples. The NCyc families with this pattern were ANR, DNF, dissimilatory nitrate reduction (DNR), and organic degradation and synthesis (ODS) (Additional File 2, Fig. S2).

To investigate changes in the abundance of functional genes with location and soil layer, we calculated log_2_-fold changes (LFC) between LAV and VRS soils and between active-layer soils and permafrost soils in the different locations, separately for the genes annotated with the eggNOG, CAZy, and NCyc databases. Interestingly, clear separation between locations and between soil layers was detected by PCoA ordination. In particular, the microbial genetic potentials of the soil metagenomes from VRS were clearly different from the ones collected in LAV. Separation was also visible in LAV between active-layer and permafrost soils (Additional File 2, Fig. S3).

### Functional categories annotated with EggNOG

The comparison between the permafrost and active layer indicated 1755 differentially abundant genes (p < 0.01) in LAV and 2039 in VRS. In both sites, there were many overrepresented genes (higher positive LFC) in permafrost than in the A horizon (931 vs. 824 in LAV and 1632 vs. 407 in VRS; Additional File 1, Table S4). In the comparisons between permafrost in LAV and permafrost in VRS and between the active layer in LAV and the active layer in VRS, 4292 and 2099 differentially abundant genes (p < 0.01) were found, respectively. In the active layer (A horizon), COG genes were almost equally abundant between LAV and VRS (1006 vs. 1093), while in permafrost there were a large number of genes that were significantly (p < 0.01) overrepresented in LAV (2271 vs. 2021; Additional File 1, Table S4).

**Fig. 4:**
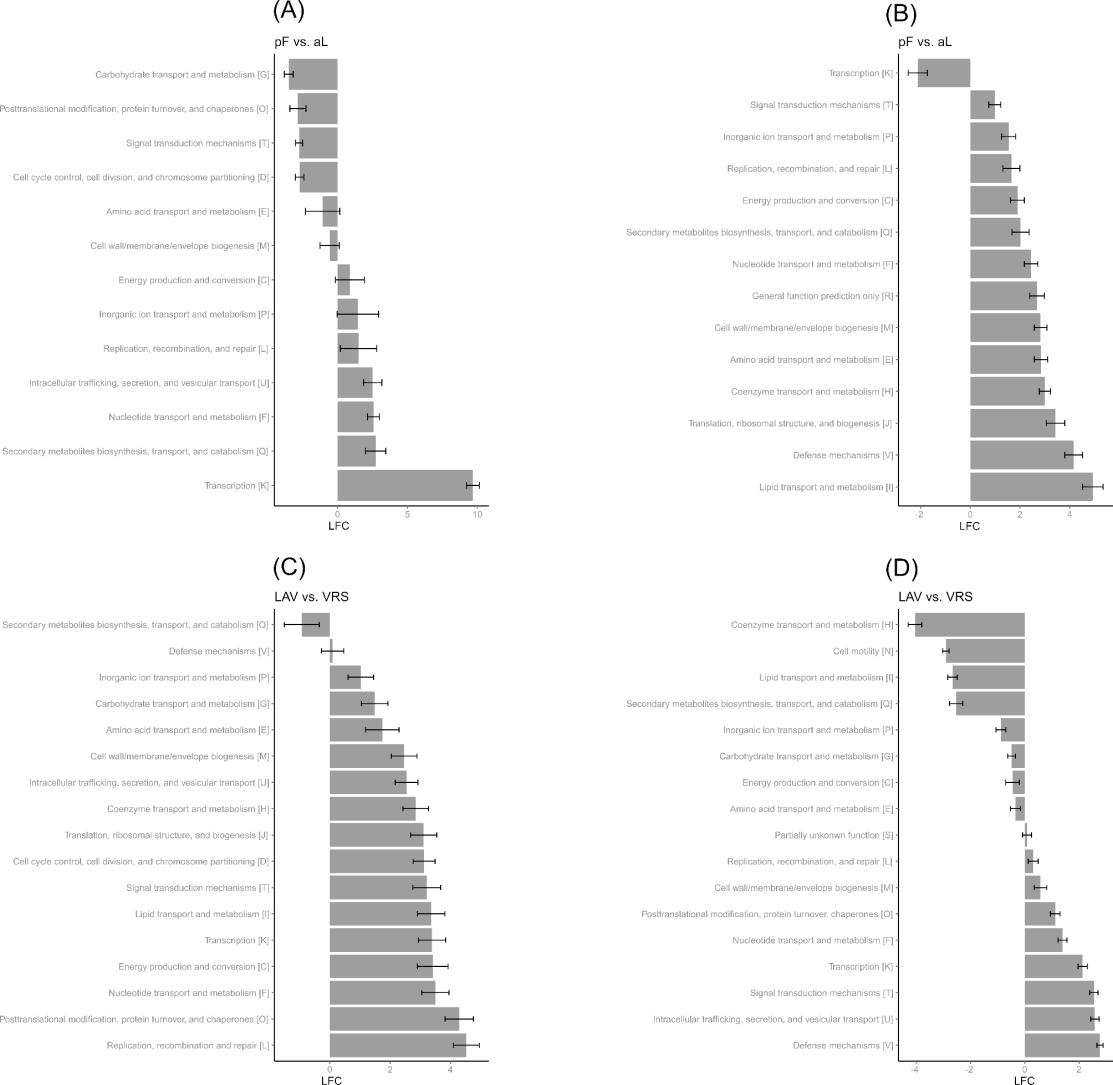
Differences in COG functional categories between the two sampling sites and soil profiles LAV, Val Lavirun; VRS, Villum Research Station; aL, active layer; pF, permafrost The log_2_-fold change (LFC) value pF vs. aL in LAV (**A**) and in VRS (**B**) is the log_2_ of (gene abundance of pF/gene abundance of aL). The LFC value LAV vs. VRS is the log_2_ of (gene abundance of LAV_aL/gene abundance of VRS_aL) for aL (**C**) and the log_2_ of (gene abundance of LAV_pF/gene abundance of VRS_pF) for pF (**D**). For each pairwise comparison, only genes with a base mean > 20, a relative abundance > 0.005, p < 0.01, and LFC > 2.5 were selected. A list of all selected genes with their relative abundances, eggNOG classifications, and associated processes are provided in Additional file 1, Tables S5-8

**Fig. 5:**
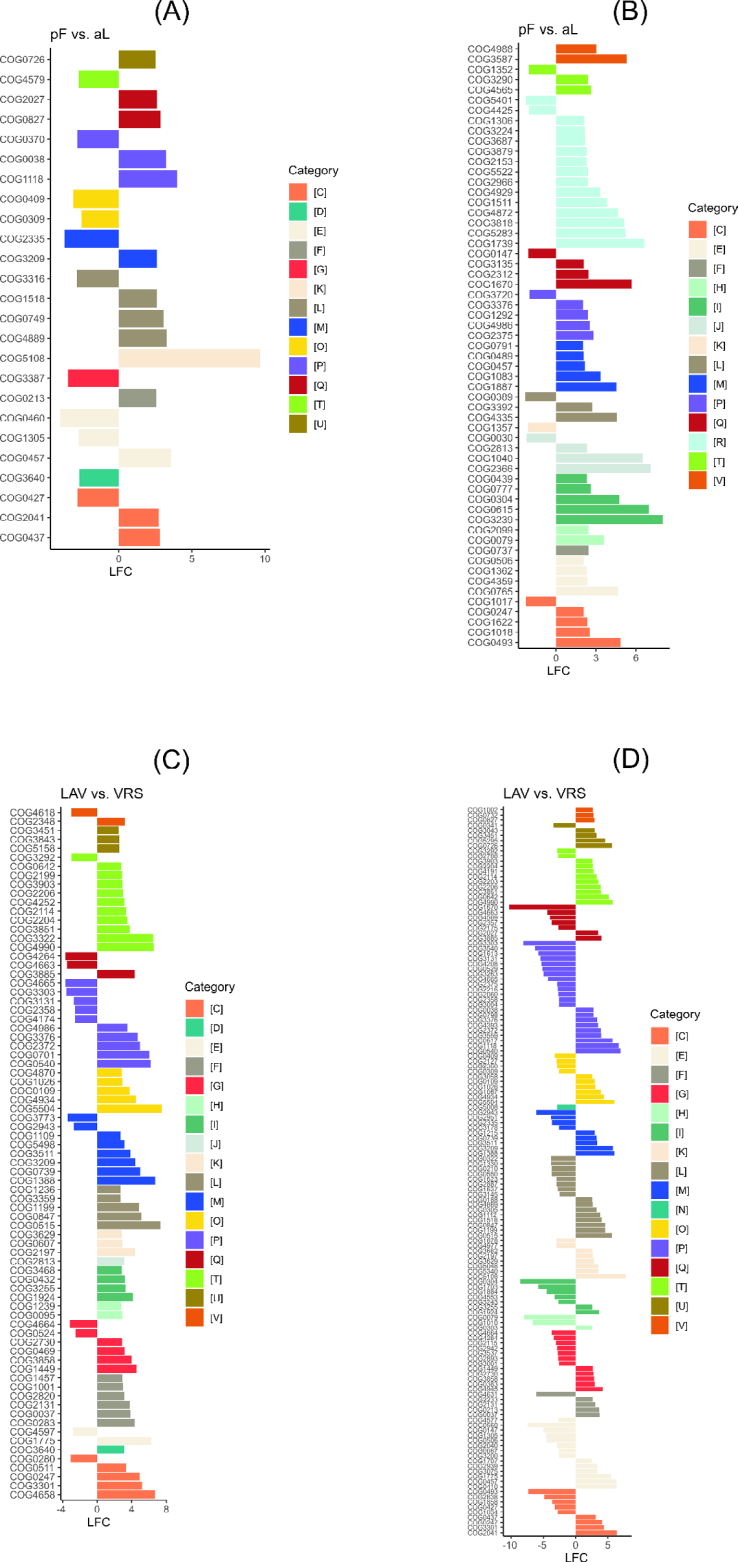
Differences in COG genes abundance between the two sampling sites and soil profiles LAV, Val Lavirun; VRS, Villum Research Station; aL, active layer; pF, permafrost The log_2_-fold change (LFC) value pF vs. aL in LAV (**A**) and in VRS (**B**) is the log_2_ of (gene abundance of pF/gene abundance of aL). The LFC value LAV vs. VRS is the log_2_ of (gene abundance of LAV_aL/gene abundance of VRS_aL) for aL (**C**) and the log_2_ of (gene abundance of LAV_pF/gene abundance of VRS_pF) for pF (**D**). For each pairwise comparison, only genes with a base mean > 20, a relative abundance > 0.005, p < 0.01, and LFC > 2.5 were selected. A list of all selected genes with their relative abundances, eggNOG classifications and associated processes are provided in Additional file 1, Tables S9-12

Regarding eggNOG functional categories, in VRS all categories were overrepresented in permafrost soil, except for transcription (K), which was overrepresented in the active layer ( Fig.4. and Additional File 1, Tables S5-8). The transcription functional descriptions involved the transcription of DNA into RNA (COG5108 in LAV) and the WD-40 repeat-containing protein (COG1357 in VRS) (Fig. 5. and Additional File 1, Tables S9-12).

The functional categories with the highest differential abundance (i.e. overrepresented) in permafrost soil in VRS were lipid transport and metabolism (I), and defense mechanisms (V). The functional descriptions involved: fatty acid desaturase (COG3239) and synthase, glycerol-3-phosphate cytidylyltransferase (COG0615) and carboxylase (I); ABC transporter (COG4988) and restriction enzyme (COG3587) (V) (Fig. 5 and Additional File 1, Tables S5-8; Fig. 5 and Additional File 1, Tables S9-12).

The most abundant overrepresented functional categories in the permafrost in LAV were transcription (K) and secondary metabolite biosynthesis, transport, and catabolism (Q; functional descriptions: deacylase [COG2027] and restriction-modification methylase [COG0827]). On the other hand, highly differentially abundant functional categories in the active layer in LAV were carbohydrate transport and metabolism (G; functional descriptions: glucan 1,4-alpha-glucosidase and hydrolase) and post-translational modification, protein turnover, and chaperones (O; functional descriptions: hydrogenase expression formation protein and air-synthase-related protein) (Fig. 4 and Additional File 1, Tables S5-8; Fig. 5 and Additional File 1, Tables S9-12).

In the comparison of the A horizon between LAV and VRS, all the functional categories were overrepresented in LAV, except for secondary metabolite biosynthesis, transport, and catabolism (Q; functional descriptions: extracellular solute-binding protein and siderophore biosynthesis), which was overrepresented in VRS. In the active layer of LAV, the most abundant overrepresented functional categories were replication, recombination and repair (L) and post-translational modification, protein turnover, and chaperones (O) (functional descriptions with highest LFCs: serine threonine protein kinase, DNA polymerase, predicted Zn-dependent protease, and pro-kumamolisin activation domain). In the comparison of the permafrost between the two sites, defense mechanisms (V; functional descriptions: DNA methylase and specificity) and intracellular trafficking, secretion, and vesicular transport (U) (functional descriptions with highest LFCs: virulence factor, induction of mutagenesis protein, domain protein/choline binding protein, histidine, and tyrosine kinase) were the most abundant functional categories overrepresented in LAV. The functional categories more abundant in VRS were coenzyme transport and metabolism (H) (functional descriptions with the highest LFC: catalyzes amidation of carboxylic groups of either cobyrinic acid or hydrogenobrynic acid) and cell motility (N; functional description: twitching motility protein) (Fig. 4 and Additional File 1, Tables S5-8; Fig. 5 and Additional File 1, Tables S9-12).

### Genes encoding carbohydrate-active enzymes

**Fig. 6:**
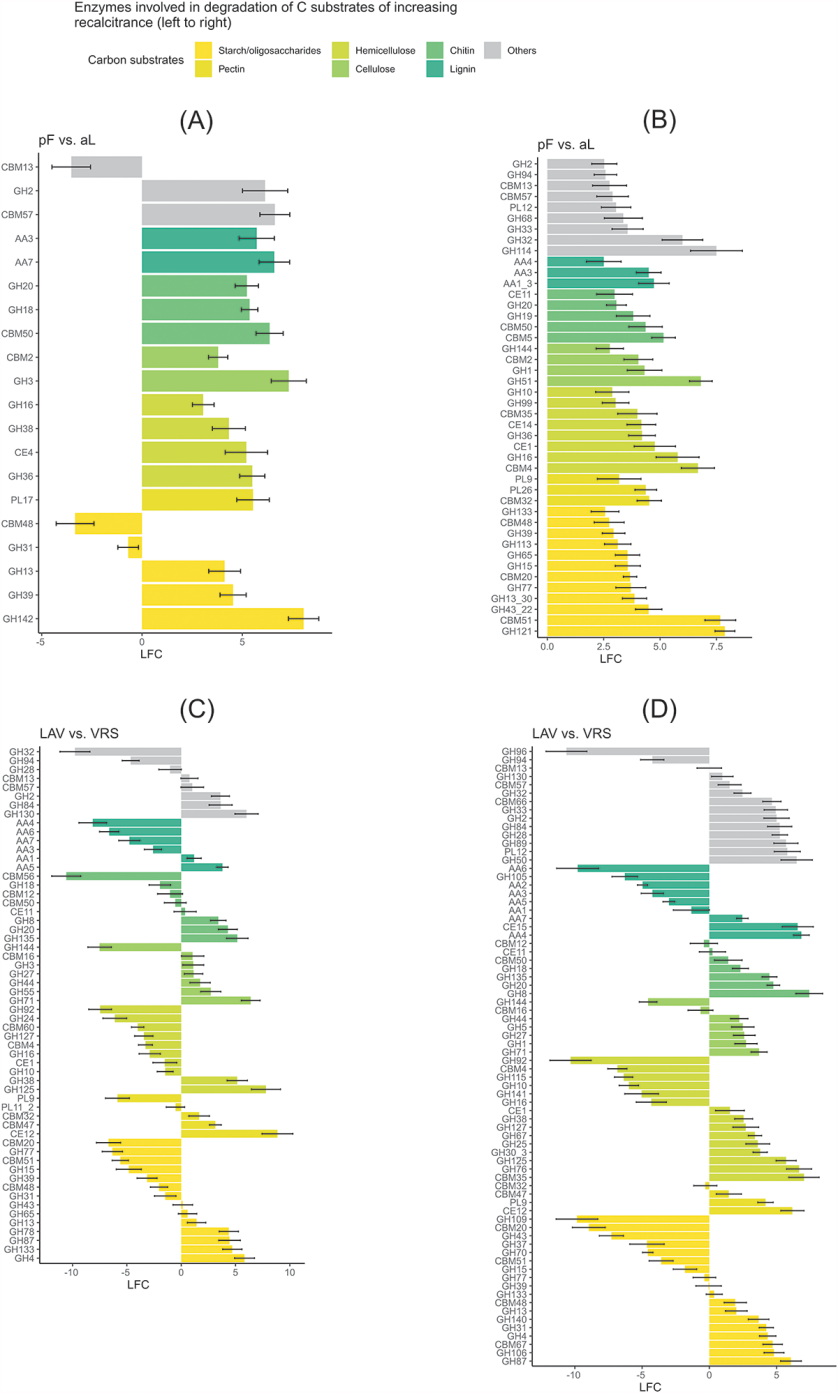
Differences in CAZy genes between the two sampling sites and soil profiles LAV, Val Lavirun; VRS, Villum Research Station; aL, active layer; pF, permafrost The log_2_-fold change (LFC) value pF vs. aL in LAV (**A**) and in VRS (**B**) is the log_2_ of (gene abundance of pF/gene abundance of aL). The LFC value LAV vs. VRS is the log_2_ of (gene abundance of LAV_aL/gene abundance of VRS_aL) for aL (**C**) and the log_2_ of (gene abundance of LAV_pF/gene abundance of VRS_pF) for pF (**D**). For each pairwise comparison, only genes with a base mean > 50, p < 0.01, and LFC > 2.5 were selected; the genes are sorted by their functions in the depolymerization of carbon substrates. Glycosyl transferases are not shown. A list of all selected genes with their relative abundances, CAZy classifications and associated enzymatic activities is provided in Additional file 1, Tables S13-16. [AA], auxiliary activities; [CBM], carbohydrate-binding modules; [CE], carbohydrate esterases; [GH], glycoside hydrolases; [PL], polysaccharide lyases

Overall, the most abundant CAZy genes, encoding carbohydrate-active enzymes, were in the category of glycoside hydrolases (GH), followed by auxiliary activity (AA) enzymes, carbohydrate esterases (CE), and polysaccharide lyases (PL). These genes are mainly involved in the degradation of starch and other oligosaccharides, followed by the degradation of hemicellulose, chitin, cellulose, and lignin. Only a few genes were involved in the degradation of pectin (Fig. 6).

The comparison between permafrost and active-layer soils in LAV and VRS indicated 2385 and 1073 differentially abundant genes (p < 0.01), respectively. In both sites, there were more overrepresented genes in permafrost than in the active layer (1308 vs. 1077 in LAV and 831 vs. 242 in VRS; Fig. 6 and Additional File 1, Table S4). In the GH family, the genes with the highest positive LFC in permafrost soils were involved in the activity of β-L-arabinobiosidase (GH121 and GH142; involved in oligosaccharide degradation). In the AA family, the main activities involved oxidase (AA7; lignin), cellobiose dehydrogenase (AA3; lignin), and laccase (AA1-3; lignin). Carbohydrate-binding modules (CBM) were also overrepresented in the permafrost in LAV and VRS, and the genes were mainly involved in the binding of galactose (CBM51) and the binding of xylan and glucan (CBM4) (Fig. 6 and Additional File 1, Tables S13-16).

In the comparisons of permafrost and active-layer soil between LAV and VRS, there were 3853 and 4733 differentially abundant genes (p < 0.01), respectively (Additional File 1, Table S4 and Tables S13-16). CAZy genes were overrepresented in VRS (2443 vs. 1410 in permafrost and 2967 vs. 1766 in active layer). In the GH family, the genes with highest positive LFCs occurred in LAV and were involved in the activities of ∂-agarase (GH96) and mannosyl-oligosaccharide ∂-1,2-mannosidase (GH92; oligosaccharide degradation). In the AA family, most of the genes with the highest positive LFCs were involved in the activities of 1,4-benzoquinone reductase (AA6; lignin; overrepresented in LAV in both active-layer and permafrost soils), while genes related to vanillyl-alcohol oxidase (AA4; lignin) were overrepresented in the permafrost in LAV and in the active layer in VRS (Fig. 6). Carbohydrate-binding modules (CBMs) involved in the binding of β-1,3 glucan (CBM56) and in the binding of starch (CBM20) were overrepresented in LAV in active-layer soils, while the binding of xylan (CBM35) was overrepresented in VRS in permafrost (Fig. 6 and Additional File 1, Tables S13-16).

### N-cycling genes

The comparison of N-cycling genes (annotated with NCyc) between permafrost and active-layer soil in LAV and VRS indicated 348 and 209 differentially abundant genes (p < 0.01), respectively. In both sites, there were more genes with a positive LFC in permafrost than in the active layer (202 vs. 146 in LAV and 155 vs. 54 in VRS; Additional File 1, Table S4 and Tables S17-20). In the comparisons of permafrost and of active-layer soil between LAV and VRS, there were 864 and 790 differentially abundant genes (p < 0.01), respectively. N-cycling genes were most often overrepresented in VRS (516 vs. 348 in permafrost and 435 vs. 355 in active layer; Additional File 1, Table S4 and Tables S17-20).

**Fig. 7:**
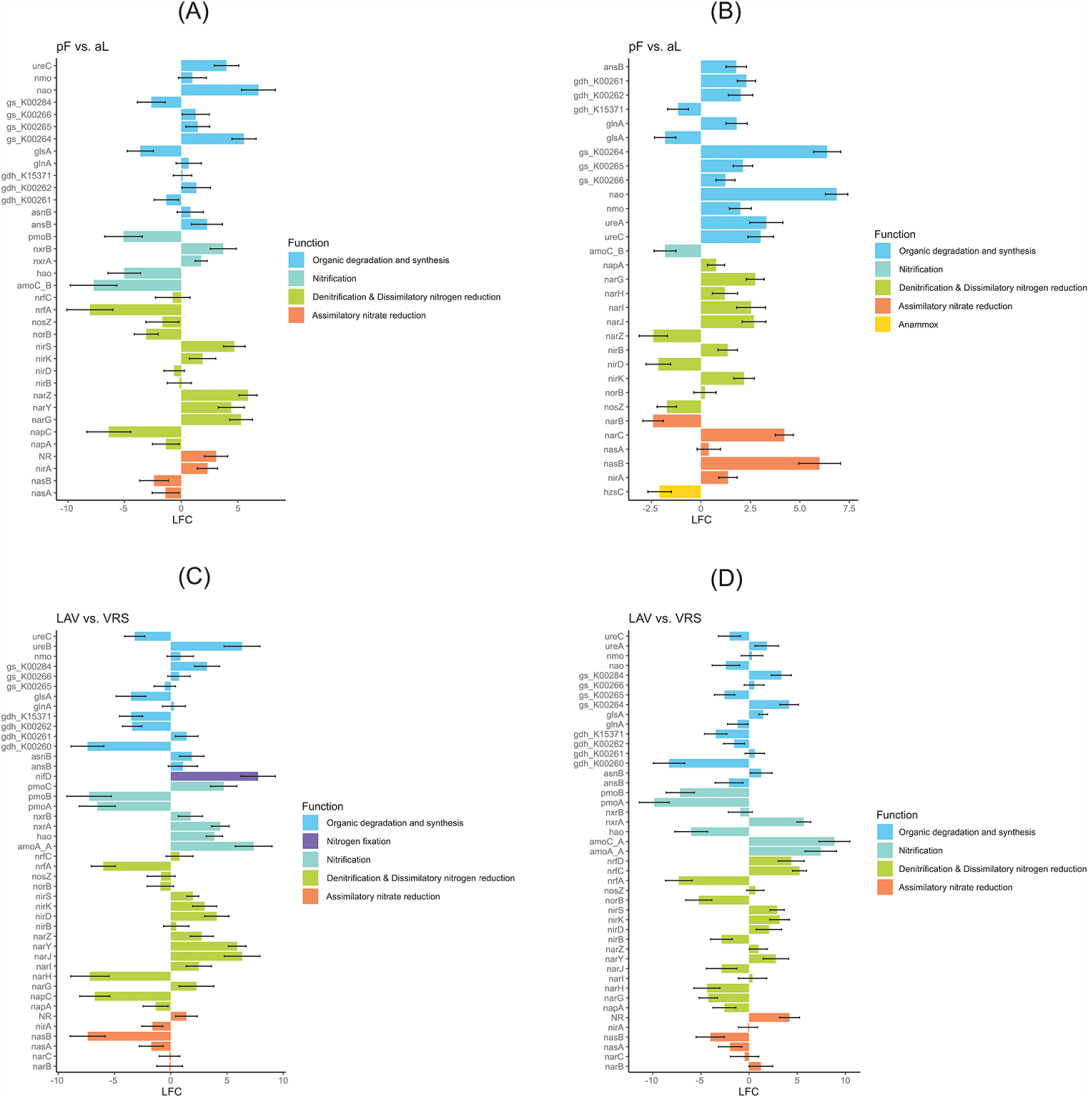
Differences in N-cycling gene families between the two sampling sites and soil profiles LAV, Val Lavirun; VRS, Villum Research Station; aL, active layer; pF, permafrost The log_2_-fold change (LFC) value pF vs. aL in LAV (**A**) and in VRS (**B**) is the log_2_ of (gene abundance of pF/gene abundance of aL). The LFC value LAV vs. VRS is the log_2_ of (gene abundance of LAV_aL/gene abundance of VRS_aL) for aL (**C**) and the log_2_ of (gene abundance of LAV_pF/gene abundance of VRS_pF) for pF (**D**). For each pairwise comparison, only genes with a base mean > 50 and p < 0.01 were selected. A list of all selected genes with their relative abundances, NCyc classifications, and associated processes is provided in Additional file 1, Tables S17-20

Overall, the most abundant N-cycling families were involved in the activities of organic degradation and synthesis (ODS), followed by denitrification and dissimilatory nitrate reduction (DNR), assimilatory nitrate reduction (ANR), and nitrification (NT). The families annamox (*hzsC*) and N fixation (*nifD*) were overrepresented only in the active layer in VRS and only in the active layer in LAV, respectively (Fig. 7). In the comparisons between permafrost and active-layer soils, ODS was overrepresented overall in permafrost in both LAV and VRS. The genes *ureC*, *nao*, and *gs_K00265* were overrepresented in permafrost in both sites, while the gene *glsA* was overrepresented in the active layer in both sites. In the denitrification and DNR family, the genes *nirK* and *narG* were overrepresented in permafrost in both LAV and VRS. In LAV the genes with the higher LFCs in permafrost were *nirS*, *narY*, and *narZ*, while *narI* and *narJ* had the higher LFCs in permafrost in VRS. In the nitrification family, *amoC_B* was overrepresented in the active layer in LAV and was the only overrepresented gene in the active layer of VRS. In the ANR family, *nirA* was overrepresented in the permafrost of both localities, while *nasB* was overrepresented in the active layer in LAV but in permafrost in VRS (Fig. 7 and Additional File 1, Tables S17-20).

In the comparisons of permafrost and of active-layer soil between LAV and VRS, the genes with the highest positive LFCs (overrepresented in LAV) were *ureB* (in permafrost), *gs-K00264* (in the active layer) and *gs-K00284* (in both soil layers), while *ureC* and *gdh-K00260* were overrepresented in VRS in both soil layers. In the denitrification and DNR family, *narY* and *narJ* (in the active layer), and *nrfD* and *nrfC* (in permafrost) were overrepresented in LAV. In the nitrification family, *amoA_A* and *pmoC* (in the active layer) and *amoC_A* and *amoA_A* (in permafrost) were overrepresented in LAV, while *pmoA* and *pmoB* (in both soil layers) were overrepresented in VRS. In the ANR family, NR was overrepresented in LAV while *nasB* was overrepresented in VRS, in both soil layers in each case (Fig. 7 and Additional File 1, Tables S17-20).

## Discussion

In agreement with our first hypothesis, this study shows that the microbial community structure differed between LAV and VRS soils in terms of both alpha- and beta-diversity. These differences were strongly governed by the soil physico-chemical properties, which might largely be attributed to differences in biotic activity [18]. The main abiotic differences were the lower pH and the lower C content in LAV soils. Some studies have indicated that microbial abundance and diversity are related to resource availability, and significant correlations between the microbiota and soil C content have been reported [81–84]. The low pH and C content in LAV soils led to highly structured bacterial communities, with a high abundance of the Chloroflexi-AD3 group (both in the metabarcoding and shotgun metagenomic analyses). It has been reported previously that AD3 (Candidatus Dormibacteraeota; dormant bacterial phylum) thrives in soil with a low pH and C content [28] and is a common bacterial group in permafrost habitats [18, 85, 86]. Based on assembled genomes representative of this candidate phylum, functions that are likely to be beneficial in nutrient-poor environments include the synthesis and storage of carbohydrates, the potential to use carbon monoxide (CO) as a supplemental energy source, and the ability to form spores [87]. Together, these functions likely enable members of the candidate phylum Dormibacteraeota to flourish in high-alpine soils and provide insight into the survival and growth strategies employed by the microbes that thrive in oligotrophic soil environments [32, 88].

Interestingly, we found high abundances of unclassified bacteria (by metabarcoding analysis) and unclassified predicted genes (by Kaiju analysis) in both sites, suggesting that our knowledge of permafrost soils is still very limited. The rapid degradation of permafrost soils as a consequence of global warming may lead to extinction of this unexplored microbial diversity and potential release of putative unknown pathogenic microorganisms into the environment [18, 21, 89, 90]. In disagreement with our first hypothesis, a high abundance of Ascomycota was found in both sites. This finding is in line with a previous metabarcoding analysis in permafrost and in cold environments [18, 41, 91–94]. Likewise, the high abundance of Mortierellomycota (represented by the genus *Mortierella*, mainly in LAV) is not surprising. *Mortierella* spp. have been reported to survive in cold environments by changing the composition of lipid membranes and increasing levels of unsaturated fatty acids and by trehalose accumulation for protection against the adverse effects of low temperatures [95].

The differences in taxonomic composition between LAV and VRS could also be partly attributed to the different ages of the permafrost soils. Recently, it was reported that the age of samples could be a key factor governing microbial community structure in permafrost habitats: microbial composition changes with increasing permafrost age, with a corresponding increase of genes related to survival strategies [36]. Therefore, permafrost age can act as an environmental pressure, selecting for a subset of species which may change at the time of permafrost formation [36, 96, 97].

Although the structure of microbial communities has been reported to be mainly affected by permafrost age, ice content, dispersal limitation, and physical/thermodynamic constraints, rather than edaphic parameters [16, 36, 97, 98], physical and chemical aspects can still have a substantial influence on the alpha-, beta-, and functional diversity of soil microorganisms [81, 99, 100]. In this regard, while fungal communities at our two study sites were previously found to be affected by edaphic factors (i.e. depth, pH, C and N content; [41, 81–83]), bacteria were affected by all abiotic parameters tested except for water content. Generally, there is low water activity in permafrost soils [101] and microorganisms are more halotolerant than in the overlaying active layer [102], suggesting that a halophilic lifestyle could be one of the several microbial survival strategies for both bacterial and fungal communities. However, the correlations between all abiotic parameters and bacteria could explain the high abundance of this microbial group found by shotgun sequencing analysis.

Ancient soils like the ones in VRS consist of relic DNA which can be abundant in soils [103, 104]. There is debate as to whether the presence of relic DNA fundamentally alters estimates of microbial diversity [105]. Our DNA-based study includes active, dead, and dormant cells. Despite in this work we did not differentiate between active, dormant, and dead cells present in the permafrost-affected soils we assume that the spatial distribution of microorganisms observed here likely represents a realistic scenario of microbial communities in those niches over the past period. This scenario is supported by recent studies from permafrost environments. First, [98] did not find a significant change in microbial community structure after removal of relict DNA preserved within Beringian permafrost. Second, [106] did not report any statistically significant variation in the microbial diversity between Holocene and Pleistocene samples within a permafrost core extracted from central Yukon, a part of Eastern Beringia.

Whole-community shotgun metagenomics sequencing can be considered a powerful tool to characterize the metabolic potentials of microbial communities, including those in extreme environments. However, microbial gene expression cannot be captured using a metagenomic approach but only indirectly inferred. Therefore, in this study, the potential of functional profiles of active layer and permafrost soils of alpine and High Arctic sites was investigated using shotgun metagenomics. Overall, functional gene diversity based on the EggNOG, CAZy, and NCyc datasets indicated differences between the two localities and between permafrost and active-layer soil.

The functional genetic potential of microbial communities can vary significantly between alpine and High Arctic regions. Such differences may have important ecological implications for microbial survival and energy production. Arctic soils are characterized by low temperatures and late Pleistocene-aged soils, which select for microbial communities that are adapted to cold and nutrient-poor conditions [107]. These communities may have a higher proportion of genes related to stress response and nutrient acquisition, such as those involved in cold shock and transport of low molecular weight compounds [108, 109]. Such adaptations allow them to cope with these harsh environments where other organisms may not survive. In contrast, the low pH and carbon content present in the alpine site can also influence microbial community composition and function. The microbiomes in these soils have a higher proportion of genes related to acid tolerance and alternative energy production, such as sulfur oxidation, methanogenesis, or fermentation [42, 110]. These adaptations help them to contend with the low pH and lack of organic matter by utilizing alternative energy sources [87].

Overall, the functional genetic potential of microbial communities in different High Arctic and alpine soils can have important implications for their ability to survive and thrive in their respective environments. Understanding these differences provide further insights how permafrost-affected microbial communities shape ecosystem biogeochemistry in the context of global change.

The functional genetic potential of microbial communities can also differ between the seasonally active layer and the permafrost layer in regions, with important ecological implications in a warmed Arctic and Alps. For instance, the organisms that inhabit both the active layer and the permafrost soil will respond differently to a warmer climate based on where in the soil they are present and the soil characteristics. In the seasonally active layer, microbial communities have access to more labile organic matter, which is more easily degradable than the old carbon found in the permafrost soils. As a result, these communities may have a higher proportion of genes related to carbohydrate metabolism and organic matter degradation, which contribute to C- and N cycling in the soil [111]. In contrast, permafrost contains more recalcitrant organic matter that has been frozen for long periods of time. Microbial communities in this layer may have a higher proportion of genes related to stress response and survival, such as those involved in DNA repair and cold shock proteins [112]. However, microbiomes may also have a lower functional diversity due to the limited availability of organic matter and nutrients.

In agreement with our second hypothesis, the functional genes annotated against EggNOG indicated that most of the predicted genes in permafrost soil in VRS were categorized as cell metabolism (lipid transport by fatty acid desaturase) and defense mechanisms (ABC transporters). Fatty acids are essential components of microbial membranes and are important for environmental stress responses and survival. In response to cold conditions, microorganisms produce a desaturase that modifies existing saturated fatty acids in the membrane to form monounsaturated fatty acids. Their increase in membranes leads to increased membrane fluidity, preventing the microorganisms from freezing [113]. ABC transporters are responsible for the uptake and secretion of a wide range of substrates (e.g. ions, amino acids, sugars), and they are implicated in several cellular processes, including defense mechanisms such as xenobiotic protection, bacterial immunity, and virulence [114]. Moreover, the presence of ABC transporters should confer resistance to *skf* operons (sporulation killing factor), which are present in the initial stages of sporulation [115]. Therefore, ABC transporters facilitate the sporulation process, while dormancy of this process may represent a reliable survival strategy for bacteria inhabiting permafrost ecosystems [41].

Permafrost soils in alpine and Arctic regions may share some common metabolic pathways and gene functions due to their similar environmental conditions. In particular, permafrost soils in both regions contain old carbon that is often stored for long periods due to the cold temperatures and the microorganisms of these soils have adapted to utilize this carbon through various metabolic pathways [116]. However, in agreement with our third hypothesis, differences in C-cycling genes were found between permafrost and active-layer soils. The diversity of CAZy genes was higher in active-layer soils than in permafrost in both localities, supporting findings by [117]. However, overrepresented CAZy genes were found in permafrost soils. In warmer soils, increases in genes involved in the degradation of C substrates suggest the possible decomposition of labile and recalcitrant C compounds. These findings suggest that frozen soil becomes biologically active during permafrost thawing, with the consequence of increased C loss from the environment due to increased microbial activity [17]. Moreover, the many overrepresented CAZy genes in permafrost soils may indicate a high microbial genetic potential for the degradation of complex C substrates. The decomposition of C compounds (i.e. oligosaccharides, cellulose, hemicellulose, and lignin) in the deepest frozen layers, where oxygen and nutrients are lacking, produces easily degradable carbohydrates that can support microbial survival and growth [33, 118].

In disagreement with our hypothesis, commonalities in N-cycling genes were found between permafrost and active-layer soils. High latitude (Arctic sites) and high-altitude (alpine sites) ecosystems are considered N-limited, given the small amount of soluble N compounds [62, 119, 120], resulting in competition for N between plants and microorganisms. However, mineral N cycling occurs in the active layer of permafrost-affected soils to a similar extent as in temperate or even tropical soils, and its main processes (ammonification and nitrification) are similarly dependent on C and N availability [121]. Overrepresented genes involved in the N cycle in active-layer soils of LAV and VRS were related to anammox (*hzsC*) and N fixation (*nifD*). Biological N fixation can contribute significantly to N availability (in the form of ammonia [NH_3_] and ammonium [NH_4_^+^]) in ecosystems with permafrost [21, 62, 122, 123], while the high abundance of anammox (able to oxidize NH_3_ and reduce nitrite [NO_2_^−^] to N_2_ gas) suggests a possible reduction in the emission of the greenhouse gases NO_2_ and CO_2_ [124].

Overrepresented N-cycling genes in permafrost soil were mainly involved in the biodegradation of N compounds (*ureC* and *nao*). Urease is one of the most important enzymes in the N cycle [125]. Urea is an N source hydrolyzed in NH_3_ and carbamate as an energy and C source [126]. Bacterial urease is a trimer of three subunits, encoded by *ureA, ureB*, and *ureC*, with the *ureC* gene considered the target gene for urease analysis because it is the largest of the genes encoding urease functional subunits [127].

High positive LFC values for genes involved in denitrification processes were found in permafrost in both LAV and VRS. The potential for denitrification is well documented in permafrost-affected soils. In particular, in the High Arctic and alpine soils considered in this study, the high abundance of genes involved in denitrification processes have been reported previously [21, 61, 62], and global warming has been shown to increase their abundance [128, 129].

As the Arctic and Alps continue to warm, the permafrost layers may thaw, releasing previously frozen organic matter and nutrients into the seasonally active layer. This can stimulate microbial activity and lead to increased greenhouse gas emissions, such as carbon dioxide and methane, from the soil. The functional genetic potential of permafrost-affected microbial communities in these regions will be important in determining the extent of these emissions and the impact of a warming climate on ecosystem function.

## Conclusion

In the present study, we reported differences in the functional gene diversity and the metabolic potential between alpine and High Arctic permafrost-affected soil microbiomes. In such extreme environments, microorganisms have adapted to the cold through survival strategies that take essential genes into account. The ability of permafrost microorganisms to survive at sub-zero temperatures, their energetic strategies, and their metabolic versatility in using soil organic materials determine their growth and functionality upon thawing. Here, we found differences in general metabolic and cellular functions between the alpine and High Arctic soils. In particular, a higher abundance of genes involved in the survival of microorganisms under freezing conditions was found in the High Arctic soils.

Permafrost thawing supports the release of organic elements, promoting the metabolic rates of microbial decomposers and the consequent release of greenhouse gases. In this regard, we found significant abundances of microbial genes involved in the C and N cycles in the High Arctic and alpine permafrost soils. Microorganisms are directly responsible for organic matter decomposition and greenhouse gas emission, so attention to their functional genes is essential because they can be expected to shift considerably with additional temperature increases and new soil formation. Therefore, the functional characterization of the permafrost microbiome, particularly in the underexplored mid-latitudinal alpine regions, is a crucial step in predicting its response to the changing climate and potential soil–climate feedbacks.

## Electronic supplementary material

Below is the link to the electronic supplementary material.

**Table S1**. Relative abundance of Archaea in alpine (Val Lavirun, LAV) and High Arctic soils (Villum Research Station, VRS) along the soil profile. **Table S2**. Results of envfit analyses (A, bacteria; B, fungi) testing the correlation between environmental parameters and the soil microbial communities in the non-metric multidimensional scaling (NMDS) ordination. Depth, soil depth; T °C, soil temperature; C, carbon; N, nitrogen; DOC, dissolved organic carbon; DN, dissolved nitrogen; Water, gravimetric water content; ^14^C, ^14^C radiocarbon. Significant (< 0.05) p values are shown in bold (number of permutations 999). **Table S3**. Overall assembly statistics of the metagenomic data. **Table S4**. Numbers of functional genes annotated using eggNOG (only with COG ID/annotations), CAZy, and NCyc that differed significantly between the different soils in Val Lavirun (LAV, alpine site) and Villum Research Station (VRS, High Arctic site). Four different comparisons were considered: (i) permafrost (pF) in LAV vs. active layer (aL) in LAV; (ii) pF in VRS vs. aL in VRS; (iii) aL in LAV vs. aL in VRS; (iv) pF in LAV vs. pF in VRS. A horizon (0 ? 5 cm depth) was considered active layer here. **Table S5**. Functional annotation, log2 fold change values and relative abundance of differentially abundant COG genes selected in the study (related to Figure 4A). LAV, Val Lavirun; pF, permafrost; aL, active layer; SE, standard error; Rel.ab, relative abundance. **Table S6**. Functional annotation, log2 fold change values and relative abundance of differentially abundant COG genes selected in the study (related to Figure 4B). VRS, Villum Research Station; pF, permafrost; aL, active layer; SE, standard error; Rel.ab, relative abundance. **Table S7**. Functional annotation, log2 fold change values and relative abundance of differentially abundant COG genes selected in the study (related to Figure 4C). aL, active layer; LAV, Val Lavriun; VRS, Villum Research Station; SE, standard error; Rel.ab, relative abundance. **Table S8**. Functional annotation, log2 fold change values and relative abundance of differentially abundant COG genes selected in the study (related to Figure 4D). pF, permafrost; LAV, Val Lavriun; VRS, Villum Research Station; SE, standard error; Rel.ab, relative abundance. **Table S9**. Functional annotation, log2 fold change values and relative abundance of differentially abundant COG genes selected in the study (related to Figure 5A). LAV, Val Lavirun; pF, permafrost; aL, active layer; Rel.ab, relative abundance. **Table S10**. Functional annotation, log2 fold change values and relative abundance of differentially abundant COG genes selected in the study (related to Figure 5B). VRS, Villum Research Station; pF, permafrost; aL, active layer; Rel.ab, relative abundance. **Table S11**. Functional annotation, log2 fold change values and relative abundance of differentially abundant COG genes selected in the study (related to Figure 5C). aL, active layer; LAV, Val Lavriun; VRS, Villum Research Station; Rel.ab, relative abundance. **Table S12**. Functional annotation, log2 fold change values and relative abundance of differentially abundant COG genes selected in the study (related to Figure 5D). pF, permafrost; LAV, Val Lavriun; VRS, Villum Research Station; Rel.ab, relative abundance. **Table S13**. Functional annotation, log2 fold change values and relative abundance of differentially abundant CAZy genes selected in the study (related to Figure 6A). LAV, Val Lavirun; pF, permafrost; aL, active layer; SE, standard error; Rel.ab, relative abundance. **Table S14**. Functional annotation, log2 fold change values and relative abundance of differentially abundant CAZy genes selected in the study (related to Figure 6B). VRS, Villum Research Station; pF, permafrost; aL, active layer; SE, standard error; Rel.ab, relative abundance. **Table S15**. Functional annotation, log2 fold change values and relative abundance of differentially abundant CAZy genes selected in the study (related to Figure 6C). aL, active layer; LAV, Val Lavirun; VRS, Villum Research Station; SE, standard error; Rel.ab, relative abundance. **Table S16**. Functional annotation, log2 fold change values and relative abundance of differentially abundant CAZy genes selected in the study (related to Figure 6D). pF, permafrost; LAV, Val Lavirun; VRS, Villum Research Station; SE, standard error; Rel.ab, relative abundance. **Table S17**. Functional annotation, log2 fold change values and relative abundance of differentially abundant NCYc genes selected in the study (related to Figure 7A). LAV, Val Lavirun; pF, permafrost; aL, active layer; SE, standard error; Rel.ab, relative abundance. **Table S18**. Functional annotation, log2 fold change values and relative abundance of differentially abundant NCYc genes selected in the study (relative to Figure 7B). VRS, Villum Research Station; pF, permafrost; aL, active layer; SE, standard error; Rel.ab, relative abundance. **Table S19**. Functional annotation, log2 fold change values and relative abundance of differentially abundant NCYc genes selected in the study (relative to Figure 7C). aL, active layer; LAV, Val Lavirun; VRS, Villum Research Station; SE, standard error; Rel.ab, relative abundance. **Table S20**. Functional annotation, log2 fold change values and relative abundance of differentially abundant NCYc genes selected in the study (relative to Figure 7D). pF, permafrost; LAV, Val Lavirun; VRS, Villum Research Station; SE, standard error; Rel.ab, relative abundance.

**Supplementary Figure 1**. Relative abundance of the most abundant taxa at the domain level in active-layer (aL) and permafrost soils (pF). In each panel the Val Lavirun alpine site (LAV) is on the left side and the Villum Research Station High Arctic site (VRS) is on the right side. 16S/18S rRNA genes (A) were assigned to the SILVA taxonomy database v138. Predicted genes (B), eggNOG genes (C), CAZy genes (D), and NCyC (E) were assigned to the NCBI taxonomy with Kaiju v1.7.4. Relative abundance of the most abundant taxa at the phylum level (F). **Supplementary Figure 2**. Shannon-H diversity index based on the read abundance of different genes. **Supplementary Figure 3**. Functional structure of genes annotated to the different databases in alpine (Val Lavirun, LAV) and High Arctic (Villum Research Station, VRS) soil samples. Samples are visualized by principal coordinate analysis (PCoA). (A): eggNOG; (B): CAZy; (C): NCyc. aL, active layer; pF, permafrost.

## Data Availability

The data that support the findings of this study are openly available in NCBI Sequence Read Archive under the BioProject numbers PRJNA917658 (amplicon metagenomics; https://www.ncbi.nlm.nih.gov/bioproject/PRJNA917658) and PRJNA917667 (shotgun metagenomics; https://www.ncbi.nlm.nih.gov/bioproject/PRJNA917667.)
