# Supplementary material for: Distinct taxonomic and functional profiles of high Arctic and alpine permafrost-affected soil microbiomes
Source: Environ Microbiome. 2023 Jun 16;18:54. doi: 10.1186/s40793-023-00509-6 (PMC10276392; doi:10.1186/s40793-023-00509-6)
Supplement: Supplementary file 1 — Table S1. Relative abundance of Archaea in alpine (Val Lavirun, LAV) and High Arctic soils (Villum Research Station, VRS) along the soil profile. Table S2. Results of envfit analyses (A, bacteria; B, fungi) testing the correlation between environmental parameters and the soil microbial communities in the non-metric multidimensional scaling (NMDS) ordination. Depth, soil depth; T °C, soil temperature; C, carbon; N, nitrogen; DOC, dissolved organic carbon; DN, dissolved nitrogen; Water, gravimetric water content; 14C, 14C radiocarbon. Significant (< 0.05) p values are shown in bold (number of permutations 999). Table S3. Overall assembly statistics of the metagenomic data. Table S4. Numbers of functional genes annotated using eggNOG (only with COG ID/annotations), CAZy, and NCyc that differed significantly between the different soils in Val Lavirun (LAV, alpine site) and Villum Research Station (VRS, High Arctic site). Four different comparisons were considered: (i) permafrost (pF) in LAV vs. active layer (aL) in LAV; (ii) pF in VRS vs. aL in VRS; (iii) aL in LAV vs. aL in VRS; (iv) pF in LAV vs. pF in VRS. A horizon (0 ? 5 cm depth) was considered active layer here. Table S5. Functional annotation, log2 fold change values and relative abundance of differentially abundant COG genes selected in the study (related to Figure 4A). LAV, Val Lavirun; pF, permafrost; aL, active layer; SE, standard error; Rel.ab, relative abundance. Table S6. Functional annotation, log2 fold change values and relative abundance of differentially abundant COG genes selected in the study (related to Figure 4B). VRS, Villum Research Station; pF, permafrost; aL, active layer; SE, standard error; Rel.ab, relative abundance. Table S7. Functional annotation, log2 fold change values and relative abundance of differentially abundant COG genes selected in the study (related to Figure 4C). aL, active layer; LAV, Val Lavriun; VRS, Villum Research Station; SE, standard error; Rel.ab, relative abundance. T [file 40793_2023_509_MOESM1_ESM.docx]

**Table S1**. Relative abundance of Archaea in alpine (Val Lavirun, LAV) and High Arctic soils (Villum Research Station, VRS) along the soil profile.

|  | **LAV** | | | |  | **VRS** | | | | |
| --- | --- | --- | --- | --- | --- | --- | --- | --- | --- | --- |
| **Phylum** | aL_A | aL_B | aL_B_c_ | pF |  | BSC | aL_A | aL_B | aL_B_c_ | pF |
| Crenarchaeota | 94.5% | 100% | 100% | 100% |  | 98.0% | 90.2% | 82.2% | 76.2% | 98.2% |
| Micrarchaeota | 0.09% | 0 | 0 | 0 |  | 0 | 0 | 0 | 0 | 0 |
| Nanoarchaeota | 0 | 0 | 0 | 0 |  | 0 | 0.09% | 0 | 0 | 0 |
| Thermoplasmatota | 5.4% | 0 | 0 | 0 |  | 2.0% | 9.7% | 17.8% | 23.8% | 1.8% |

BSC = biological soil crust; aL = active-layer soils; pF = permafrost soils.

A = A horizon enriched with organic matter and thus darker than the underlying B horizon.

B = B horizon.

Bc = transitional layer with characteristics of both B and C horizons, with B horizon characteristics dominant; partly affected by cryoturbation, as manifested by a transition of horizons.

**Table S2**. Results of envfit analyses (A, bacteria; B, fungi) testing the correlation between environmental parameters and the soil microbial communities in the non-metric multidimensional scaling (NMDS) ordination. Depth, soil depth; T °C, soil temperature; C, carbon; N, nitrogen; DOC, dissolved organic carbon; DN, dissolved nitrogen; Water, gravimetric water content; ^14^C, ^14^C radiocarbon. Significant (< 0.05) p values are shown in bold (number of permutations 999).

|  | **Factor** | **NMDS1** | **NMDS2** | **r^2^** | **p** |
| --- | --- | --- | --- | --- | --- |
| A | Depth | -0.000341 | 1.000000 | 0.4645 | **0.001** |
|  | T °C | 0.000197 | -1.000000 | 0.5763 | **0.001** |
|  | pH | 0.055929 | -0.998430 | 0.9707 | **0.001** |
|  | C | 0.000366 | -1.000000 | 0.5928 | **0.001** |
|  | N | 0.000694 | -1.000000 | 0.7378 | **0.001** |
|  | DOC | -0.000068 | -1.000000 | 0.3958 | **0.003** |
|  | DN | 0.000008 | -1.000000 | 0.5082 | **0.001** |
|  | Water | 0.000004 | 1.000000 | 0.1053 | 0.247 |
|  | ^14^C | 0.000354 | 1.000000 | 0.4270 | **0.002** |
|  |  |  |  |  |  |
| B | Depth | -0.45063 | 0.89271 | 0.3662 | **0.005** |
|  | T °C | 0.48694 | -0.87344 | 0.3344 | **0.008** |
|  | pH | 0.99945 | 0.03330 | 0.8191 | **0.001** |
|  | C | 0.93027 | -0.36688 | 0.4129 | **0.002** |
|  | N | 0.92486 | -0.38031 | 0.6623 | **0.001** |
|  | DOC | 0.35679 | -0.93418 | 0.0263 | 0.727 |
|  | DN | 0.8183 | -0.59260 | 0.0652 | 0.438 |
|  | Water | -0.08183 | 0.99665 | 0.0796 | 0.347 |
|  | ^14^C | 0.45138 | 0.89233 | 0.1351 | 0.168 |

**Table S3.** Overall assembly statistics of the metagenomic data.

**(A)**

| Item | Number |
| --- | --- |
| Contigs | 1,477,538 |
| Assembly size | 844,722,899 |
| Mean | 571 |
| Median | 3,807 |
| Maximum transcript length | 720,250 |
| Minimum transcript length | 200 |
| N50 | 897 |
| GC content (%) | 63 |
| Predicted genes | 3,952,470 |
| Predicted genes annotated with CAZy | 39,507 |
| Predicted genes annotated with NCyc | 6,373 |
| Predicted genes annotated with eggNOG | 2,000,785 |

**(B)**

|  | **Location (soil layer)** | | | |
| --- | --- | --- | --- | --- |
|  | **LAV (aL)** | **LAV (pF)** | **VRS (aL)** | **VRS (pF)** |
| Raw reads (×10^6^) | 20.2±3.9 | 15.0±8.8 | 22.8±2.8 | 16.2±4.7 |
| HQ reads (×10^6^) | 20.0±3.9 | 14.9±8.7 | 22.6±2.8 | 16.1±4.7 |
| No. reads mapped to CDS genes | (40.0±8.8) × 10^5^ | (3.5±2.3) × 10^6^ | (35.8±6.2) × 10^5^ | (20.0±4.4) × 10^5^ |
| % reads mapped to CDS genes | 20±0 | 23±2 | 16±1 | 13±1. |
| No. reads mapped to contigs | (6.1±1.5) × 10^6^ | (4.6±3.0) × 10^6^ | (5.9±1.2) × 10^6^ | (34.5±9.8) × 10^5^ |
| % reads mapped to contigs | 30±2 | 31±2 | 25±3 | 22±1 |

The total number of sequences and the percentage of protein-coding genes (CDS) and contigs in the alpine (Val Lavirun, LAV) and High Arctic (Villum, VRS) soils. aL = active layer; pF = permafrost.

**Table S4.** Numbers of functional genes annotated using eggNOG (only with COG ID/annotations), CAZy, and NCyc that differed significantly between the different soils in Val Lavirun (LAV, alpine site) and Villum Research Station (VRS, High Arctic site). Four different comparisons were considered: (i) permafrost (pF) in LAV vs. active layer (aL) in LAV; (ii) pF in VRS vs. aL in VRS; (iii) aL in LAV vs. aL in VRS; (iv) pF in LAV vs. pF in VRS. A horizon (0 – 5 cm depth) was considered active layer here.

|  |  | **LAV** | **VRS** | **aL** | **pF** |
| --- | --- | --- | --- | --- | --- |
|  |  | **pF vs. aL** | **pF vs. L** | **LAV vs. VRS** | **LAV vs. VRS** |
| **eggNOG** | Total | 1755 | 2039 | 2099 | 4292 |
|  | Up | 931 | 1632 | 1006 | 2271 |
|  | Down | 824 | 407 | 1093 | 2021 |
| **CAZy** | Total | 2385 | 1073 | 4733 | 3853 |
|  | Up | 1308 | 831 | 1766 | 1410 |
|  | Down | 1077 | 242 | 2967 | 2443 |
| **NCyc** | Total | 348 | 209 | 790 | 864 |
|  | Up | 202 | 155 | 355 | 348 |
|  | Down | 146 | 54 | 435 | 516 |

Total = total number of significantly different genes; *Up* = overrepresented; *Down* = underrepresented. For example, in the pF vs. aL column for LAV, *Up* means the number of genes that were more abundant (overrepresented) in the permafrost soils in LAV, while *Down* means the number of genes that were more abundant in the active layer in LAV.

**Table S5.** Functional annotation, log2 fold change values and relative abundance of differentially abundant COG genes selected in the study (related to Figure 4A). LAV, Val Lavirun; pF, permafrost; aL, active layer; SE, standard error; Rel.ab, relative abundance.

| **LAV** | **Functional_category** | **Log2FoldChange** | **SE** | **Rel.ab** |
| --- | --- | --- | --- | --- |
| pF vs aL | Amino acid transport and metabolism [E] | -1.062147206 | 1.220083 | 0.024774968 |
| pF vs aL | Carbohydrate transport and metabolism [G] | -3.486983978 | 0.322998 | 0.006315214 |
| pF vs aL | Cell cycle control, cell division, chromosome partitioning [D] | -2.710972409 | 0.311931 | 0.02407743 |
| pF vs aL | Cell wall/membrane/envelope biogenesis [M] | -0.561884553 | 0.689171 | 0.026776069 |
| pF vs aL | Energy production and conversion [C] | 0.890725625 | 1.033308 | 0.017607957 |
| pF vs aL | Inorganic ion transport and metabolism [P] | 1.448662732 | 1.475213 | 0.018000027 |
| pF vs aL | Intracellular trafficking, secretion, and vesicular transport [U] | 2.513185459 | 0.648497 | 0.005491313 |
| pF vs aL | Nucleotide transport and metabolism [F] | 2.569369838 | 0.420265 | 0.007166048 |
| pF vs aL | Posttranslational modification, protein turnover, chaperones [O] | -2.83811557 | 0.579907 | 0.019525866 |
| pF vs aL | Replication, recombination and repair [L] | 1.503745598 | 1.292228 | 0.060785876 |
| pF vs aL | Secondary metabolites biosynthesis, transport and catabolism [Q] | 2.727130298 | 0.726096 | 0.015120894 |
| pF vs aL | Signal transduction mechanisms [T] | -2.747069826 | 0.248435 | 0.005985313 |
| pF vs aL | Transcription [K] | 9.669921791 | 0.454592 | 0.008198622 |

**Table S6.** Functional annotation, log2 fold change values and relative abundance of differentially abundant COG genes selected in the study (related to Figure 4B). VRS, Villum Research Station; pF, permafrost; aL, active layer; SE, standard error; Rel.ab, relative abundance.

| **VRS** | **Functional_category** | **Log2FoldChange** | **SE** | **Rel.ab** |
| --- | --- | --- | --- | --- |
| pF vs aL | Amino acid transport and metabolism [E] | 2.841220929 | 0.271438567 | 0.029922 |
| pF vs aL | Cell wall/membrane/envelope biogenesis [M] | 2.82092824 | 0.256785514 | 0.032821 |
| pF vs aL | Coenzyme transport and metabolism [H] | 2.999109192 | 0.22540519 | 0.014327 |
| pF vs aL | Defense mechanisms [V] | 4.153256394 | 0.361681862 | 0.005579 |
| pF vs aL | Energy production and conversion [C] | 1.897643748 | 0.277388956 | 0.021704 |
| pF vs aL | General function prediction only [R] | 2.682488009 | 0.298746068 | 0.061203 |
| pF vs aL | Inorganic ion transport and metabolism [P] | 1.541932993 | 0.283014307 | 0.019918 |
| pF vs aL | Lipid transport and metabolism [I] | 4.923172536 | 0.41643509 | 0.045886 |
| pF vs aL | Nucleotide transport and metabolism [F] | 2.440841696 | 0.274676681 | 0.003601 |
| pF vs aL | Replication, recombination and repair [L] | 1.65143417 | 0.339715263 | 0.006385 |
| pF vs aL | Secondary metabolites biosynthesis, transport and catabolism [Q] | 2.016731647 | 0.3451427 | 0.012902 |
| pF vs aL | Signal transduction mechanisms [T] | 0.984168904 | 0.239727936 | 0.02423 |
| pF vs aL | Transcription [K] | -2.115166572 | 0.381130858 | 0.003024 |
| pF vs aL | Translation, ribosomal structure and biogenesis [J] | 3.425546449 | 0.373664408 | 0.010168 |

**Table S7.** Functional annotation, log2 fold change values and relative abundance of differentially abundant COG genes selected in the study (related to Figure 4C). aL, active layer; LAV, Val Lavriun; VRS, Villum Research Station; SE, standard error; Rel.ab, relative abundance.

| **aL** | **Functional_category** | **Log2FoldChange** | **SE** | **Rel.ab** |
| --- | --- | --- | --- | --- |
| LAV vs VRS | Amino acid transport and metabolism [E] | 1.739535107 | 0.555776903 | 0.013669845 |
| LAV vs VRS | Carbohydrate transport and metabolism [G] | 1.483236877 | 0.439364072 | 0.094797087 |
| LAV vs VRS | Cell cycle control, cell division, chromosome partitioning [D] | 3.124631914 | 0.362161765 | 0.021042197 |
| LAV vs VRS | Cell wall/membrane/envelope biogenesis [M] | 2.46236767 | 0.427937259 | 0.145379903 |
| LAV vs VRS | Coenzyme transport and metabolism [H] | 2.842875311 | 0.422890745 | 0.042463239 |
| LAV vs VRS | Defense mechanisms [V] | 0.094679592 | 0.370245863 | 0.02871049 |
| LAV vs VRS | Energy production and conversion [C] | 3.410930412 | 0.509825003 | 0.066444011 |
| LAV vs VRS | Inorganic ion transport and metabolism [P] | 1.023168597 | 0.423775189 | 0.098373985 |
| LAV vs VRS | Intracellular trafficking, secretion, and vesicular transport [U] | 2.544336658 | 0.375325078 | 0.079223075 |
| LAV vs VRS | Lipid transport and metabolism [I] | 3.360732858 | 0.452721161 | 0.026784893 |
| LAV vs VRS | Nucleotide transport and metabolism [F] | 3.499828895 | 0.445225732 | 0.086180635 |
| LAV vs VRS | Posttranslational modification, protein turnover, chaperones [O] | 4.28911946 | 0.467786139 | 0.101604518 |
| LAV vs VRS | Replication, recombination and repair [L] | 4.526953333 | 0.425196447 | 0.093155225 |
| LAV vs VRS | Secondary metabolites biosynthesis, transport and catabolism [Q] | -0.930315878 | 0.581623642 | 0.03652018 |
| LAV vs VRS | Signal transduction mechanisms [T] | 3.213472949 | 0.464096383 | 0.186961554 |
| LAV vs VRS | Transcription [K] | 3.387235053 | 0.448527501 | 0.032481717 |
| LAV vs VRS | Translation, ribosomal structure and biogenesis [J] | 3.110886535 | 0.436274176 | 0.006406476 |

**Table S8.** Functional annotation, log2 fold change values and relative abundance of differentially abundant COG genes selected in the study (related to Figure 4D). pF, permafrost; LAV, Val Lavriun; VRS, Villum Research Station; SE, standard error; Rel.ab, relative abundance.

| **pF** | **Functional_category** | **Log2FoldChange** | **SE** | **Rel.ab** |
| --- | --- | --- | --- | --- |
| LAV vs VRS | Amino acid transport and metabolism [E] | -0.341259369 | 0.188771766 | 0.178244764 |
| LAV vs VRS | Carbohydrate transport and metabolism [G] | -0.478230528 | 0.140900674 | 0.217184849 |
| LAV vs VRS | Cell Motility [N] | -2.900568296 | 0.119910741 | 0.009292936 |
| LAV vs VRS | Cell wall/membrane/envelope biogenesis [M] | 0.57920925 | 0.233383573 | 0.163029705 |
| LAV vs VRS | Coenzyme transport and metabolism [H] | -4.037470644 | 0.251278105 | 0.025786407 |
| LAV vs VRS | Defense mechanisms [V] | 2.770423389 | 0.117853803 | 0.09729187 |
| LAV vs VRS | Energy production and conversion [C] | -0.447179273 | 0.250534515 | 0.106423767 |
| LAV vs VRS | Inorganic ion transport and metabolism [P] | -0.878748479 | 0.182991612 | 0.245871201 |
| LAV vs VRS | Intracellular trafficking, secretion, and vesicular transport [U] | 2.583185166 | 0.150025782 | 0.117892939 |
| LAV vs VRS | Lipid transport and metabolism [I] | -2.654527512 | 0.168186013 | 0.115074325 |
| LAV vs VRS | Nucleotide transport and metabolism [F] | 1.390426496 | 0.165026705 | 0.040569026 |
| LAV vs VRS | Partially unkonwn function [S] | 0.085034295 | 0.16005536 | 0.758080343 |
| LAV vs VRS | Posttranslational modification, protein turnover, chaperones [O] | 1.12575392 | 0.175561914 | 0.147791602 |
| LAV vs VRS | Replication, recombination and repair [L] | 0.30745178 | 0.181043828 | 0.217565392 |
| LAV vs VRS | Secondary metabolites biosynthesis, transport and catabolism [Q] | -2.519224431 | 0.246855963 | 0.060813819 |
| LAV vs VRS | Signal transduction mechanisms [T] | 2.549266175 | 0.150519939 | 0.200918792 |
| LAV vs VRS | Transcription [K] | 2.132504232 | 0.170954058 | 0.07159476 |

**Table S9.** Functional annotation, log2 fold change values and relative abundance of differentially abundant COG genes selected in the study (related to Figure 5A). LAV, Val Lavirun; pF, permafrost; aL, active layer; Rel.ab, relative abundance.

| **LAV** | **COG** | **Functional_category** | **Log2FoldChange** | **Rel.ab** | **Eggnog_Function** |
| --- | --- | --- | --- | --- | --- |
| pF vs aL | COG0457 | Amino acid transport and metabolism [E] | 3.57426318 | 0.0088555 | Transglutaminase-like superfamily |
| pF vs aL | COG0460 | Amino acid transport and metabolism [E] | -4.011095438 | 0.009779607 | homoserine dehydrogenase |
| pF vs aL | COG1305 | Amino acid transport and metabolism [E] | -2.74960936 | 0.006139861 | domain protein |
| pF vs aL | COG3387 | Carbohydrate transport and metabolism [G] | -3.486983978 | 0.006315214 | glucan 1,4-alpha-glucosidase (EC 3.2.1.3) |
| pF vs aL | COG3640 | Cell cycle control, cell division, chromosome partitioning [D] | -2.710972409 | 0.02407743 | Cobyrinic acid ac-diamide synthase |
| pF vs aL | COG2335 | Cell wall/membrane/envelope biogenesis [M] | -3.716000712 | 0.011886294 | Fasciclin domain |
| pF vs aL | COG3209 | Cell wall/membrane/envelope biogenesis [M] | 2.592231606 | 0.014889775 | RHS Repeat |
| pF vs aL | COG0427 | Energy production and conversion [C] | -2.843202764 | 0.005489715 | 4-hydroxybutyrate coenzyme A transferase |
| pF vs aL | COG0437 | Energy production and conversion [C] | 2.807230275 | 0.006706626 | Cyclic nucleotide-binding domain |
| pF vs aL | COG2041 | Energy production and conversion [C] | 2.708149363 | 0.005411615 | Oxidoreductase, molybdopterin binding protein |
| pF vs aL | COG0038 | Inorganic ion transport and metabolism [P] | 3.219378813 | 0.007099783 | Voltage gated chloride channel |
| pF vs aL | COG0370 | Inorganic ion transport and metabolism [P] | -2.854513539 | 0.005078343 | Ferrous iron |
| pF vs aL | COG1118 | Inorganic ion transport and metabolism [P] | 3.981122923 | 0.005821901 | Molybdate ABC transporter |
| pF vs aL | COG0726 | Intracellular trafficking, secretion, and vesicular transport [U] | 2.513185459 | 0.005491313 | virulence factor family protein |
| pF vs aL | COG0213 | Nucleotide transport and metabolism [F] | 2.569369838 | 0.007166048 | pyrimidine-nucleoside phosphorylase |
| pF vs aL | COG0309 | Posttranslational modification, protein turnover, chaperones [O] | -2.562092184 | 0.010053866 | air synthase related protein domain protein;hydrogenase expression formation protein (HypE) |
| pF vs aL | COG0409 | Posttranslational modification, protein turnover, chaperones [O] | -3.114138955 | 0.009472 | hydrogenase expression formation protein HypD |
| pF vs aL | COG0749 | Replication, recombination and repair [L] | 3.050033747 | 0.005104309 | POLAc |
| pF vs aL | COG1518 | Replication, recombination and repair [L] | 2.594130766 | 0.012475288 | CRISPR (clustered regularly interspaced short palindromic repeat) |
| pF vs aL | COG3316 | Replication, recombination and repair [L] | -2.887988969 | 0.031513646 | Transposase |
| pF vs aL | COG4889 | Replication, recombination and repair [L] | 3.258806848 | 0.011692632 | helicase |
| pF vs aL | COG0827 | Secondary metabolites biosynthesis, transport and catabolism [Q] | 2.83932437 | 0.00766536 | Eco57I restriction-modification methylase |
| pF vs aL | COG2027 | Secondary metabolites biosynthesis, transport and catabolism [Q] | 2.614936225 | 0.007455533 | Deacylase |
| pF vs aL | COG4579 | Signal transduction mechanisms [T] | -2.747069826 | 0.005985313 | Bifunctional enzyme which can phosphorylate or dephosphorylate isocitrate  dehydrogenase (IDH) on a specific serine residue |
| pF vs aL | COG5108 | Transcription [K] | 9.669921791 | 0.008198622 | DNA-dependent RNA polymerase catalyzes the transcription of DNA into RNA using the four ribonucleoside triphosphates as substrates (By similarity) |

**Table S10.** Functional annotation, log2 fold change values and relative abundance of differentially abundant COG genes selected in the study (related to Figure 5B). VRS, Villum Research Station; pF, permafrost; aL, active layer; Rel.ab, relative abundance.

| **VRS** | **COG** | **Functional_category** | **Log2FoldChange** | **Rel.ab** | **Eggnog_Function** |
| --- | --- | --- | --- | --- | --- |
| pF vs aL | COG0506 | Amino acid transport and metabolism [E] | 2.055614795 | 0.021844346 | acetyl-CoA carboxylase, biotin carboxylase |
| pF vs aL | COG0765 | Amino acid transport and metabolism [E] | 4.645265981 | 0.001709801 | integral membrane protein |
| pF vs aL | COG1362 | Amino acid transport and metabolism [E] | 2.305178598 | 0.004796689 | DNA polymerase III (alpha subunit) |
| pF vs aL | COG4359 | Amino acid transport and metabolism [E] | 2.358824343 | 0.001570666 | Mur ligase middle domain |
| pF vs aL | COG0457 | Cell wall/membrane/envelope biogenesis [M] | 2.163470563 | 0.002314701 | Methyltransferase |
| pF vs aL | COG0489 | Cell wall/membrane/envelope biogenesis [M] | 2.050342783 | 0.01986333 | Responsible for the amidation of carboxylic groups at position A and C of either cobyrinic acid or hydrogenobrynic acid. |
| pF vs aL | COG0791 | Cell wall/membrane/envelope biogenesis [M] | 2.025643732 | 0.001429892 | precorrin-6x reductase |
| pF vs aL | COG1083 | Cell wall/membrane/envelope biogenesis [M] | 3.333776038 | 0.003630229 | GTP-binding protein |
| pF vs aL | COG1887 | Cell wall/membrane/envelope biogenesis [M] | 4.531408083 | 0.005583148 | Bacterial protein of unknown function (DUF881) |
| pF vs aL | COG0079 | Coenzyme transport and metabolism [H] | 3.580037673 | 0.009918243 | Antibiotic biosynthesis monooxygenase |
| pF vs aL | COG2099 | Coenzyme transport and metabolism [H] | 2.418180712 | 0.004408992 | high-affinity nickel-transporter |
| pF vs aL | COG3587 | Defense mechanisms [V] | 5.295265282 | 0.001319687 | Phage tail tape measure protein |
| pF vs aL | COG4988 | Defense mechanisms [V] | 3.011247507 | 0.004258859 | Germane |
| pF vs aL | COG0247 | Energy production and conversion [C] | 2.070944224 | 0.001882291 | carboxylase, carboxyl transferase |
| pF vs aL | COG0493 | Energy production and conversion [C] | 4.848569447 | 0.010478939 | YhgE Pip N-terminal domain protein |
| pF vs aL | COG1017 | Energy production and conversion [C] | -2.266987876 | 0.002649273 | Uncharacterised ACR, YkgG family COG1556 |
| pF vs aL | COG1018 | Energy production and conversion [C] | 2.51138994 | 0.004418684 | binding-protein-dependent transport systems inner membrane component |
| pF vs aL | COG1622 | Energy production and conversion [C] | 2.324303005 | 0.00227452 | nlp p60 protein |
| pF vs aL | COG1306 | General function prediction only [R] | 2.109121857 | 0.004664702 | Glycerol-3-phosphate cytidylyltransferase |
| pF vs aL | COG1511 | General function prediction only [R] | 3.840012754 | 0.003644133 | metal-dependent hydrolase |
| pF vs aL | COG1739 | General function prediction only [R] | 6.618486108 | 0.001757083 | signal transduction histidine kinase regulating citrate malate metabolism |
| pF vs aL | COG2153 | General function prediction only [R] | 2.291942517 | 0.002356454 | WD-40 repeat-containing protein |
| pF vs aL | COG2966 | General function prediction only [R] | 2.387673301 | 0.003863498 | Capsular exopolysaccharide family |
| pF vs aL | COG3224 | General function prediction only [R] | 2.161698579 | 0.002664712 | Specifically dimethylates two adjacent adenosines (A1518 and A1519) in the loop of a conserved hairpin near the 3'-end of 16S rRNA in the 30S particle. |
| pF vs aL | COG3687 | General function prediction only [R] | 2.173193726 | 0.005105648 | sigma 54 modulation protein ribosomal protein S30EA |
| pF vs aL | COG3818 | General function prediction only [R] | 5.10649301 | 0.002267415 | Membrane |
| pF vs aL | COG3879 | General function prediction only [R] | 2.284601036 | 0.012543986 | Penicillin amidase |
| pF vs aL | COG4425 | General function prediction only [R] | -2.034508398 | 0.011296686 | amino acid ABC transporter |
| pF vs aL | COG4872 | General function prediction only [R] | 4.651256351 | 0.002141865 | Amino acid export carrier protein |
| pF vs aL | COG4929 | General function prediction only [R] | 3.324197217 | 0.001602152 | Membrane |
| pF vs aL | COG5283 | General function prediction only [R] | 5.209496155 | 0.00149347 | Acetyltransferase (GNAT) family |
| pF vs aL | COG5401 | General function prediction only [R] | -2.265089717 | 0.001825595 | cytochrome c oxidase |
| pF vs aL | COG5522 | General function prediction only [R] | 2.378745642 | 0.003975371 | cytidylyltransferase |
| pF vs aL | COG1292 | Inorganic ion transport and metabolism [P] | 2.393417686 | 0.00598673 | protein-L-isoaspartate O-methyltransferase (EC 2.1.1.77) |
| pF vs aL | COG2375 | Inorganic ion transport and metabolism [P] | 2.790463877 | 0.008913305 | extracellular nuclease |
| pF vs aL | COG3376 | Inorganic ion transport and metabolism [P] | 2.01255189 | 0.0014348 | 2,3-diketo-5-methylthio-1-phosphopentane phosphatase |
| pF vs aL | COG3720 | Inorganic ion transport and metabolism [P] | -2.002909306 | 0.001964214 | Delta-1-pyrroline-5-carboxylate dehydrogenase |
| pF vs aL | COG4986 | Inorganic ion transport and metabolism [P] | 2.516140817 | 0.001618903 | Siderophore-interacting protein |
| pF vs aL | COG0304 | Lipid transport and metabolism [I] | 4.746866923 | 0.026961848 | Inherit from COG: Membrane |
| pF vs aL | COG0439 | Lipid transport and metabolism [I] | 2.315341746 | 0.006015455 | D12 class N6 adenine-specific DNA methyltransferase |
| pF vs aL | COG0615 | Lipid transport and metabolism [I] | 6.953942502 | 0.002816545 | transcriptional regulatory protein |
| pF vs aL | COG0777 | Lipid transport and metabolism [I] | 2.599412427 | 0.008034187 | non-ribosomal peptide synthetase |
| pF vs aL | COG3239 | Lipid transport and metabolism [I] | 8.000299079 | 0.002057704 | Type III restriction enzyme, res subunit |
| pF vs aL | COG0737 | Nucleotide transport and metabolism [F] | 2.440841696 | 0.003601123 | Choline carnitine betaine |
| pF vs aL | COG0389 | Replication, recombination and repair [L] | -2.315954197 | 0.002203184 | Globin |
| pF vs aL | COG3392 | Replication, recombination and repair [L] | 2.699637277 | 0.001329527 | IucA IucC family protein |
| pF vs aL | COG4335 | Replication, recombination and repair [L] | 4.570619428 | 0.00285272 | Gcn5-related n-acetyltransferase |
| pF vs aL | COG0147 | Secondary metabolites biosynthesis, transport and catabolism [Q] | -2.092984651 | 0.001624551 | Fatty acid desaturase |
| pF vs aL | COG1670 | Secondary metabolites biosynthesis, transport and catabolism [Q] | 5.661351528 | 0.005420545 | Impact family member yigz |
| pF vs aL | COG2312 | Secondary metabolites biosynthesis, transport and catabolism [Q] | 2.416941632 | 0.001800437 | hemin transport protein |
| pF vs aL | COG3135 | Secondary metabolites biosynthesis, transport and catabolism [Q] | 2.08161808 | 0.004056692 | fatty acid synthase |
| pF vs aL | COG1352 | Signal transduction mechanisms [T] | -2.048693647 | 0.004476191 | M18 family aminopeptidase |
| pF vs aL | COG3290 | Signal transduction mechanisms [T] | 2.383910194 | 0.013094626 | Teichoic acid biosynthesis protein |
| pF vs aL | COG4565 | Signal transduction mechanisms [T] | 2.617290165 | 0.006659056 | benzoate transporter |
| pF vs aL | COG1357 | Transcription [K] | -2.115166572 | 0.003023825 | metal cluster binding |
| pF vs aL | COG0030 | Translation, ribosomal structure and biogenesis [J] | -2.212468809 | 0.001743309 | nitric oxide dioxygenase (EC 1.14.12.17) |
| pF vs aL | COG1040 | Translation, ribosomal structure and biogenesis [J] | 6.50215371 | 0.00236714 | MCP methyltransferase methylesterase, CheR CheB with PAS PAC sensor |
| pF vs aL | COG2366 | Translation, ribosomal structure and biogenesis [J] | 7.096447483 | 0.00265981 | ABC transporter |
| pF vs aL | COG2813 | Translation, ribosomal structure and biogenesis [J] | 2.316053411 | 0.003397785 | DNA alkylation repair |

**Table S11.** Functional annotation, log2 fold change values and relative abundance of differentially abundant COG genes selected in the study (related to Figure 5C). aL, active layer; LAV, Val Lavriun; VRS, Villum Research Station; Rel.ab, relative abundance.

| **aL** | **COG** | **Functional_category** | **Log2FoldChange** | **Rel.ab** | **Eggnog_Function** |
| --- | --- | --- | --- | --- | --- |
| LAV vs VRS | COG1775 | Amino acid transport and metabolism [E] | 6.265019984 | 0.008467 | Benzoyl-CoA reductase |
| LAV vs VRS | COG4597 | Amino acid transport and metabolism [E] | -2.78594977 | 0.005202 | Polar amino acid ABC transporter, inner membrane subunit |
| LAV vs VRS | COG0469 | Carbohydrate transport and metabolism [G] | 3.169493966 | 0.014756 | Pyruvate kinase |
| LAV vs VRS | COG0524 | Carbohydrate transport and metabolism [G] | -2.512294557 | 0.006381 | PfkB domain-containing protein |
| LAV vs VRS | COG1449 | Carbohydrate transport and metabolism [G] | 4.563894577 | 0.007912 | pullulanase |
| LAV vs VRS | COG2730 | Carbohydrate transport and metabolism [G] | 2.878873624 | 0.023992 | CBM_3 |
| LAV vs VRS | COG3858 | Carbohydrate transport and metabolism [G] | 3.962346136 | 0.017459 | hydrolase family 18 |
| LAV vs VRS | COG4664 | Carbohydrate transport and metabolism [G] | -3.162892483 | 0.024298 | Trap dicarboxylate transporter, dctm subunit |
| LAV vs VRS | COG3640 | Cell cycle control, cell division, chromosome partitioning [D] | 3.124631914 | 0.021042 | Cobyrinic acid ac-diamide synthase |
| LAV vs VRS | COG0739 | Cell wall/membrane/envelope biogenesis [M] | 4.969502025 | 0.01251 | peptidase M23 |
| LAV vs VRS | COG1109 | Cell wall/membrane/envelope biogenesis [M] | 2.716877719 | 0.035831 | Mannose-1-phosphate guanyltransferase |
| LAV vs VRS | COG1388 | Cell wall/membrane/envelope biogenesis [M] | 6.718093078 | 0.008205 | CHAP domain |
| LAV vs VRS | COG2943 | Cell wall/membrane/envelope biogenesis [M] | -2.70168685 | 0.010889 | Involved in the biosynthesis of osmoregulated periplasmic glucans (OPGs) (By similarity) |
| LAV vs VRS | COG3209 | Cell wall/membrane/envelope biogenesis [M] | 4.39375034 | 0.005852 | YD repeat protein |
| LAV vs VRS | COG3511 | Cell wall/membrane/envelope biogenesis [M] | 3.850008713 | 0.05942 | Membrane-associated phospholipase C 2 |
| LAV vs VRS | COG3773 | Cell wall/membrane/envelope biogenesis [M] | -3.411162819 | 0.006409 | Cell wall hydrolase |
| LAV vs VRS | COG5498 | Cell wall/membrane/envelope biogenesis [M] | 3.163559152 | 0.006263 | Endo-1,3(4)-beta-glucanase (EC 3.2.1.6) |
| LAV vs VRS | COG0095 | Coenzyme transport and metabolism [H] | 2.93133592 | 0.014828 | biotin lipoate A B protein ligase;Lipoate-protein, ligase |
| LAV vs VRS | COG1239 | Coenzyme transport and metabolism [H] | 2.754414701 | 0.027635 | magnesium-chelatase |
| LAV vs VRS | COG2348 | Defense mechanisms [V] | 3.189630101 | 0.021166 | Methicillin resistance protein |
| LAV vs VRS | COG4618 | Defense mechanisms [V] | -3.000270916 | 0.007545 | Type I secretion system ATPase |
| LAV vs VRS | COG0247 | Energy production and conversion [C] | 4.932571032 | 0.02041 | Uncharacterised ACR, YkgG family COG1556 |
| LAV vs VRS | COG0280 | Energy production and conversion [C] | -3.080056425 | 0.005935 | Involved in acetate metabolism (By similarity) |
| LAV vs VRS | COG0511 | Energy production and conversion [C] | 3.351940834 | 0.023025 | pyruvate carboxylase subunit B |
| LAV vs VRS | COG3301 | Energy production and conversion [C] | 5.168088252 | 0.010252 | Polysulphide reductase, NrfD |
| LAV vs VRS | COG4658 | Energy production and conversion [C] | 6.682108369 | 0.006822 | Electron transport complex, RnfABCDGE type, D subunit |
| LAV vs VRS | COG0540 | Inorganic ion transport and metabolism [P] | 6.209933125 | 0.005014 | mce related protein |
| LAV vs VRS | COG0701 | Inorganic ion transport and metabolism [P] | 6.012476597 | 0.005951 | Predicted permease |
| LAV vs VRS | COG2358 | Inorganic ion transport and metabolism [P] | -2.583413029 | 0.018083 | TRAP transporter solute receptor TAXI family |
| LAV vs VRS | COG2372 | Inorganic ion transport and metabolism [P] | 4.943425328 | 0.014463 | transport integral membrane protein |
| LAV vs VRS | COG3131 | Inorganic ion transport and metabolism [P] | -2.712750651 | 0.010067 | Involved in the biosynthesis of osmoregulated periplasmic glucans (OPGs) (By similarity) |
| LAV vs VRS | COG3303 | Inorganic ion transport and metabolism [P] | -3.570660588 | 0.007878 | Plays a role in nitrite reduction (By similarity) |
| LAV vs VRS | COG3376 | Inorganic ion transport and metabolism [P] | 4.663295043 | 0.015258 | High-affinity nickel-transport protein |
| LAV vs VRS | COG4174 | Inorganic ion transport and metabolism [P] | -2.575806154 | 0.00581 | binding-protein-dependent transport systems inner membrane component |
| LAV vs VRS | COG4665 | Inorganic ion transport and metabolism [P] | -3.665349808 | 0.009194 | tripartite ATP-independent periplasmic transporter, DctQ component |
| LAV vs VRS | COG4986 | Inorganic ion transport and metabolism [P] | 3.510536104 | 0.006657 | inner membrane component |
| LAV vs VRS | COG3451 | Intracellular trafficking, secretion, and vesicular transport [U] | 2.502425986 | 0.062698 | traE protein |
| LAV vs VRS | COG3843 | Intracellular trafficking, secretion, and vesicular transport [U] | 2.555380854 | 0.00797 | Protein of unknown function (DUF3363) |
| LAV vs VRS | COG5158 | Intracellular trafficking, secretion, and vesicular transport [U] | 2.575203133 | 0.008555 | syntaxin binding protein |
| LAV vs VRS | COG0432 | Lipid transport and metabolism [I] | 3.180668361 | 0.006237 | Catalyzes the 1,3-allylic rearrangement of the homoallylic substrate isopentenyl (IPP) to its allylic isomer,  dimethylallyl diphosphate (DMAPP) (By similarity) |
| LAV vs VRS | COG1924 | Lipid transport and metabolism [I] | 4.119546749 | 0.007172 | CoA-substrate-specific enzyme activase |
| LAV vs VRS | COG3255 | Lipid transport and metabolism [I] | 3.301135522 | 0.008081 | sterol-binding domain protein |
| LAV vs VRS | COG3468 | Lipid transport and metabolism [I] | 2.841580801 | 0.005294 | outer membrane autotransporter barrel |
| LAV vs VRS | COG0037 | Nucleotide transport and metabolism [F] | 3.822493024 | 0.005376 | hypoxanthine phosphoribosyltransferase |
| LAV vs VRS | COG0283 | Nucleotide transport and metabolism [F] | 4.347682709 | 0.005164 | bifunctional pantoate ligase cytidylate kinase |
| LAV vs VRS | COG1001 | Nucleotide transport and metabolism [F] | 2.965801641 | 0.017964 | adenine deaminase |
| LAV vs VRS | COG1457 | Nucleotide transport and metabolism [F] | 2.930197981 | 0.038129 | permease for cytosine purines, uracil, thiamine, allantoin |
| LAV vs VRS | COG2131 | Nucleotide transport and metabolism [F] | 3.778631497 | 0.008499 | CMP dCMP deaminase zinc-binding;deaminase |
| LAV vs VRS | COG2820 | Nucleotide transport and metabolism [F] | 3.154166518 | 0.011049 | Uridine phosphorylase |
| LAV vs VRS | COG0109 | Posttranslational modification, protein turnover, chaperones [O] | 3.740208154 | 0.005629 | Converts heme B (protoheme IX) to heme O by substitution of the vinyl group on carbon 2 of heme B porphyrin ring  with a hydroxyethyl farnesyl side group (By similarity) |
| LAV vs VRS | COG1026 | Posttranslational modification, protein turnover, chaperones [O] | 2.938264214 | 0.020678 | peptidase |
| LAV vs VRS | COG4870 | Posttranslational modification, protein turnover, chaperones [O] | 2.837724812 | 0.021903 | peptidase C1A, papain |
| LAV vs VRS | COG4934 | Posttranslational modification, protein turnover, chaperones [O] | 4.469658916 | 0.047413 | Pro-kumamolisin, activation domain |
| LAV vs VRS | COG5504 | Posttranslational modification, protein turnover, chaperones [O] | 7.459741205 | 0.005981 | Predicted Zn-dependent protease (DUF2268) |
| LAV vs VRS | COG0515 | Replication, recombination and repair [L] | 7.296633037 | 0.016944 | Inherit from COG: Serine Threonine protein kinase |
| LAV vs VRS | COG0847 | Replication, recombination and repair [L] | 5.095178857 | 0.020125 | DNA polymerase III, epsilon subunit;Bifunctional ATP-dependent DNA helicase DNA polymerase III subunit epsilon |
| LAV vs VRS | COG1199 | Replication, recombination and repair [L] | 4.829508983 | 0.0138 | Helicase |
| LAV vs VRS | COG1236 | Replication, recombination and repair [L] | 2.700764902 | 0.026405 | DNA ligase N terminus |
| LAV vs VRS | COG3359 | Replication, recombination and repair [L] | 2.712680885 | 0.01588 | Inherit from COG: Exonuclease-like protein;Exonuclease-like protein;Inherit from bactNOG: Exonuclease-like protein;  Inherit from chloNOG: exonuclease-like protein |
| LAV vs VRS | COG3885 | Secondary metabolites biosynthesis, transport and catabolism [Q] | 4.339685408 | 0.008495 | dioxygenase |
| LAV vs VRS | COG4264 | Secondary metabolites biosynthesis, transport and catabolism [Q] | -3.664499315 | 0.008096 | IucA IucC family protein;iron transport protein |
| LAV vs VRS | COG4663 | Secondary metabolites biosynthesis, transport and catabolism [Q] | -3.466133726 | 0.019929 | extracellular solute-binding protein, family 7 |
| LAV vs VRS | COG0642 | Signal transduction mechanisms [T] | 2.765488039 | 0.007413 | Signal transduction histidine kinase |
| LAV vs VRS | COG3322 | Signal transduction mechanisms [T] | 6.467989475 | 0.005307 | Histidine kinase |
| LAV vs VRS | COG2114 | Signal transduction mechanisms [T] | 3.333114683 | 0.012361 | Adenylate Guanylate cyclase |
| LAV vs VRS | COG3903 | Signal transduction mechanisms [T] | 2.946195012 | 0.063221 | CYCc |
| LAV vs VRS | COG4252 | Signal transduction mechanisms [T] | 3.162513175 | 0.030778 | Adenylate and Guanylate cyclase catalytic domain |
| LAV vs VRS | COG2199 | Signal transduction mechanisms [T] | 2.880024863 | 0.02171 | Diguanylate cyclase and metal dependent phosphohydrolase |
| LAV vs VRS | COG2204 | Signal transduction mechanisms [T] | 3.477764842 | 0.006548 | HDc |
| LAV vs VRS | COG2206 | Signal transduction mechanisms [T] | 2.967626509 | 0.00966 | Transcriptional regulator |
| LAV vs VRS | COG3292 | Signal transduction mechanisms [T] | -2.955890254 | 0.010925 | GAF domain;Histidine kinase |
| LAV vs VRS | COG3851 | Signal transduction mechanisms [T] | 3.768244019 | 0.013624 | integral membrane sensor signal transduction histidine kinase |
| LAV vs VRS | COG4990 | Signal transduction mechanisms [T] | 6.535132082 | 0.005414 | Inherit from COG: domain protein |
| LAV vs VRS | COG0607 | Transcription [K] | 2.928911004 | 0.01108 | Transcriptional regulator |
| LAV vs VRS | COG3629 | Transcription [K] | 2.853512452 | 0.013727 | LuxR family transcriptional regulator |
| LAV vs VRS | COG2197 | Transcription [K] | 4.379281704 | 0.007675 | response regulator receiver protein |
| LAV vs VRS | COG2813 | Translation, ribosomal structure and biogenesis [J] | 3.110886535 | 0.006406 | Methyltransferase |

**Table S12.** Functional annotation, log2 fold change values and relative abundance of differentially abundant COG genes selected in the study (related to Figure 5D). pF, permafrost; LAV, Val Lavriun; VRS, Villum Research Station; Rel.ab, relative abundance.

| **pF** | **COG** | **Functional_category** | **Log2FoldChange** | **Rel.ab** | **Eggnog_Function** |
| --- | --- | --- | --- | --- | --- |
| LAV vs VRS | COG0147 | Amino acid transport and metabolism [E] | -5.011547504 | 0.016891 | anthranilate synthase component i |
| LAV vs VRS | COG0410 | Amino acid transport and metabolism [E] | 6.346991827 | 0.011172 | ABC transporter |
| LAV vs VRS | COG0457 | Amino acid transport and metabolism [E] | 6.297888317 | 0.00964 | Transglutaminase-like superfamily |
| LAV vs VRS | COG0506 | Amino acid transport and metabolism [E] | -4.513495603 | 0.008959 | bifunctional proline dehydrogenase pyrroline-5-carboxylate dehydrogenase |
| LAV vs VRS | COG0560 | Amino acid transport and metabolism [E] | -7.413942691 | 0.005492 | phosphoserine phosphatase |
| LAV vs VRS | COG0687 | Amino acid transport and metabolism [E] | -2.624092242 | 0.028032 | extracellular solute-binding protein, family 1 |
| LAV vs VRS | COG1305 | Amino acid transport and metabolism [E] | -4.537299747 | 0.015077 | transglutaminase;domain protein;Putative amidoligase enzyme (DUF2126) |
| LAV vs VRS | COG1707 | Amino acid transport and metabolism [E] | 2.521993434 | 0.011126 | Inherit from COG: Amino acid-binding act domain protein |
| LAV vs VRS | COG1775 | Amino acid transport and metabolism [E] | 5.530229745 | 0.01039 | Benzoyl-CoA reductase, subunit B |
| LAV vs VRS | COG2040 | Amino acid transport and metabolism [E] | -2.903530601 | 0.006306 | homocysteine S-methyltransferase |
| LAV vs VRS | COG2939 | Amino acid transport and metabolism [E] | 3.375563697 | 0.020045 | peptidase S10, serine carboxypeptidase;peptidase S10 serine carboxypeptidase |
| LAV vs VRS | COG3075 | Amino acid transport and metabolism [E] | 3.380087719 | 0.013192 | anaerobic glycerol-3-phosphate dehydrogenase, subunit B |
| LAV vs VRS | COG3200 | Amino acid transport and metabolism [E] | -2.56250766 | 0.014173 | phospho-2-dehydro-3-deoxyheptonate aldolase |
| LAV vs VRS | COG4577 | Amino acid transport and metabolism [E] | -2.663969853 | 0.00775 | Microcompartments protein |
| LAV vs VRS | COG0383 | Carbohydrate transport and metabolism [G] | 2.967262474 | 0.060434 | mannosidase, alpha, class 2C, member 1 |
| LAV vs VRS | COG1449 | Carbohydrate transport and metabolism [G] | 2.644449578 | 0.066643 | Glycoside hydrolase, family 57 |
| LAV vs VRS | COG4945 | Carbohydrate transport and metabolism [G] | 4.170181803 | 0.008542 | pullulanase |
| LAV vs VRS | COG2115 | Carbohydrate transport and metabolism [G] | -3.081559512 | 0.010764 | xylose isomerase |
| LAV vs VRS | COG2730 | Carbohydrate transport and metabolism [G] | 2.74992102 | 0.014651 | CBM_3;Glycoside hydrolase Family 5 |
| LAV vs VRS | COG2893 | Carbohydrate transport and metabolism [G] | -2.719338647 | 0.005371 | PTS System |
| LAV vs VRS | COG2942 | Carbohydrate transport and metabolism [G] | -2.879674006 | 0.00524 | n-acylglucosamine 2-epimerase |
| LAV vs VRS | COG3001 | Carbohydrate transport and metabolism [G] | -2.561231802 | 0.005388 | Fructosamine kinase |
| LAV vs VRS | COG3537 | Carbohydrate transport and metabolism [G] | -2.769964079 | 0.005226 | Glycosyl hydrolase family 92 |
| LAV vs VRS | COG3858 | Carbohydrate transport and metabolism [G] | 2.845876269 | 0.012453 | hydrolase family 18 |
| LAV vs VRS | COG4284 | Carbohydrate transport and metabolism [G] | -3.393330489 | 0.007697 | UTP-glucose-1-phosphate uridylyltransferase |
| LAV vs VRS | COG4664 | Carbohydrate transport and metabolism [G] | -3.711358952 | 0.014775 | Trap dicarboxylate transporter, dctm subunit |
| LAV vs VRS | COG5008 | Cell Motility [N] | -2.900568296 | 0.009293 | twitching motility protein |
| LAV vs VRS | COG1215 | Cell wall/membrane/envelope biogenesis [M] | 2.948244588 | 0.016351 | Glycosyl transferase, family 2 |
| LAV vs VRS | COG0739 | Cell wall/membrane/envelope biogenesis [M] | 3.255780637 | 0.009893 | peptidase M23 |
| LAV vs VRS | COG1388 | Cell wall/membrane/envelope biogenesis [M] | 5.989670541 | 0.010814 | CHAP domain |
| LAV vs VRS | COG2335 | Cell wall/membrane/envelope biogenesis [M] | -3.672939496 | 0.0105 | Beta-Ig-H3 fasciclin |
| LAV vs VRS | COG2943 | Cell wall/membrane/envelope biogenesis [M] | -6.094925944 | 0.005263 | Involved in the biosynthesis of osmoregulated periplasmic glucans (OPGs) (By similarity) |
| LAV vs VRS | COG2951 | Cell wall/membrane/envelope biogenesis [M] | -3.78749611 | 0.026689 | Lytic murein transglycosylase B |
| LAV vs VRS | COG3178 | Cell wall/membrane/envelope biogenesis [M] | -2.572217399 | 0.007762 | Aminoglycoside phosphotransferase |
| LAV vs VRS | COG3209 | Cell wall/membrane/envelope biogenesis [M] | 5.775799841 | 0.01462 | RHS Repeat |
| LAV vs VRS | COG3511 | Cell wall/membrane/envelope biogenesis [M] | 3.370966588 | 0.061137 | Membrane-associated phospholipase C 2 |
| LAV vs VRS | COG0079 | Coenzyme transport and metabolism [H] | -7.993484039 | 0.009701 | Responsible for the amidation of carboxylic groups at position A and C  of either cobyrinic acid or hydrogenobrynic acid |
| LAV vs VRS | COG0303 | Coenzyme transport and metabolism [H] | 2.540358083 | 0.009195 | Molybdopterin |
| LAV vs VRS | COG1010 | Coenzyme transport and metabolism [H] | -6.659285977 | 0.00689 | precorrin-3B C17-methyltransferase |
| LAV vs VRS | COG0732 | Defense mechanisms [V] | 2.744623918 | 0.022233 | specificity;Type I restriction modification DNA specificity domain |
| LAV vs VRS | COG0827 | Defense mechanisms [V] | 2.91161252 | 0.008837 | methylase |
| LAV vs VRS | COG1002 | Defense mechanisms [V] | 2.65503373 | 0.066222 | Inherit from proNOG: methylase |
| LAV vs VRS | COG0247 | Energy production and conversion [C] | 4.095718232 | 0.026684 | Uncharacterised ACR, YkgG family COG1556 |
| LAV vs VRS | COG0427 | Energy production and conversion [C] | -3.271347396 | 0.006689 | acetyl-CoA hydrolase |
| LAV vs VRS | COG0437 | Energy production and conversion [C] | 3.104157331 | 0.007576 | Cyclic nucleotide-binding domain |
| LAV vs VRS | COG0493 | Energy production and conversion [C] | -7.362192709 | 0.010818 | Ferredoxin |
| LAV vs VRS | COG1034 | Energy production and conversion [C] | -2.787431081 | 0.006724 | subunit G;Oxidoreductase |
| LAV vs VRS | COG1858 | Energy production and conversion [C] | -3.659556306 | 0.018619 | Cytochrome c;SCO1 SenC family protein methylamine utilization protein |
| LAV vs VRS | COG2041 | Energy production and conversion [C] | 6.371166352 | 0.005323 | Oxidoreductase, molybdopterin binding protein |
| LAV vs VRS | COG2838 | Energy production and conversion [C] | -4.903909575 | 0.013741 | Isocitrate dehydrogenase |
| LAV vs VRS | COG3301 | Energy production and conversion [C] | 4.388781693 | 0.010251 | Polysulphide reductase, NrfD |
| LAV vs VRS | COG0038 | Inorganic ion transport and metabolism [P] | 2.751594485 | 0.016863 | chloride channel |
| LAV vs VRS | COG0517 | Inorganic ion transport and metabolism [P] | 5.736357706 | 0.007567 | Voltage gated chloride channel |
| LAV vs VRS | COG0529 | Inorganic ion transport and metabolism [P] | -5.177203265 | 0.010854 | May be the GTPase, regulating ATP sulfurylase activity (By similarity) |
| LAV vs VRS | COG0540 | Inorganic ion transport and metabolism [P] | 6.959489845 | 0.008233 | mce related protein |
| LAV vs VRS | COG0798 | Inorganic ion transport and metabolism [P] | 2.787447445 | 0.005932 | arsenical-resistance protein |
| LAV vs VRS | COG1118 | Inorganic ion transport and metabolism [P] | 6.699225149 | 0.006621 | Molybdate ABC transporter |
| LAV vs VRS | COG1613 | Inorganic ion transport and metabolism [P] | -5.779842857 | 0.012555 | sulfate ABC transporter |
| LAV vs VRS | COG2060 | Inorganic ion transport and metabolism [P] | -2.706697221 | 0.017091 | One of the components of the high-affinity ATP-driven potassium transport (or KDP) system, which catalyzes the hydrolysis of ATP coupled with the exchange of hydrogen and potassium ions (By similarity) |
| LAV vs VRS | COG2216 | Inorganic ion transport and metabolism [P] | -2.793318539 | 0.027266 | One of the components of the high-affinity ATP-driven potassium transport (or KDP) system, which catalyzes the hydrolysis of ATP coupled with the exchange of hydrogen and potassium ions (By similarity) |
| LAV vs VRS | COG2358 | Inorganic ion transport and metabolism [P] | -2.593960443 | 0.013076 | TRAP transporter solute receptor TAXI family |
| LAV vs VRS | COG2372 | Inorganic ion transport and metabolism [P] | 3.906629663 | 0.013436 | transport integral membrane protein |
| LAV vs VRS | COG2375 | Inorganic ion transport and metabolism [P] | -2.843855717 | 0.009045 | siderophore-interacting |
| LAV vs VRS | COG3004 | Inorganic ion transport and metabolism [P] | -2.553500045 | 0.013273 | Na( ) H( ) antiporter that extrudes sodium in exchange for external protons (By similarity) |
| LAV vs VRS | COG3131 | Inorganic ion transport and metabolism [P] | -5.477885832 | 0.005072 | Involved in the biosynthesis of osmoregulated periplasmic glucans (OPGs) (By similarity) |
| LAV vs VRS | COG3263 | Inorganic ion transport and metabolism [P] | -4.998403598 | 0.013535 | Sodium/hydrogen exchanger family |
| LAV vs VRS | COG3303 | Inorganic ion transport and metabolism [P] | -8.127749264 | 0.005354 | Plays a role in nitrite reduction (By similarity) |
| LAV vs VRS | COG3376 | Inorganic ion transport and metabolism [P] | 3.324430722 | 0.014297 | High-affinity;High-affinity nickel-transport protein |
| LAV vs VRS | COG3540 | Inorganic ion transport and metabolism [P] | -6.312077583 | 0.017635 | alkaline phosphatase |
| LAV vs VRS | COG3559 | Inorganic ion transport and metabolism [P] | 3.961623033 | 0.007322 | abc transporter |
| LAV vs VRS | COG4208 | Inorganic ion transport and metabolism [P] | -5.298080641 | 0.009601 | sulfate ABC transporter |
| LAV vs VRS | COG4393 | Inorganic ion transport and metabolism [P] | 3.484079876 | 0.005628 | Iron permease FTR1 |
| LAV vs VRS | COG4665 | Inorganic ion transport and metabolism [P] | -4.280769467 | 0.005615 | tripartite ATP-independent periplasmic transporter, DctQ component |
| LAV vs VRS | COG0341 | Intracellular trafficking, secretion, and vesicular transport [U] | -3.468882196 | 0.014742 | Protein-export membrane protein SecD |
| LAV vs VRS | COG0726 | Intracellular trafficking, secretion, and vesicular transport [U] | 5.667525218 | 0.005637 | virulence factor family protein |
| LAV vs VRS | COG3451 | Intracellular trafficking, secretion, and vesicular transport [U] | 3.210610525 | 0.060906 | traE protein;conjugal transfer ATPase |
| LAV vs VRS | COG3843 | Intracellular trafficking, secretion, and vesicular transport [U] | 2.93595629 | 0.007353 | Protein of unknown function (DUF3363);Inherit from proNOG: DNA relaxase nickase, TraS VirD2-like |
| LAV vs VRS | COG5295 | Intracellular trafficking, secretion, and vesicular transport [U] | 4.570715994 | 0.029255 | Pfam:HIM |
| LAV vs VRS | COG0304 | Lipid transport and metabolism [I] | -8.567590913 | 0.018021 | fatty acid synthase |
| LAV vs VRS | COG1703 | Lipid transport and metabolism [I] | -5.782590898 | 0.014798 | Methylmalonyl-CoA mutase |
| LAV vs VRS | COG1884 | Lipid transport and metabolism [I] | -4.491321172 | 0.033086 | Methylmalonyl-CoA mutase |
| LAV vs VRS | COG1924 | Lipid transport and metabolism [I] | 3.619839949 | 0.008281 | CoA-substrate-specific enzyme activase |
| LAV vs VRS | COG3243 | Lipid transport and metabolism [I] | -2.68063181 | 0.027776 | Poly-beta-hydroxybutyrate polymerase domain protein |
| LAV vs VRS | COG3255 | Lipid transport and metabolism [I] | 2.604687202 | 0.008029 | sterol-binding domain protein |
| LAV vs VRS | COG4553 | Lipid transport and metabolism [I] | -3.284084944 | 0.005082 | Polyhydroxyalkanoate depolymerase, intracellular |
| LAV vs VRS | COG0037 | Nucleotide transport and metabolism [F] | 3.734823777 | 0.006513 | hypoxanthine phosphoribosyltransferase |
| LAV vs VRS | COG0213 | Nucleotide transport and metabolism [F] | 3.671110152 | 0.010848 | pyrimidine-nucleoside phosphorylase |
| LAV vs VRS | COG2131 | Nucleotide transport and metabolism [F] | 3.041472385 | 0.006919 | CMP dCMP deaminase zinc-binding;deaminase |
| LAV vs VRS | COG2233 | Nucleotide transport and metabolism [F] | 2.632538561 | 0.010311 | permease |
| LAV vs VRS | COG4631 | Nucleotide transport and metabolism [F] | -6.127812397 | 0.005979 | Xanthine dehydrogenase |
| LAV vs VRS | COG0109 | Posttranslational modification, protein turnover, chaperones [O] | 2.985124119 | 0.006832 | Converts heme B (protoheme IX) to heme O by substitution of the vinyl group on carbon 2 of heme B porphyrin ring with a hydroxyethyl farnesyl side group (By similarity) |
| LAV vs VRS | COG0309 | Posttranslational modification, protein turnover, chaperones [O] | -2.594177156 | 0.009102 | hydrogenase expression formation protein HypE |
| LAV vs VRS | COG0409 | Posttranslational modification, protein turnover, chaperones [O] | -3.218184216 | 0.009135 | hydrogenase expression formation protein HypD |
| LAV vs VRS | COG1026 | Posttranslational modification, protein turnover, chaperones [O] | 3.087885892 | 0.029016 | peptidase |
| LAV vs VRS | COG1067 | Posttranslational modification, protein turnover, chaperones [O] | 3.930618333 | 0.011392 | Peptidase |
| LAV vs VRS | COG2127 | Posttranslational modification, protein turnover, chaperones [O] | -2.985349635 | 0.008168 | Involved in the modulation of the specificity of the ClpAP-mediated ATP-dependent protein degradation (By similarity) |
| LAV vs VRS | COG2360 | Posttranslational modification, protein turnover, chaperones [O] | -2.905034261 | 0.009251 | Functions in the N-end rule pathway of protein degradation where it conjugates Leu, Phe and, less efficiently, Met from aminoacyl-tRNAs to the N-termini of proteins containing an N-terminal arginine or lysine (By similarity) |
| LAV vs VRS | COG3058 | Posttranslational modification, protein turnover, chaperones [O] | 2.592685329 | 0.014546 | Necessary for formate dehydrogenase activity (By similarity) |
| LAV vs VRS | COG4934 | Posttranslational modification, protein turnover, chaperones [O] | 4.390766796 | 0.043119 | Pro-kumamolisin, activation domain |
| LAV vs VRS | COG5504 | Posttranslational modification, protein turnover, chaperones [O] | 5.973204002 | 0.007232 | Predicted Zn-dependent protease (DUF2268) |
| LAV vs VRS | COG0188 | Replication, recombination and repair [L] | 2.583585879 | 0.012358 | DNA gyrase negatively supercoils closed circular double- stranded DNA in an ATP-dependent manner and also catalyzes the interconversion of other topological isomers of double-stranded DNA rings, including catenanes and knotted rings (By similarity) |
| LAV vs VRS | COG0210 | Replication, recombination and repair [L] | -3.720617709 | 0.009276 | UvrD REP helicase |
| LAV vs VRS | COG0305 | Replication, recombination and repair [L] | 3.22439519 | 0.018214 | replicative DNA helicase |
| LAV vs VRS | COG0322 | Replication, recombination and repair [L] | -3.806418248 | 0.012904 | DNA polymerase III, epsilon subunit |
| LAV vs VRS | COG0515 | Replication, recombination and repair [L] | 5.648217808 | 0.010964 | Inherit from COG: Serine Threonine protein kinase |
| LAV vs VRS | COG0550 | Replication, recombination and repair [L] | -3.689991921 | 0.010709 | DNA topoisomerase iii |
| LAV vs VRS | COG0847 | Replication, recombination and repair [L] | 4.596927907 | 0.02439 | DNA polymerase III, epsilon subunit |
| LAV vs VRS | COG1112 | Replication, recombination and repair [L] | 3.795477904 | 0.041238 | DNA helicase |
| LAV vs VRS | COG1199 | Replication, recombination and repair [L] | 4.600620688 | 0.012866 | Helicase |
| LAV vs VRS | COG1330 | Replication, recombination and repair [L] | -3.7842568 | 0.005887 | exodeoxyribonuclease V, gamma |
| LAV vs VRS | COG1518 | Replication, recombination and repair [L] | 4.053887428 | 0.012862 | CRISPR (clustered regularly interspaced short palindromic repeat) |
| LAV vs VRS | COG1623 | Replication, recombination and repair [L] | -2.98376679 | 0.010894 | Participates in a DNA-damage check-point. DisA forms globular foci that rapidly scan along the chromosomes searching for lesions (By similarity) |
| LAV vs VRS | COG1637 | Replication, recombination and repair [L] | -2.79279031 | 0.007267 | Cleaves both 3' and 5' ssDNA extremities of branched DNA structures (By similarity) |
| LAV vs VRS | COG2887 | Replication, recombination and repair [L] | -2.932688687 | 0.005719 | recb family;Protein of unknown function (DUF2800) |
| LAV vs VRS | COG3145 | Replication, recombination and repair [L] | -2.507907912 | 0.007868 | 2og-fe(ii) oxygenase |
| LAV vs VRS | COG4889 | Replication, recombination and repair [L] | 2.634554052 | 0.014151 | helicase |
| LAV vs VRS | COG1670 | Secondary metabolites biosynthesis, transport and catabolism [Q] | -10.30376961 | 0.006002 | IucA IucC family protein |
| LAV vs VRS | COG2027 | Secondary metabolites biosynthesis, transport and catabolism [Q] | 3.487506646 | 0.007695 | Deacylase |
| LAV vs VRS | COG2175 | Secondary metabolites biosynthesis, transport and catabolism [Q] | -2.696395304 | 0.014223 | alpha-ketoglutarate-dependent 2,4-dichlorophenoxyacetate dioxygenase |
| LAV vs VRS | COG2357 | Secondary metabolites biosynthesis, transport and catabolism [Q] | -3.723841981 | 0.005014 | RelA SpoT domain protein;relA SpoT domain protein |
| LAV vs VRS | COG3885 | Secondary metabolites biosynthesis, transport and catabolism [Q] | 3.981810405 | 0.011608 | dioxygenase |
| LAV vs VRS | COG4569 | Secondary metabolites biosynthesis, transport and catabolism [Q] | -3.969854712 | 0.005163 | Catalyzes the conversion of acetaldehyde to acetyl-CoA, using NAD( ) and coenzyme A |
| LAV vs VRS | COG4663 | Secondary metabolites biosynthesis, transport and catabolism [Q] | -4.410026456 | 0.01111 | extracellular solute-binding protein, family 7 |
| LAV vs VRS | COG0642 | Signal transduction mechanisms [T] | 5.153812683 | 0.005099 | Histidine kinase;PAS PAC sensor signal transduction histidine kinase |
| LAV vs VRS | COG2203 | Signal transduction mechanisms [T] | 3.515594286 | 0.014065 | Signal transduction histidine kinase;Histidine kinase;multi-sensor signal transduction histidine kinase;GAF;Sensor Signal Transduction Histidine Kinase;Signal Transduction Histidine Kinase;Protein kinase domain;histidine Kinase |
| LAV vs VRS | COG2114 | Signal transduction mechanisms [T] | 3.281550717 | 0.039044 | Adenylate and Guanylate cyclase catalytic domain;Adenylate guanylate cyclase with Chase sensor;Adenylate guanylate cyclase;Adenylate guanylate Cyclase;Adenylate;Adenylate cyclase;CYCc;Adenylate Guanylate cyclase;Guanylate Cyclase;CHASE2 domain |
| LAV vs VRS | COG3903 | Signal transduction mechanisms [T] | 2.618815766 | 0.064355 | CYCc;Transcriptional regulator;Adenylate guanylate cyclase;transcriptional regulator;LuxR family transcriptional regulator;Transcriptional regulator, luxr family;Inherit from proNOG: transcriptional regulator |
| LAV vs VRS | COG2206 | Signal transduction mechanisms [T] | 3.869234147 | 0.010836 | HDc;Diguanylate cyclase and metal dependent phosphohydrolase;Metal dependent phosphohydrolase |
| LAV vs VRS | COG2204 | Signal transduction mechanisms [T] | 2.66266623 | 0.005134 | Stage II sporulation protein E (SpoIIE);protein serine threonine phosphatase |
| LAV vs VRS | COG2766 | Signal transduction mechanisms [T] | -2.72685504 | 0.0095 | Serine protein kinase;serine protein kinase PrkA |
| LAV vs VRS | COG3292 | Signal transduction mechanisms [T] | -2.857590958 | 0.005097 | GAF domain;Histidine kinase;Hybrid sensor;Y_Y_Y domain |
| LAV vs VRS | COG3851 | Signal transduction mechanisms [T] | 3.949783253 | 0.029555 | integral membrane sensor signal transduction histidine kinase;signal transduction Histidine kinase;Histidine kinase |
| LAV vs VRS | COG4191 | Signal transduction mechanisms [T] | 2.802298682 | 0.01093 | histidine Kinase;PAS PAC sensor signal transduction histidine kinase;Histidine kinase;sensory box histidine kinase response regulator;c4-dicarboxylate transport sensor protein;Signal Transduction Histidine Kinase;His Kinase A (phospho-acceptor) domain;signal transduction histidine kinase |
| LAV vs VRS | COG4990 | Signal transduction mechanisms [T] | 5.772618155 | 0.007304 | Inherit from COG: domain protein;;Inherit from COG: choline binding protein;Inherit from COG: Cell wall binding repeat 2-containing protein |
| LAV vs VRS | COG1678 | Transcription [K] | -2.952977441 | 0.008759 | UPF0301 protein;Uncharacterized ACR, COG1678 |
| LAV vs VRS | COG3629 | Transcription [K] | 2.830160461 | 0.016503 | LuxR family transcriptional regulator;CYCc |
| LAV vs VRS | COG2197 | Transcription [K] | 2.673725542 | 0.005867 | response regulator receiver protein |
| LAV vs VRS | COG3682 | Transcription [K] | 2.587559781 | 0.006844 | Pfam:Pencillinase_R |
| LAV vs VRS | COG4977 | Transcription [K] | -2.931961057 | 0.012183 | AraC family transcriptional regulator |
| LAV vs VRS | COG5048 | Transcription [K] | 3.484904395 | 0.00503 | Inherit from KOG: Zinc finger protein |
| LAV vs VRS | COG5108 | Transcription [K] | 7.814870436 | 0.01025 | RNA Polymerase |
| LAV vs VRS | COG5340 | Transcription [K] | 3.553751737 | 0.006158 | Inherit from bactNOG: transcriptional regulator-like protein |

**Table S13.** Functional annotation, log2 fold change values and relative abundance of differentially abundant CAZy genes selected in the study (related to Figure 6A). LAV, Val Lavirun; pF, permafrost; aL, active layer; SE, standard error; Rel.ab, relative abundance.

| **LAV** | **CAZy** | **Log2FoldChange** | **SE** | **Rel.ab** | **Category** | **Function** | **Group** |
| --- | --- | --- | --- | --- | --- | --- | --- |
| pF vs aL | AA3 | 5.716018113 | 0.880432636 | 0.000971016 | AuxiliaryActivities | oxidoreductases | Lignin |
| pF vs aL | AA7 | 6.595697591 | 0.762163692 | 0.000897994 | AuxiliaryActivities | glucooligosaccharide oxidase | Lignin |
| pF vs aL | CBM2 | 3.792927412 | 0.480324025 | 0.000639827 | Carbohydrate-BindingModules | cellulose-binding function | Cellulose |
| pF vs aL | CBM13 | -3.526829107 | 0.961959862 | 0.000752379 | Carbohydrate-BindingModules | glycoside hydrolases | Others |
| pF vs aL | CBM48 | -3.335516391 | 0.942319239 | 0.000665085 | Carbohydrate-BindingModules | glycogen-binding function | Starch/oligosaccharides |
| pF vs aL | CBM50 | 6.359220963 | 0.67641869 | 0.00098029 | Carbohydrate-BindingModules | chitin or peptidoglycan cleaving | Chitin |
| pF vs aL | CBM57 | 6.620263899 | 0.747070517 | 0.000620453 | Carbohydrate-BindingModules | glycosidases | Others |
| pF vs aL | CE4 | 5.201716364 | 1.05655226 | 0.001088042 | CarbohydrateEsterases | acetyl xylan esterase | Hemicellulose |
| pF vs aL | GH2 | 6.132537088 | 1.127845004 | 0.001482611 | GlycosideHydrolases | β-galactosidase | Others |
| pF vs aL | GH3 | 7.313375847 | 0.874785028 | 0.000577247 | GlycosideHydrolases | β-glucosidase | Cellulose |
| pF vs aL | GH13 | 4.119702346 | 0.793779169 | 0.000924271 | GlycosideHydrolases | α-amylase | Starch/oligosaccharides |
| pF vs aL | GH16 | 3.050857185 | 0.540449061 | 0.000838364 | GlycosideHydrolases | xyloglucosyltransferase | Hemicellulose |
| pF vs aL | GH18 | 5.359498351 | 0.41254337 | 0.001327543 | GlycosideHydrolases | chitinase | Chitin |
| pF vs aL | GH20 | 5.223446994 | 0.581280757 | 0.001011646 | GlycosideHydrolases | β-hexosaminidase | Chitin |
| pF vs aL | GH31 | -0.689327384 | 0.514166369 | 0.000734275 | GlycosideHydrolases | α-glucosidase | Starch/oligosaccharides |
| pF vs aL | GH36 | 5.494132214 | 0.62472215 | 0.000978085 | GlycosideHydrolases | α-galactosidase | Hemicellulose |
| pF vs aL | GH38 | 4.329638232 | 0.822080737 | 0.001351989 | GlycosideHydrolases | α-mannosidase | Hemicellulose |
| pF vs aL | GH39 | 4.538025079 | 0.65159926 | 0.000830064 | GlycosideHydrolases | α-L-iduronidase | Starch/oligosaccharides |
| pF vs aL | GH142 | 8.05498111 | 0.754618849 | 0.00053803 | GlycosideHydrolases | β-L-arabinofuranosidase | Starch/oligosaccharides |
| pF vs aL | PL17 | 5.533095572 | 0.812141096 | 0.000549452 | PolysaccharideLyases | alginate lyase | Pectin |

**Table S14.** Functional annotation, log2 fold change values and relative abundance of differentially abundant CAZy genes selected in the study (related to Figure 6B). VRS, Villum Research Station; pF, permafrost; aL, active layer; SE, standard error; Rel.ab, relative abundance.

| **VRS** | **CAZy** | **Log2FoldChange** | **SE** | **Rel.ab** | **Category** | **Function** | **Group** |
| --- | --- | --- | --- | --- | --- | --- | --- |
| pF vs aL | AA1_3 | 4.727689314 | 0.673454012 | 0.013517199 | AuxiliaryActivities | laccase | Lignin |
| pF vs aL | AA3 | 4.498195883 | 0.546746847 | 0.042091318 | AuxiliaryActivities | oxidoreductases | Lignin |
| pF vs aL | AA4 | 2.502067626 | 0.765833499 | 0.006263641 | AuxiliaryActivities | vanillyl-alcohol oxidase | Lignin |
| pF vs aL | CBM2 | 4.038955655 | 0.64030878 | 0.015650177 | Carbohydrate-BindingModules | cellulose-binding | Cellulose |
| pF vs aL | CBM4 | 6.677922565 | 0.735043696 | 0.01957548 | Carbohydrate-BindingModules | Binding of these modules has been demonstrated with xylan | Hemicellulose |
| pF vs aL | CBM5 | 5.153229757 | 0.523721823 | 0.050791791 | Carbohydrate-BindingModules | chitin-binding | Chitin |
| pF vs aL | CBM13 | 2.760139662 | 0.754293735 | 0.026088519 | Carbohydrate-BindingModules | glycoside hydrolases | Others |
| pF vs aL | CBM20 | 3.668656998 | 0.295756575 | 0.137246492 | Carbohydrate-BindingModules | starch-binding function | Starch/oligosaccharides |
| pF vs aL | CBM32 | 4.5190733 | 0.538818272 | 0.036049349 | Carbohydrate-BindingModules | Binding to polygalacturonic acid | Pectin |
| pF vs aL | CBM35 | 3.995041464 | 0.869732456 | 0.00571672 | Carbohydrate-BindingModules | xylan-degrading enzymes binds to xylan | Hemicellulose |
| pF vs aL | CBM48 | 2.743218777 | 0.663055569 | 0.00701631 | Carbohydrate-BindingModules | glycogen-binding | Starch/oligosaccharides |
| pF vs aL | CBM50 | 4.355727814 | 0.739143548 | 0.012085675 | Carbohydrate-BindingModules | chitin or peptidoglycan cleaving | Chitin |
| pF vs aL | CBM51 | 7.673449683 | 0.67960324 | 0.03824137 | Carbohydrate-BindingModules | galactose-bindind | Starch/oligosaccharides |
| pF vs aL | CBM57 | 2.892472412 | 0.700117696 | 0.005995676 | Carbohydrate-BindingModules | glycosidases | Others |
| pF vs aL | CE1 | 4.768072369 | 0.916155714 | 0.011337059 | CarbohydrateEsterases | acetyl xylan esterase | Hemicellulose |
| pF vs aL | CE11 | 2.9838212 | 0.794801439 | 0.014167881 | CarbohydrateEsterases | N-acetylglucosamine deacetylase | Chitin |
| pF vs aL | CE14 | 4.171581824 | 0.638741845 | 0.015683921 | CarbohydrateEsterases | deacetylase | Hemicellulose |
| pF vs aL | GH1 | 4.314744381 | 0.770324895 | 0.017891288 | GlycosideHydrolases | β-glucosidase | Cellulose |
| pF vs aL | GH2 | 2.525869734 | 0.557486006 | 0.015095361 | GlycosideHydrolases | β-galactosidase | Others |
| pF vs aL | GH10 | 2.880251453 | 0.736879845 | 0.006567373 | GlycosideHydrolases | endo-1,4-β-xylanase | Hemicellulose |
| pF vs aL | GH13_30 | 3.868309095 | 0.535707589 | 0.027711659 | GlycosideHydrolases | α-amylase | Starch/oligosaccharides |
| pF vs aL | GH15 | 3.567908126 | 0.555604234 | 0.017815903 | GlycosideHydrolases | glucoamylase | Starch/oligosaccharides |
| pF vs aL | GH16 | 5.779057023 | 0.952345834 | 0.007786385 | GlycosideHydrolases | xyloglucosyltransferase | Hemicellulose |
| pF vs aL | GH19 | 3.806803683 | 0.745550199 | 0.00671301 | GlycosideHydrolases | chitinase | Chitin |
| pF vs aL | GH20 | 3.063665969 | 0.437167265 | 0.019244586 | GlycosideHydrolases | β-hexosaminidase | Chitin |
| pF vs aL | GH23 | 4.678272849 | 0.641324345 | 0.014971262 | GlycosideHydrolases | peptidoglycan lyase | Cell wall/murein degradation |
| pF vs aL | GH32 | 5.99050769 | 0.900396104 | 0.011172899 | GlycosideHydrolases | invertase | Others |
| pF vs aL | GH33 | 3.563193368 | 0.68876902 | 0.011699867 | GlycosideHydrolases | sialidase | Others |
| pF vs aL | GH36 | 4.198653344 | 0.590249 | 0.016796699 | GlycosideHydrolases | α-galactosidase | Hemicellulose |
| pF vs aL | GH39 | 2.939821684 | 0.502021994 | 0.016866969 | GlycosideHydrolases | α-L-iduronidase | Starch/oligosaccharides |
| pF vs aL | GH43_22 | 4.49586031 | 0.586860443 | 0.024861092 | GlycosideHydrolases | β-xylosidase | Starch/oligosaccharides |
| pF vs aL | GH51 | 6.810734698 | 0.500442789 | 0.060300169 | GlycosideHydrolases | endoglucanase | Cellulose |
| pF vs aL | GH65 | 3.557189018 | 0.540063422 | 0.034392192 | GlycosideHydrolases | α,α-trehalase | Starch/oligosaccharides |
| pF vs aL | GH68 | 3.370723524 | 0.843765079 | 0.005570778 | GlycosideHydrolases | β-fructofuranosidase | Others |
| pF vs aL | GH77 | 3.702086163 | 0.663477655 | 0.007323149 | GlycosideHydrolases | amylomaltase | Starch/oligosaccharides |
| pF vs aL | GH94 | 2.578497441 | 0.497988992 | 0.027594714 | GlycosideHydrolases | cellobiose phosphorylase | Others |
| pF vs aL | GH99 | 3.027634626 | 0.590634293 | 0.008570917 | GlycosideHydrolases | endo-α-1,2-mannosidase | Hemicellulose |
| pF vs aL | GH113 | 3.121278921 | 0.584102084 | 0.01013362 | GlycosideHydrolases | amylo-α-1,6-glucosidase | Starch/oligosaccharides |
| pF vs aL | GH114 | 7.501992489 | 1.149362475 | 0.011323866 | GlycosideHydrolases | α-1,4-polygalactosaminidase | Others |
| pF vs aL | GH121 | 7.875894708 | 0.438300133 | 0.132586671 | GlycosideHydrolases | β-L-arabinobiosidase | Starch/oligosaccharides |
| pF vs aL | GH133 | 2.55996878 | 0.613759821 | 0.008899955 | GlycosideHydrolases | amylo-α-1,6-glucosidase | Starch/oligosaccharides |
| pF vs aL | GH144 | 2.770232884 | 0.604917919 | 0.007667604 | GlycosideHydrolases | β-1,2-glucanase | Cellulose |
| pF vs aL | PL9 | 3.184357468 | 0.968582058 | 0.006841082 | PolysaccharideLyases | pectate lyase | Pectin |
| pF vs aL | PL12 | 3.048203844 | 0.652047839 | 0.007491449 | PolysaccharideLyases | heparin-sulfate lyase | Others |
| pF vs aL | PL26 | 4.366047627 | 0.477814512 | 0.0271367 | PolysaccharideLyases | rhamnogalacturonan exolyase | Pectin |

**Table S15.** Functional annotation, log2 fold change values and relative abundance of differentially abundant CAZy genes selected in the study (related to Figure 6C). aL, active layer; LAV, Val Lavirun; VRS, Villum Research Station; SE, standard error; Rel.ab, relative abundance.

| **aL** | **Cazy** | **Log2FoldChange** | **SE** | **Rel.ab** | **Category** | **Function** | **Group** |
| --- | --- | --- | --- | --- | --- | --- | --- |
| LAV vs VRS | AA1 | 1.190046904 | 0.654285995 | 0.1358 | AuxiliaryActivities | laccase | Lignin |
| LAV vs VRS | AA3 | -2.599105507 | 0.793779531 | 0.417745 | AuxiliaryActivities | oxidoreductases | Lignin |
| LAV vs VRS | AA4 | -8.143639551 | 1.274602273 | 0.030072 | AuxiliaryActivities | vanillyl-alcohol oxidase | Lignin |
| LAV vs VRS | AA5 | 3.79953827 | 0.516070753 | 0.086875 | AuxiliaryActivities | oxidases | Lignin |
| LAV vs VRS | AA6 | -6.632779143 | 0.887137359 | 0.011701 | AuxiliaryActivities | benzoquinone reductase | Lignin |
| LAV vs VRS | AA7 | -4.772708613 | 0.974810987 | 0.090516 | AuxiliaryActivities | glucooligosaccharide oxidase | Lignin |
| LAV vs VRS | CBM4 | -3.292376014 | 0.639399524 | 0.073518 | Carbohydrate-BindingModules | Binding of these modules has been demonstrated with xylan | Hemicellulose |
| LAV vs VRS | CBM12 | -1.027001725 | 1.150273587 | 0.100923 | Carbohydrate-BindingModules | chitin-binding | Chitin |
| LAV vs VRS | CBM13 | 0.756015049 | 0.786282876 | 0.347072 | Carbohydrate-BindingModules | glycoside hydrolases | Others |
| LAV vs VRS | CBM16 | 1.054003745 | 1.033438926 | 0.073894 | Carbohydrate-BindingModules | cellulose-binding | Cellulose |
| LAV vs VRS | CBM20 | -6.691052136 | 1.109419156 | 0.073317 | Carbohydrate-BindingModules | starch-binding function | Starch/oligosaccharides |
| LAV vs VRS | CBM32 | 1.649268459 | 0.941862434 | 0.540485 | Carbohydrate-BindingModules | Binding to polygalacturonic acid | Pectin |
| LAV vs VRS | CBM47 | 3.144393519 | 0.540382774 | 0.015437 | Carbohydrate-BindingModules | Fucose-binding | Pectin |
| LAV vs VRS | CBM48 | -2.037679833 | 0.791906434 | 0.275869 | Carbohydrate-BindingModules | glycogen-binding | Starch/oligosaccharides |
| LAV vs VRS | CBM50 | -0.55036274 | 1.003959435 | 1.323379 | Carbohydrate-BindingModules | chitin or peptidoglycan cleaving | Chitin |
| LAV vs VRS | CBM51 | -5.607831124 | 0.746673949 | 0.045594 | Carbohydrate-BindingModules | galactose-binding | Starch/oligosaccharides |
| LAV vs VRS | CBM56 | -10.57911224 | 1.338250417 | 0.020001 | Carbohydrate-BindingModules | β-1,3-glucan binding | Chitin |
| LAV vs VRS | CBM57 | 1.018059759 | 1.040894924 | 0.09793 | Carbohydrate-BindingModules | glycosidases | Others |
| LAV vs VRS | CBM60 | -4.004868675 | 0.567695854 | 0.017766 | Carbohydrate-BindingModules | xylan-binding | Hemicellulose |
| LAV vs VRS | CE1 | -1.510963469 | 1.095421126 | 0.068436 | CarbohydrateEsterases | acetyl xylan esterase | Hemicellulose |
| LAV vs VRS | CE11 | 0.357844658 | 1.021434719 | 0.104854 | CarbohydrateEsterases | N-acetylglucosamine deacetylase | Chitin |
| LAV vs VRS | CE12 | 8.855475877 | 1.415103469 | 0.02927 | CarbohydrateEsterases | pectin acetylesterase | Pectin |
| LAV vs VRS | GH2 | 3.609424412 | 0.829302595 | 0.352277 | GlycosideHydrolases | β-galactosidase | Others |
| LAV vs VRS | GH3 | 1.106567266 | 0.970426769 | 0.307007 | GlycosideHydrolases | β-glucosidase | Cellulose |
| LAV vs VRS | GH4 | 5.826637704 | 0.912512154 | 0.102178 | GlycosideHydrolases | phosphate glucosidase | Starch/oligosaccharides |
| LAV vs VRS | GH8 | 3.4165147 | 0.707645411 | 0.026445 | GlycosideHydrolases | chitosanase | Chitin |
| LAV vs VRS | GH10 | -1.47571093 | 0.736351952 | 0.071114 | GlycosideHydrolases | endo-1,4-β-xylanase | Hemicellulose |
| LAV vs VRS | GH13 | 1.416025917 | 0.848168558 | 0.222156 | GlycosideHydrolases | α-amylase | Starch/oligosaccharides |
| LAV vs VRS | GH15 | -4.824333168 | 1.152269714 | 0.502895 | GlycosideHydrolases | glucoamylase | Starch/oligosaccharides |
| LAV vs VRS | GH16 | -2.907249686 | 0.974066147 | 0.195834 | GlycosideHydrolases | xyloglucosyltransferase | Hemicellulose |
| LAV vs VRS | GH18 | -1.964217065 | 0.984565076 | 0.047004 | GlycosideHydrolases | chitinase | Chitin |
| LAV vs VRS | GH20 | 4.293765422 | 0.871660307 | 0.040935 | GlycosideHydrolases | β-hexosaminidase | Chitin |
| LAV vs VRS | GH24 | -6.115618311 | 1.09033258 | 0.026604 | GlycosideHydrolases | lysozyme | Hemicellulose |
| LAV vs VRS | GH27 | 1.135736931 | 0.849361745 | 0.049664 | GlycosideHydrolases | α-galactosidase | Cellulose |
| LAV vs VRS | GH28 | -1.025527409 | 1.031243054 | 0.086805 | GlycosideHydrolases | polygalacturonase | Others |
| LAV vs VRS | GH31 | -1.468320495 | 0.989262164 | 0.030595 | GlycosideHydrolases | α-glucosidase | Starch/oligosaccharides |
| LAV vs VRS | GH32 | -9.778291186 | 1.379588242 | 0.011389 | GlycosideHydrolases | invertase | Others |
| LAV vs VRS | GH38 | 5.156175745 | 0.938512744 | 0.31639 | GlycosideHydrolases | α-mannosidase | Hemicellulose |
| LAV vs VRS | GH39 | -3.143628737 | 0.922132979 | 0.063096 | GlycosideHydrolases | α-L-iduronidase | Starch/oligosaccharides |
| LAV vs VRS | GH43 | 0.145351947 | 0.901519037 | 0.056008 | GlycosideHydrolases | β-xylosidase | Starch/oligosaccharides |
| LAV vs VRS | GH44 | 1.734926205 | 0.936042556 | 0.037137 | GlycosideHydrolases | endoglucanase | Cellulose |
| LAV vs VRS | GH55 | 2.722604005 | 0.923877695 | 0.079695 | GlycosideHydrolases | exo-β-1,3-glucanase | Cellulose |
| LAV vs VRS | GH65 | 0.583938796 | 0.851992451 | 0.236069 | GlycosideHydrolases | α,α-trehalase | Starch/oligosaccharides |
| LAV vs VRS | GH71 | 6.394507211 | 0.863956085 | 0.03409 | GlycosideHydrolases | α-1,3-glucanase | Cellulose |
| LAV vs VRS | GH77 | -6.337902939 | 0.933528542 | 0.054149 | GlycosideHydrolases | amylomaltase | Starch/oligosaccharides |
| LAV vs VRS | GH78 | 4.37976113 | 0.883527052 | 0.00925 | GlycosideHydrolases | α-L-rhamnosidase | Starch/oligosaccharides |
| LAV vs VRS | GH84 | 3.628827745 | 1.050783761 | 0.007398 | GlycosideHydrolases | N-acetyl β-glucosaminidase | Others |
| LAV vs VRS | GH87 | 4.447087418 | 0.993480148 | 0.010665 | GlycosideHydrolases | mycodextranase | Starch/oligosaccharides |
| LAV vs VRS | GH92 | -7.466180481 | 1.045417622 | 0.012434 | GlycosideHydrolases | α-1,2-mannosidase | Hemicellulose |
| LAV vs VRS | GH94 | -4.65668302 | 0.782420379 | 0.237043 | GlycosideHydrolases | cellobiose phosphorylase | Others |
| LAV vs VRS | GH125 | 7.803751829 | 1.334221177 | 0.033602 | GlycosideHydrolases | exo-α-1,6-mannosidase | Hemicellulose |
| LAV vs VRS | GH127 | -3.442726062 | 0.850801426 | 0.051754 | GlycosideHydrolases | β-L-arabinofuranosidase | Hemicellulose |
| LAV vs VRS | GH130 | 6.015974141 | 1.061454683 | 0.074048 | GlycosideHydrolases | β-1,4-mannosylglucose phosphorylase | Others |
| LAV vs VRS | GH133 | 4.700162123 | 0.883605016 | 0.134833 | GlycosideHydrolases | amylo-α-1,6-glucosidase | Starch/oligosaccharides |
| LAV vs VRS | GH135 | 5.166074984 | 0.984639368 | 0.007804 | GlycosideHydrolases | α-1,4-galactosaminogalactan hydrolase | Chitin |
| LAV vs VRS | GH144 | -7.514853607 | 1.077106777 | 0.01345 | GlycosideHydrolases | β-1,2-glucanase | Cellulose |
| LAV vs VRS | PL9 | -5.874180438 | 1.101940317 | 0.026529 | PolysaccharideLyases | pectate lyase | Pectin |
| LAV vs VRS | PL11_2 | -0.531320105 | 0.851206507 | 0.062231 | PolysaccharideLyases | rhamnogalacturonan endolyase | Pectin |

**Table S16.** Functional annotation, log2 fold change values and relative abundance of differentially abundant CAZy genes selected in the study (related to Figure 6D). pF, permafrost; LAV, Val Lavirun; VRS, Villum Research Station; SE, standard error; Rel.ab, relative abundance.

| **pF** | **Cazy** | **Log2FoldChange** | **SE** | **Rel.ab** | **Caqtegory** | **Function** | **Group** |
| --- | --- | --- | --- | --- | --- | --- | --- |
| LAV vs VRS | AA1 | -1.338992059 | 1.339405303 | 0.011932764 | AuxiliaryActivities | laccase | Lignin |
| LAV vs VRS | AA2 | -4.965187967 | 0.374706838 | 0.073799339 | AuxiliaryActivities | lignin peroxidase | Lignin |
| LAV vs VRS | AA3 | -4.23759433 | 0.832903683 | 0.839344893 | AuxiliaryActivities | oxidoreductases | Lignin |
| LAV vs VRS | AA4 | 6.842947412 | 0.581523487 | 0.039252553 | AuxiliaryActivities | vanillyl-alcohol oxidase | Lignin |
| LAV vs VRS | AA5 | -3.006858529 | 0.437606221 | 0.026675546 | AuxiliaryActivities | oxidases | Lignin |
| LAV vs VRS | AA6 | -9.790618999 | 1.563027051 | 0.010685748 | AuxiliaryActivities | benzoquinone reductase | Lignin |
| LAV vs VRS | AA7 | 2.454888757 | 0.425901764 | 0.380422726 | AuxiliaryActivities | glucooligosaccharide oxidase | Lignin |
| LAV vs VRS | CBM4 | -6.836003672 | 0.713671346 | 0.051917272 | Carbohydrate-BindingModules | Binding of these modules has been demonstrated with xylan | Hemicellulose |
| LAV vs VRS | CBM12 | -0.407369596 | 1.013699335 | 0.118117238 | Carbohydrate-BindingModules | Chitin-binding | Chitin |
| LAV vs VRS | CBM13 | -0.008352643 | 0.909197988 | 0.309216679 | Carbohydrate-BindingModules | glycoside hydrolases | Others |
| LAV vs VRS | CBM16 | -0.657378506 | 0.926783378 | 0.216678021 | Carbohydrate-BindingModules | cellulose-binding | Cellulose |
| LAV vs VRS | CBM20 | -8.961196731 | 1.21841474 | 0.197258433 | Carbohydrate-BindingModules | starch-binding function | Starch/oligosaccharides |
| LAV vs VRS | CBM32 | -0.302356948 | 0.856661427 | 0.890612531 | Carbohydrate-BindingModules | Binding to polygalacturonic acid | Pectin |
| LAV vs VRS | CBM35 | 7.034338595 | 1.12822876 | 0.009901344 | Carbohydrate-BindingModules | xylan-degrading enzymes binds to xylan | Hemicellulose |
| LAV vs VRS | CBM47 | 1.447429399 | 0.928492134 | 0.332529473 | Carbohydrate-BindingModules | Fucose-binding | Pectin |
| LAV vs VRS | CBM48 | 1.923767829 | 0.826339588 | 0.415090414 | Carbohydrate-BindingModules | glycogen-binding | Starch/oligosaccharides |
| LAV vs VRS | CBM50 | 1.402612342 | 1.024826312 | 1.373365245 | Carbohydrate-BindingModules | chitin or peptidoglycan cleaving | Chitin |
| LAV vs VRS | CBM51 | -3.580924949 | 0.891700168 | 0.092460528 | Carbohydrate-BindingModules | galactose-bindind | Starch/oligosaccharides |
| LAV vs VRS | CBM57 | 1.506231854 | 0.851740945 | 0.293455854 | Carbohydrate-BindingModules | glycosidases | Others |
| LAV vs VRS | CBM66 | 4.643456468 | 0.675194617 | 0.009597426 | Carbohydrate-BindingModules | β-fructosidase | Others |
| LAV vs VRS | CBM67 | 4.70418696 | 0.718432592 | 0.020064121 | Carbohydrate-BindingModules | L-rhamnose binding | Starch/oligosaccharides |
| LAV vs VRS | CE1 | 1.530792903 | 1.070570463 | 0.096158475 | CarbohydrateEsterases | acetyl xylan esterase | Hemicellulose |
| LAV vs VRS | CE11 | 0.226945335 | 0.97252306 | 0.266053847 | CarbohydrateEsterases | N-acetylglucosamine deacetylase | Chitin |
| LAV vs VRS | CE12 | 6.174632559 | 0.861242852 | 0.010898159 | CarbohydrateEsterases | pectin acetylesterase | Pectin |
| LAV vs VRS | CE15 | 6.564576371 | 1.154693257 | 0.007157423 | CarbohydrateEsterases | 4-O-methyl-glucuronoyl methylesterase | Lignin |
| LAV vs VRS | GH1 | 2.722528579 | 0.826516924 | 0.378154597 | GlycosideHydrolases | β-glucosidase | Cellulose |
| LAV vs VRS | GH2 | 4.994505456 | 0.937570247 | 0.41961184 | GlycosideHydrolases | β-galactosidase | Others |
| LAV vs VRS | GH4 | 4.335557798 | 0.612275925 | 0.169385173 | GlycosideHydrolases | phosphate glucosidase | Starch/oligosaccharides |
| LAV vs VRS | GH5 | 2.482886801 | 0.837394144 | 0.049284116 | GlycosideHydrolases | endo-β-1,4-glucanase | Cellulose |
| LAV vs VRS | GH8 | 7.425933184 | 0.992210895 | 0.064801407 | GlycosideHydrolases | chitosanase | Chitin |
| LAV vs VRS | GH10 | -5.983717309 | 0.718155029 | 0.01675947 | GlycosideHydrolases | endo-1,4-β-xylanase | Hemicellulose |
| LAV vs VRS | GH13 | 2.003895297 | 0.803965365 | 0.461612757 | GlycosideHydrolases | α-amylase | Starch/oligosaccharides |
| LAV vs VRS | GH15 | -1.813188792 | 0.872905306 | 0.513244213 | GlycosideHydrolases | glucoamylase | Starch/oligosaccharides |
| LAV vs VRS | GH16 | -4.315832856 | 1.123583992 | 0.197970189 | GlycosideHydrolases | xyloglucosyltransferase | Hemicellulose |
| LAV vs VRS | GH18 | 2.306918417 | 0.592799543 | 0.159542157 | GlycosideHydrolases | chitinase | Chitin |
| LAV vs VRS | GH20 | 4.756562356 | 0.472224401 | 0.377964056 | GlycosideHydrolases | β-hexosaminidase | Chitin |
| LAV vs VRS | GH25 | 3.596431331 | 0.886309736 | 0.023893976 | GlycosideHydrolases | lysozyme | Hemicellulose |
| LAV vs VRS | GH27 | 2.594954927 | 0.802481769 | 0.149403622 | GlycosideHydrolases | α-galactosidase | Cellulose |
| LAV vs VRS | GH28 | 5.239868349 | 0.567368289 | 0.04221436 | GlycosideHydrolases | polygalacturonase | Others |
| LAV vs VRS | GH30_3 | 3.776403208 | 0.524814128 | 0.014348066 | GlycosideHydrolases | endo-β-1,4-xylanase | Hemicellulose |
| LAV vs VRS | GH31 | 4.229432273 | 0.529006805 | 0.082517899 | GlycosideHydrolases | α-glucosidase | Starch/oligosaccharides |
| LAV vs VRS | GH32 | 2.459771948 | 0.629503679 | 0.047238074 | GlycosideHydrolases | invertase | Others |
| LAV vs VRS | GH33 | 4.936802643 | 0.87058567 | 0.011769005 | GlycosideHydrolases | sialidase | Others |
| LAV vs VRS | GH37 | -4.637252465 | 1.278878107 | 0.148950119 | GlycosideHydrolases | α,α-trehalase | Starch/oligosaccharides |
| LAV vs VRS | GH38 | 2.549349727 | 0.667555785 | 0.401953974 | GlycosideHydrolases | α-mannosidase | Hemicellulose |
| LAV vs VRS | GH39 | -0.05626274 | 0.942533065 | 0.221770212 | GlycosideHydrolases | α-L-iduronidase | Starch/oligosaccharides |
| LAV vs VRS | GH43 | -7.28593667 | 0.893922823 | 0.046723562 | GlycosideHydrolases | β-xylosidase | Starch/oligosaccharides |
| LAV vs VRS | GH44 | 2.222243379 | 0.646389875 | 0.103497202 | GlycosideHydrolases | endoglucanase | Cellulose |
| LAV vs VRS | GH50 | 6.492249238 | 1.162812962 | 0.006814057 | GlycosideHydrolases | β-agarase | Others |
| LAV vs VRS | GH67 | 3.391836849 | 0.492602689 | 0.014845393 | GlycosideHydrolases | α-glucuronidase | Hemicellulose |
| LAV vs VRS | GH70 | -4.57930664 | 0.389121379 | 0.054220007 | GlycosideHydrolases | dextransucrase | Starch/oligosaccharides |
| LAV vs VRS | GH71 | 3.696917704 | 0.59795365 | 0.009225291 | GlycosideHydrolases | α-1,3-glucanase | Cellulose |
| LAV vs VRS | GH76 | 6.671698431 | 0.92380994 | 0.058063652 | GlycosideHydrolases | α-1,6-mannanase | Hemicellulose |
| LAV vs VRS | GH77 | -0.365870251 | 0.840371659 | 0.314187365 | GlycosideHydrolases | amylomaltase | Starch/oligosaccharides |
| LAV vs VRS | GH84 | 5.225099584 | 0.904975908 | 0.006029077 | GlycosideHydrolases | N-acetyl β-glucosaminidase | Others |
| LAV vs VRS | GH87 | 6.062558945 | 0.768604588 | 0.01303435 | GlycosideHydrolases | mycodextranase | Starch/oligosaccharides |
| LAV vs VRS | GH89 | 5.689537157 | 0.894690757 | 0.007809754 | GlycosideHydrolases | α-N-acetylglucosaminidase | Others |
| LAV vs VRS | GH92 | -10.31714164 | 1.531691341 | 0.01535779 | GlycosideHydrolases | α-1,2-mannosidase | Hemicellulose |
| LAV vs VRS | GH94 | -4.24090825 | 0.860244 | 0.55226122 | GlycosideHydrolases | cellobiose phosphorylase | Others |
| LAV vs VRS | GH96 | -10.62437694 | 1.514531841 | 0.018896989 | GlycosideHydrolases | α-agarase | Others |
| LAV vs VRS | GH105 | -6.283790047 | 0.950226595 | 0.01023208 | GlycosideHydrolases | unsaturated rhamnogalacturonyl hydrolase | Lignin |
| LAV vs VRS | GH106 | 4.802565164 | 0.738279993 | 0.033526504 | GlycosideHydrolases | α-L-rhamnosidase | Starch/oligosaccharides |
| LAV vs VRS | GH109 | -9.842037137 | 1.549346629 | 0.010961774 | GlycosideHydrolases | α-N-acetylgalactosaminidase | Starch/oligosaccharides |
| LAV vs VRS | GH115 | -6.374475848 | 0.680485783 | 0.021661524 | GlycosideHydrolases | xylan α-1,2-glucuronidase | Hemicellulose |
| LAV vs VRS | GH125 | 5.70821701 | 0.748332896 | 0.050599591 | GlycosideHydrolases | exo-α-1,6-mannosidase | Hemicellulose |
| LAV vs VRS | GH127 | 2.708765593 | 0.952504553 | 0.048059858 | GlycosideHydrolases | β-L-arabinofuranosidase | Hemicellulose |
| LAV vs VRS | GH130 | 0.960952488 | 0.798793223 | 0.075739422 | GlycosideHydrolases | β-1,4-mannosylglucose phosphorylase | Others |
| LAV vs VRS | GH133 | 0.357819903 | 0.597490129 | 0.094642813 | GlycosideHydrolases | amylo-α-1,6-glucosidase | Starch/oligosaccharides |
| LAV vs VRS | GH135 | 4.466586954 | 0.553060938 | 0.014158592 | GlycosideHydrolases | α-1,4-galactosaminogalactan hydrolase | Chitin |
| LAV vs VRS | GH140 | 3.66325655 | 0.764556391 | 0.005631399 | GlycosideHydrolases | β-1,2-apiosidase | Starch/oligosaccharides |
| LAV vs VRS | GH141 | -5.035587303 | 1.248198917 | 0.040009629 | GlycosideHydrolases | xylanase | Hemicellulose |
| LAV vs VRS | GH144 | -4.55433972 | 0.656963444 | 0.014939465 | GlycosideHydrolases | β-1,2-glucanase | Cellulose |
| LAV vs VRS | PL9 | 4.169940476 | 0.570535383 | 0.01171654 | PolysaccharideLyases | pectate lyase | Pectin |
| LAV vs VRS | PL12 | 5.79251911 | 0.965535978 | 0.007948793 | PolysaccharideLyases | heparin-sulfate lyase | Others |

**Table S17.** Functional annotation, log2 fold change values and relative abundance of differentially abundant NCYc genes selected in the study (related to Figure 7A). LAV, Val Lavirun; pF, permafrost; aL, active layer; SE, standard error; Rel.ab, relative abundance.

| **LAV** | **NCYc** | **Log2FoldChange** | **SE** | **Rel.ab** | **Function** |
| --- | --- | --- | --- | --- | --- |
| pF vs aL | nasA | -1.383865779 | 1.179491 | 0.027831 | Assimilatory nitrate reduction |
| pF vs aL | nasB | -2.396218475 | 1.267028 | 0.002022 | Assimilatory nitrate reduction |
| pF vs aL | nirA | 2.317902427 | 0.892623 | 0.011483 | Assimilatory nitrate reduction |
| pF vs aL | NR | 3.076627728 | 1.019999 | 0.003675 | Assimilatory nitrate reduction |
| pF vs aL | napA | -1.36034001 | 1.177829 | 0.003739 | Denitrification & DNR |
| pF vs aL | napC | -6.39690495 | 1.941867 | 0.000472 | Denitrification & DNR |
| pF vs aL | narG | 5.278862368 | 1.00548 | 0.000281 | Denitrification & DNR |
| pF vs aL | narY | 4.419628545 | 1.151915 | 0.000231 | Denitrification & DNR |
| pF vs aL | narZ | 5.88904458 | 0.79706 | 0.006455 | Denitrification & DNR |
| pF vs aL | nirB | -0.167036947 | 1.055854 | 0.02776 | Denitrification & DNR |
| pF vs aL | nirD | -0.630105534 | 0.904029 | 0.003301 | Denitrification & DNR |
| pF vs aL | nirK | 1.88190248 | 1.159875 | 0.026809 | Denitrification & DNR |
| pF vs aL | nirS | 4.684608106 | 0.953655 | 0.00653 | Denitrification & DNR |
| pF vs aL | norB | -3.091614492 | 1.054944 | 0.016918 | Denitrification & DNR |
| pF vs aL | nosZ | -1.666130826 | 1.461202 | 0.013971 | Denitrification & DNR |
| pF vs aL | nrfA | -8.05792642 | 2.033484 | 0.001547 | Denitrification & DNR |
| pF vs aL | nrfC | -0.753246617 | 1.525895 | 0.002024 | Denitrification & DNR |
| pF vs aL | amoC_B | -7.726075228 | 2.059914 | 0.001224 | Nitrification |
| pF vs aL | hao | -5.03081197 | 1.440311 | 0.003468 | Nitrification |
| pF vs aL | nxrA | 1.752175326 | 0.524772 | 0.003404 | Nitrification |
| pF vs aL | nxrB | 3.708161795 | 1.144024 | 0.000185 | Nitrification |
| pF vs aL | pmoB | -5.087136628 | 1.658696 | 0.001079 | Nitrification |
| pF vs aL | ansB | 2.270851564 | 1.362196 | 0.001628 | Organic degradation and synthesis |
| pF vs aL | asnB | 0.79609969 | 1.146713 | 0.186042 | Organic degradation and synthesis |
| pF vs aL | gdh_K00261 | -1.319886283 | 1.073612 | 0.050384 | Organic degradation and synthesis |
| pF vs aL | gdh_K00262 | 1.325803239 | 1.252101 | 0.00147 | Organic degradation and synthesis |
| pF vs aL | gdh_K15371 | 0.112574476 | 0.820788 | 0.122907 | Organic degradation and synthesis |
| pF vs aL | glnA | 0.656441567 | 1.114125 | 0.117304 | Organic degradation and synthesis |
| pF vs aL | glsA | -3.615917421 | 1.155796 | 0.001344 | Organic degradation and synthesis |
| pF vs aL | gs_K00264 | 5.538111333 | 1.068543 | 0.000593 | Organic degradation and synthesis |
| pF vs aL | gs_K00265 | 1.454540691 | 1.050705 | 0.125365 | Organic degradation and synthesis |
| pF vs aL | gs_K00266 | 1.282152097 | 1.19026 | 0.044933 | Organic degradation and synthesis |
| pF vs aL | gs_K00284 | -2.642551701 | 1.229557 | 0.042304 | Organic degradation and synthesis |
| pF vs aL | nao | 6.812764013 | 1.495047 | 0.000216 | Organic degradation and synthesis |
| pF vs aL | nmo | 0.986667204 | 1.235809 | 0.118614 | Organic degradation and synthesis |
| pF vs aL | ureC | 3.996037437 | 1.07645 | 0.005351 | Organic degradation and synthesis |

**Table S18.** Functional annotation, log2 fold change values and relative abundance of differentially abundant NCYc genes selected in the study (relative to Figure 7B). VRS, Villum Research Station; pF, permafrost; aL, active layer; SE, standard error; Rel.ab, relative abundance.

| **VRS** | **NCYc** | **Log2FoldChange** | **SE** | **Rel.ab** | **Function** |
| --- | --- | --- | --- | --- | --- |
| pF vs aL | hzsC | -2.08447 | 0.595217 | 0.000588 | Anammox |
| pF vs aL | nirA | 1.365101 | 0.464004 | 0.009456 | Assimilatory nitrate reduction |
| pF vs aL | nasB | 5.993754 | 1.05409 | 0.001201 | Assimilatory nitrate reduction |
| pF vs aL | nasA | 0.393924 | 0.590604 | 0.010391 | Assimilatory nitrate reduction |
| pF vs aL | narC | 4.214499 | 0.454996 | 0.005221 | Assimilatory nitrate reduction |
| pF vs aL | narB | -2.41473 | 0.515078 | 0.001012 | Assimilatory nitrate reduction |
| pF vs aL | nosZ | -1.72083 | 0.493328 | 0.000801 | Denitrification & DNR |
| pF vs aL | norB | 0.205081 | 0.566712 | 0.002205 | Denitrification & DNR |
| pF vs aL | nirK | 2.174576 | 0.516977 | 0.006729 | Denitrification & DNR |
| pF vs aL | nirD | -2.15137 | 0.620055 | 0.000989 | Denitrification & DNR |
| pF vs aL | nirB | 1.354685 | 0.490462 | 0.013084 | Denitrification & DNR |
| pF vs aL | narZ | -2.40481 | 0.715215 | 0.000907 | Denitrification & DNR |
| pF vs aL | narJ | 2.684996 | 0.601517 | 0.001711 | Denitrification & DNR |
| pF vs aL | narI | 2.53293 | 0.729179 | 0.00143 | Denitrification & DNR |
| pF vs aL | narH | 1.213844 | 0.636082 | 0.004528 | Denitrification & DNR |
| pF vs aL | narG | 2.745075 | 0.454138 | 0.006804 | Denitrification & DNR |
| pF vs aL | napA | 0.762819 | 0.437739 | 0.005493 | Denitrification & DNR |
| pF vs aL | amoC_B | -1.81549 | 0.555125 | 0.000585 | Nitrification |
| pF vs aL | ureC | 3.01738 | 0.642971 | 0.005247 | Organic degradation and synthesis |
| pF vs aL | ureA | 3.300649 | 0.835897 | 0.000198 | Organic degradation and synthesis |
| pF vs aL | nmo | 1.989084 | 0.556944 | 0.028238 | Organic degradation and synthesis |
| pF vs aL | nao | 6.854919 | 0.562881 | 0.002171 | Organic degradation and synthesis |
| pF vs aL | gs_K00266 | 1.240687 | 0.483207 | 0.006127 | Organic degradation and synthesis |
| pF vs aL | gs_K00265 | 2.121529 | 0.490222 | 0.028138 | Organic degradation and synthesis |
| pF vs aL | gs_K00264 | 6.377923 | 0.676712 | 0.001095 | Organic degradation and synthesis |
| pF vs aL | glsA | -1.81003 | 0.535359 | 0.000838 | Organic degradation and synthesis |
| pF vs aL | glnA | 1.80393 | 0.533404 | 0.073288 | Organic degradation and synthesis |
| pF vs aL | gdh_K15371 | -1.15275 | 0.523626 | 0.007131 | Organic degradation and synthesis |
| pF vs aL | gdh_K00262 | 2.005997 | 0.617837 | 0.001572 | Organic degradation and synthesis |
| pF vs aL | gdh_K00261 | 2.297344 | 0.462054 | 0.015474 | Organic degradation and synthesis |
| pF vs aL | ansB | 1.789251 | 0.514817 | 0.016144 | Organic degradation and synthesis |

**Table S19.** Functional annotation, log2 fold change values and relative abundance of differentially abundant NCYc genes selected in the study (relative to Figure 7C). aL, active layer; LAV, Val Lavirun; VRS, Villum Research Station; SE, standard error; Rel.ab, relative abundance.

| **aL** | **NCYc** | **Log2FoldChange** | **SE** | **Rel. ab** | **Function** |
| --- | --- | --- | --- | --- | --- |
| LAV vs VRS | narB | -0.096539846 | 1.152313 | 0.198397 | Assimilatory nitrate reduction |
| LAV vs VRS | narC | -0.102079305 | 0.909129 | 0.175845 | Assimilatory nitrate reduction |
| LAV vs VRS | nasA | -1.725612003 | 1.073596 | 0.893276 | Assimilatory nitrate reduction |
| LAV vs VRS | nasB | -7.387969442 | 1.560445 | 0.019401 | Assimilatory nitrate reduction |
| LAV vs VRS | nirA | -1.636624246 | 0.935621 | 1.225586 | Assimilatory nitrate reduction |
| LAV vs VRS | NR | 1.398509209 | 0.960538 | 2.399254 | Assimilatory nitrate reduction |
| LAV vs VRS | napA | -1.340933165 | 1.110533 | 1.623052 | Denitrification & DNR |
| LAV vs VRS | napC | -6.755619324 | 1.343216 | 0.13107 | Denitrification & DNR |
| LAV vs VRS | narG | 2.282956338 | 1.543683 | 0.164448 | Denitrification & DNR |
| LAV vs VRS | narH | -7.19830573 | 1.706827 | 0.035311 | Denitrification & DNR |
| LAV vs VRS | narI | 2.498727205 | 1.131117 | 0.080587 | Denitrification & DNR |
| LAV vs VRS | narJ | 6.35337693 | 1.588119 | 0.012806 | Denitrification & DNR |
| LAV vs VRS | narY | 5.920932705 | 0.784114 | 0.328945 | Denitrification & DNR |
| LAV vs VRS | narZ | 2.765366473 | 1.022734 | 0.854643 | Denitrification & DNR |
| LAV vs VRS | nirB | 0.490426018 | 1.127332 | 0.673437 | Denitrification & DNR |
| LAV vs VRS | nirD | 4.091776092 | 1.084107 | 0.128051 | Denitrification & DNR |
| LAV vs VRS | nirK | 3.013559961 | 1.075217 | 0.961602 | Denitrification & DNR |
| LAV vs VRS | nirS | 1.969200819 | 0.526291 | 0.088887 | Denitrification & DNR |
| LAV vs VRS | norB | -0.916839122 | 1.17635 | 0.230168 | Denitrification & DNR |
| LAV vs VRS | nosZ | -0.856669077 | 1.245419 | 0.409608 | Denitrification & DNR |
| LAV vs VRS | nrfA | -5.995408272 | 1.069243 | 0.458301 | Denitrification & DNR |
| LAV vs VRS | nrfC | 0.786562358 | 1.200357 | 0.188759 | Denitrification & DNR |
| LAV vs VRS | amoA_A | 7.378169083 | 1.634553 | 0.010451 | Nitrification |
| LAV vs VRS | hao | 3.888168676 | 0.724701 | 0.038277 | Nitrification |
| LAV vs VRS | nxrA | 4.406177965 | 0.778625 | 0.038731 | Nitrification |
| LAV vs VRS | nxrB | 1.755923553 | 1.083224 | 0.074163 | Nitrification |
| LAV vs VRS | pmoA | -6.531360965 | 1.595969 | 0.036892 | Nitrification |
| LAV vs VRS | pmoB | -7.260548679 | 1.979336 | 0.018722 | Nitrification |
| LAV vs VRS | pmoC | 4.715047762 | 1.169944 | 0.01849 | Nitrification |
| LAV vs VRS | nifD | 7.775886692 | 1.535916 | 0.021927 | Nitrogen fixation |
| LAV vs VRS | ansB | 1.088916306 | 1.308303 | 0.130359 | Organic degradation and synthesis |
| LAV vs VRS | asnB | 1.869816357 | 1.083082 | 4.99557 | Organic degradation and synthesis |
| LAV vs VRS | gdh_K00260 | -7.405152037 | 1.465928 | 0.019878 | Organic degradation and synthesis |
| LAV vs VRS | gdh_K00261 | 1.41860672 | 1.015156 | 5.039687 | Organic degradation and synthesis |
| LAV vs VRS | gdh_K00262 | -3.428676219 | 0.863843 | 0.799957 | Organic degradation and synthesis |
| LAV vs VRS | gdh_K15371 | -3.524863029 | 1.028542 | 3.391035 | Organic degradation and synthesis |
| LAV vs VRS | glnA | 0.291539696 | 1.0166 | 10.15367 | Organic degradation and synthesis |
| LAV vs VRS | glsA | -3.543289648 | 1.313426 | 0.317847 | Organic degradation and synthesis |
| LAV vs VRS | gs_K00265 | -0.532734495 | 0.946821 | 7.683524 | Organic degradation and synthesis |
| LAV vs VRS | gs_K00266 | 0.737640608 | 0.993893 | 2.523246 | Organic degradation and synthesis |
| LAV vs VRS | gs_K00284 | 3.222952418 | 1.106098 | 1.421883 | Organic degradation and synthesis |
| LAV vs VRS | nmo | 0.853505797 | 1.175316 | 2.620558 | Organic degradation and synthesis |
| LAV vs VRS | ureB | 6.335000625 | 1.601226 | 0.005959 | Organic degradation and synthesis |
| LAV vs VRS | ureC | -3.20676919 | 0.89657 | 1.068231 | Organic degradation and synthesis |

**Table S20.** Functional annotation, log2 fold change values and relative abundance of differentially abundant NCYc genes selected in the study (relative to Figure 7D). pF, permafrost; LAV, Val Lavirun; VRS, Villum Research Station; SE, standard error; Rel.ab, relative abundance.

| **pF** | **NCYc** | **Log2FoldChange** | **SE** | **Rel.ab** | **Function** |
| --- | --- | --- | --- | --- | --- |
| LAV vs VRS | narB | 1.221854038 | 1.241659152 | 0.316927314 | Assimilatory nitrate reduction |
| LAV vs VRS | narC | -0.458968564 | 1.470692156 | 0.349168838 | Assimilatory nitrate reduction |
| LAV vs VRS | nasA | -1.96316096 | 1.21923155 | 1.666783857 | Assimilatory nitrate reduction |
| LAV vs VRS | nasB | -4.025281752 | 1.481790306 | 0.090550661 | Assimilatory nitrate reduction |
| LAV vs VRS | nirA | -0.115098104 | 1.003324989 | 1.268909739 | Assimilatory nitrate reduction |
| LAV vs VRS | NR | 4.207282819 | 1.04194415 | 2.242329347 | Assimilatory nitrate reduction |
| LAV vs VRS | napA | -2.556323288 | 1.187061701 | 1.280743064 | Denitrification & DNR |
| LAV vs VRS | narG | -4.231594962 | 0.956133985 | 0.637627573 | Denitrification & DNR |
| LAV vs VRS | narH | -4.378169241 | 1.355388662 | 0.176299393 | Denitrification & DNR |
| LAV vs VRS | narI | 0.363879095 | 1.474788907 | 0.125879139 | Denitrification & DNR |
| LAV vs VRS | narJ | -2.849196545 | 1.57971512 | 0.167381525 | Denitrification & DNR |
| LAV vs VRS | narY | 2.782540485 | 1.330147815 | 0.634540618 | Denitrification & DNR |
| LAV vs VRS | narZ | 0.971823927 | 0.934268985 | 2.060027542 | Denitrification & DNR |
| LAV vs VRS | nirB | -2.875528464 | 1.132525468 | 1.334593268 | Denitrification & DNR |
| LAV vs VRS | nirD | 2.070718361 | 1.318524079 | 0.069799468 | Denitrification & DNR |
| LAV vs VRS | nirK | 3.170236335 | 1.016994208 | 1.441093193 | Denitrification & DNR |
| LAV vs VRS | nirS | 2.918679268 | 0.732656138 | 0.597668663 | Denitrification & DNR |
| LAV vs VRS | norB | -5.215738644 | 1.361817167 | 0.284514293 | Denitrification & DNR |
| LAV vs VRS | nosZ | 0.654882938 | 0.884424309 | 1.094153823 | Denitrification & DNR |
| LAV vs VRS | nrfA | -7.296598899 | 1.393234955 | 0.172869444 | Denitrification & DNR |
| LAV vs VRS | nrfC | 5.244628707 | 0.734966788 | 0.717545391 | Denitrification & DNR |
| LAV vs VRS | nrfD | 4.389096065 | 1.344941158 | 0.010289848 | Denitrification & DNR |
| LAV vs VRS | amoA_A | 7.445850548 | 1.631092122 | 0.03584297 | Nitrification |
| LAV vs VRS | amoC_A | 8.866784619 | 1.60403641 | 0.028297082 | Nitrification |
| LAV vs VRS | hao | -6.016265345 | 1.707756373 | 0.011833325 | Nitrification |
| LAV vs VRS | nxrA | 5.697724903 | 0.721045668 | 0.08386226 | Nitrification |
| LAV vs VRS | nxrB | -0.905861563 | 1.248665637 | 0.048533782 | Nitrification |
| LAV vs VRS | pmoA | -9.842574044 | 1.563638771 | 0.062939569 | Nitrification |
| LAV vs VRS | pmoB | -7.156076361 | 1.471800379 | 0.047161803 | Nitrification |
| LAV vs VRS | ansB | -2.085571942 | 1.447527396 | 0.173040942 | Organic degradation and synthesis |
| LAV vs VRS | asnB | 1.277792265 | 1.124091412 | 8.534914311 | Organic degradation and synthesis |
| LAV vs VRS | gdh_K00260 | -8.32437123 | 1.636701699 | 0.020922691 | Organic degradation and synthesis |
| LAV vs VRS | gdh_K00261 | 0.5971382 | 1.009754294 | 8.886312616 | Organic degradation and synthesis |
| LAV vs VRS | gdh_K00262 | -1.556984852 | 1.107352818 | 1.542276697 | Organic degradation and synthesis |
| LAV vs VRS | gdh_K15371 | -3.469521179 | 1.159190191 | 6.917521724 | Organic degradation and synthesis |
| LAV vs VRS | glnA | -1.185787928 | 1.057707629 | 14.5961492 | Organic degradation and synthesis |
| LAV vs VRS | glsA | 1.479106726 | 0.444092375 | 0.787173362 | Organic degradation and synthesis |
| LAV vs VRS | gs_K00264 | 4.163368707 | 0.966914785 | 0.042188376 | Organic degradation and synthesis |
| LAV vs VRS | gs_K00265 | -2.546730719 | 1.05891276 | 10.8350383 | Organic degradation and synthesis |
| LAV vs VRS | gs_K00266 | 0.529304598 | 1.032946228 | 7.909120064 | Organic degradation and synthesis |
| LAV vs VRS | gs_K00284 | 3.358505073 | 1.03741289 | 0.765907676 | Organic degradation and synthesis |
| LAV vs VRS | nao | -2.410988515 | 1.424986313 | 0.103412971 | Organic degradation and synthesis |
| LAV vs VRS | nmo | 0.304667951 | 1.126466151 | 10.08576588 | Organic degradation and synthesis |
| LAV vs VRS | ureA | 1.836807185 | 1.22452161 | 0.083519265 | Organic degradation and synthesis |
| LAV vs VRS | ureC | -2.032751003 | 1.137900598 | 0.5546228 | Organic degradation and synthesis |
