# Supplementary material for: Distinct taxonomic and functional profiles of high Arctic and alpine permafrost-affected soil microbiomes
Source: Environ Microbiome. 2023 Jun 16;18:54. doi: 10.1186/s40793-023-00509-6 (PMC10276392; doi:10.1186/s40793-023-00509-6)
Supplement: Supplementary file 2 — Supplementary Figure 1. Relative abundance of the most abundant taxa at the domain level in active-layer (aL) and permafrost soils (pF). In each panel the Val Lavirun alpine site (LAV) is on the left side and the Villum Research Station High Arctic site (VRS) is on the right side. 16S/18S rRNA genes (A) were assigned to the SILVA taxonomy database v138. Predicted genes (B), eggNOG genes (C), CAZy genes (D), and NCyC (E) were assigned to the NCBI taxonomy with Kaiju v1.7.4. Relative abundance of the most abundant taxa at the phylum level (F). Supplementary Figure 2. Shannon-H diversity index based on the read abundance of different genes. Supplementary Figure 3. Functional structure of genes annotated to the different databases in alpine (Val Lavirun, LAV) and High Arctic (Villum Research Station, VRS) soil samples. Samples are visualized by principal coordinate analysis (PCoA). (A): eggNOG; (B): CAZy; (C): NCyc. aL, active layer; pF, permafrost. [file 40793_2023_509_MOESM2_ESM.docx]

**Supplementary Figure 1.** Relative abundance of the most abundant taxa at the domain level in active-layer (aL) and permafrost soils (pF). In each panel the Val Lavirun alpine site (LAV) is on the left side and the Villum Research Station High Arctic site (VRS) is on the right side. 16S/18S rRNA genes (A) were assigned to the SILVA taxonomy database v138. Predicted genes (B), eggNOG genes (C), CAZy genes (D), and NCyC (E) were assigned to the NCBI taxonomy with Kaiju v1.7.4. Relative abundance of the most abundant taxa at the phylum level (F).


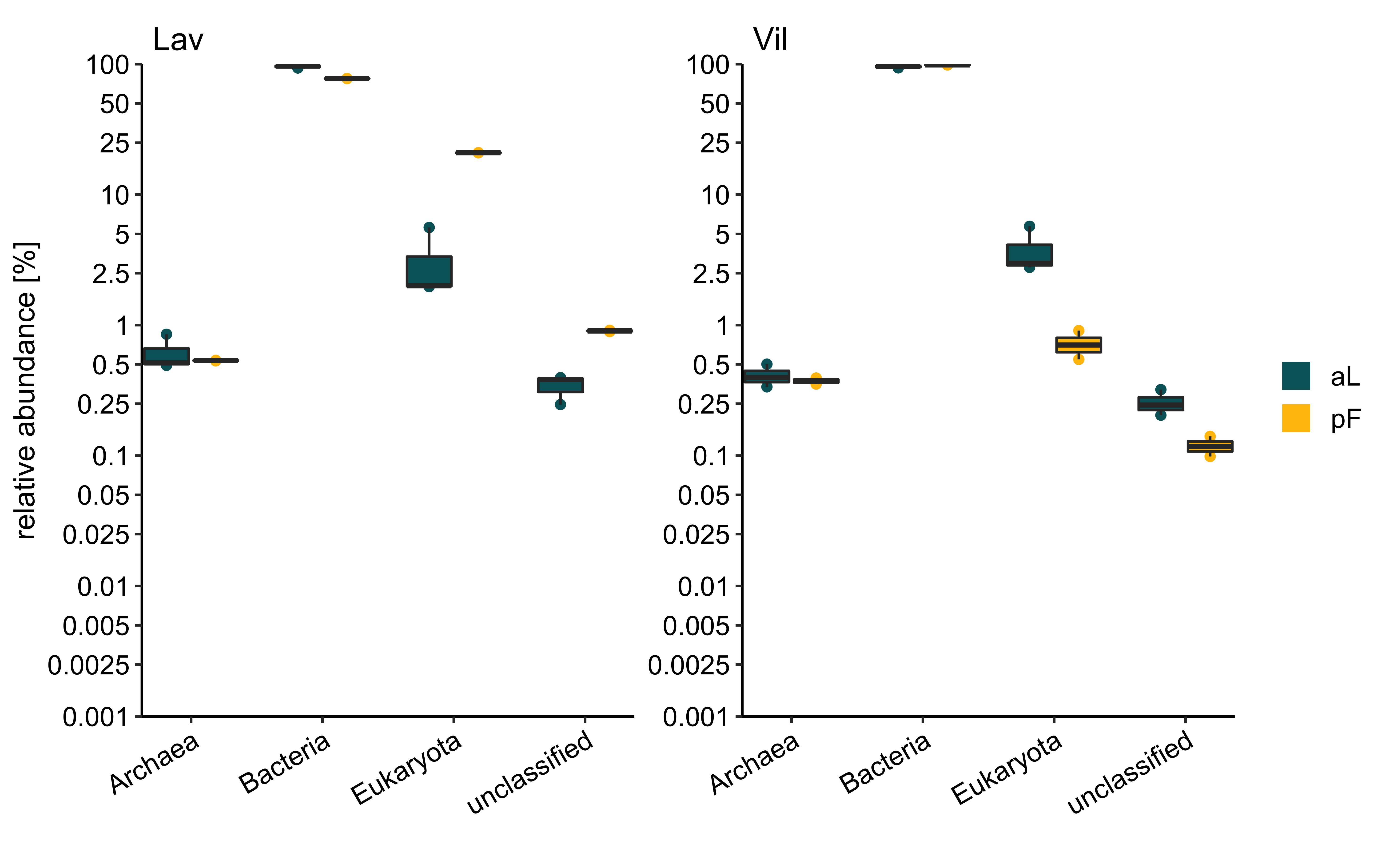

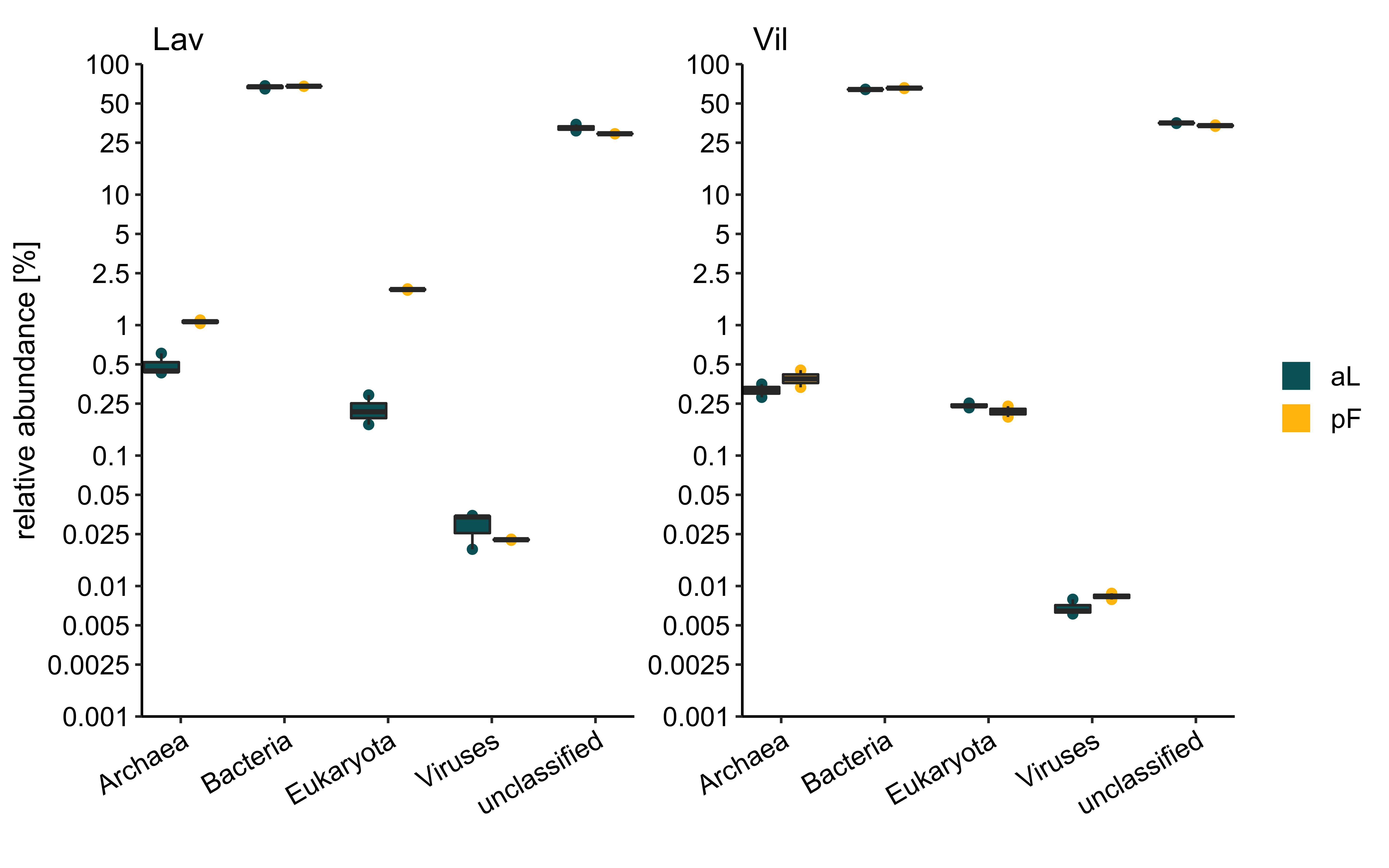

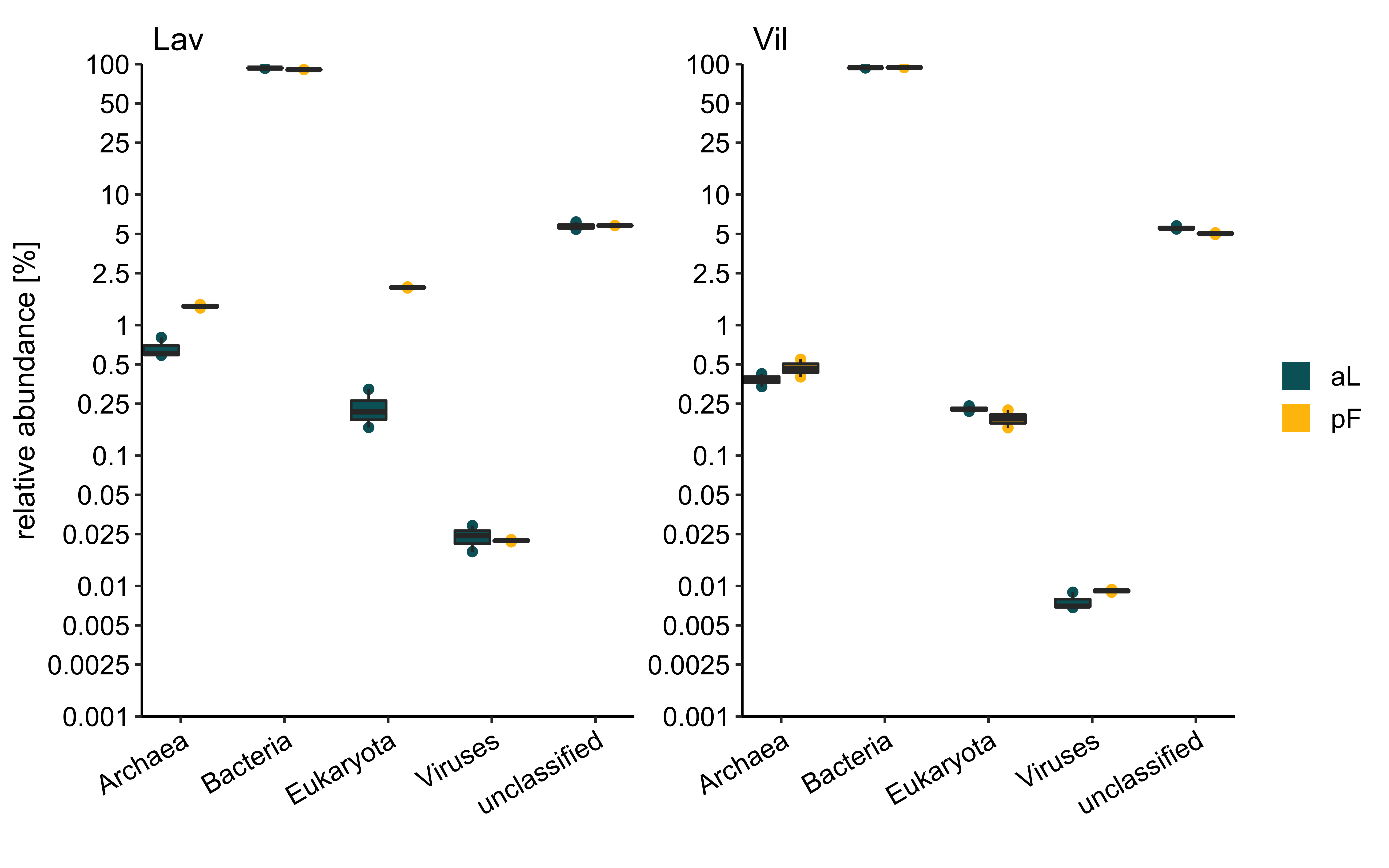

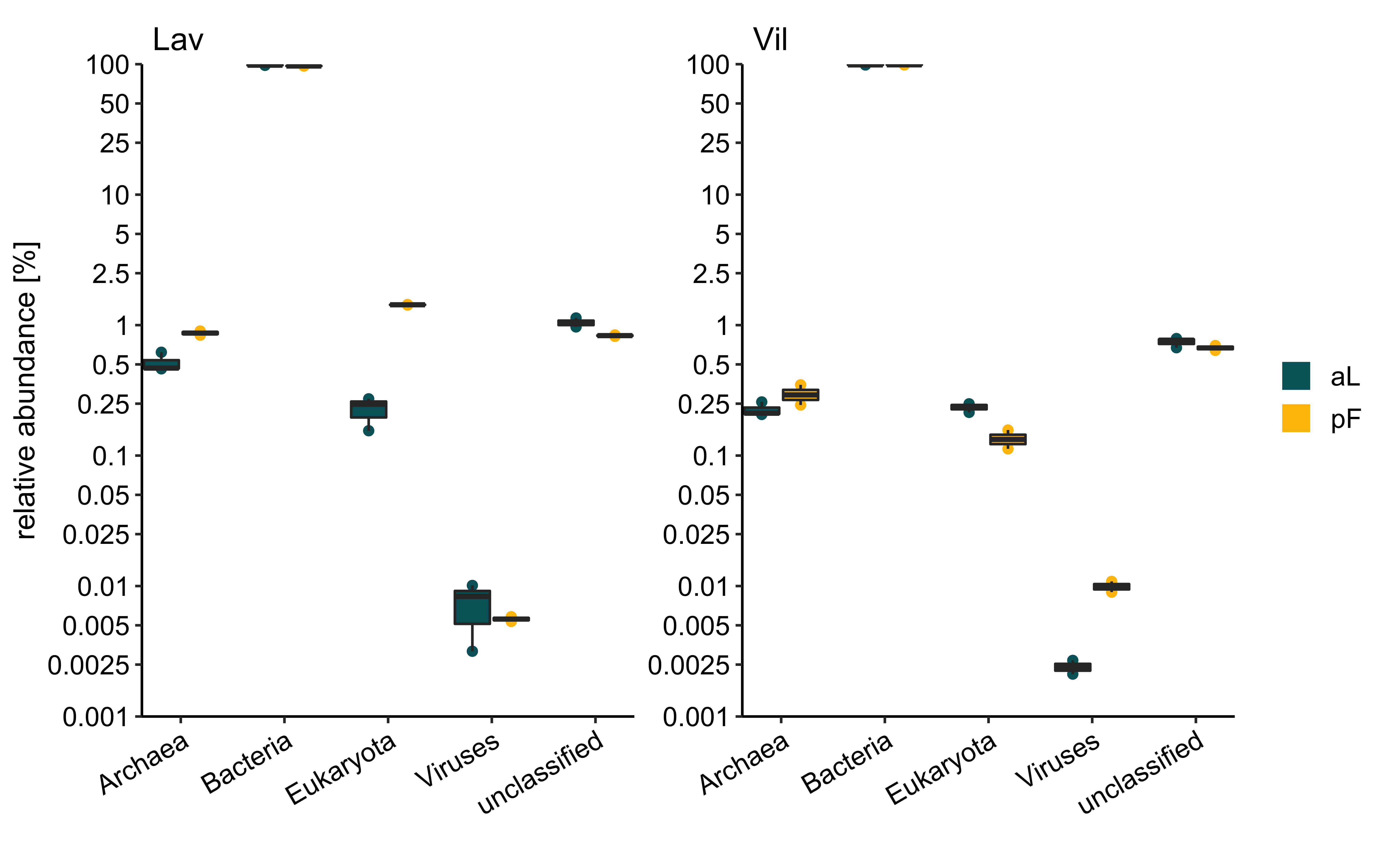

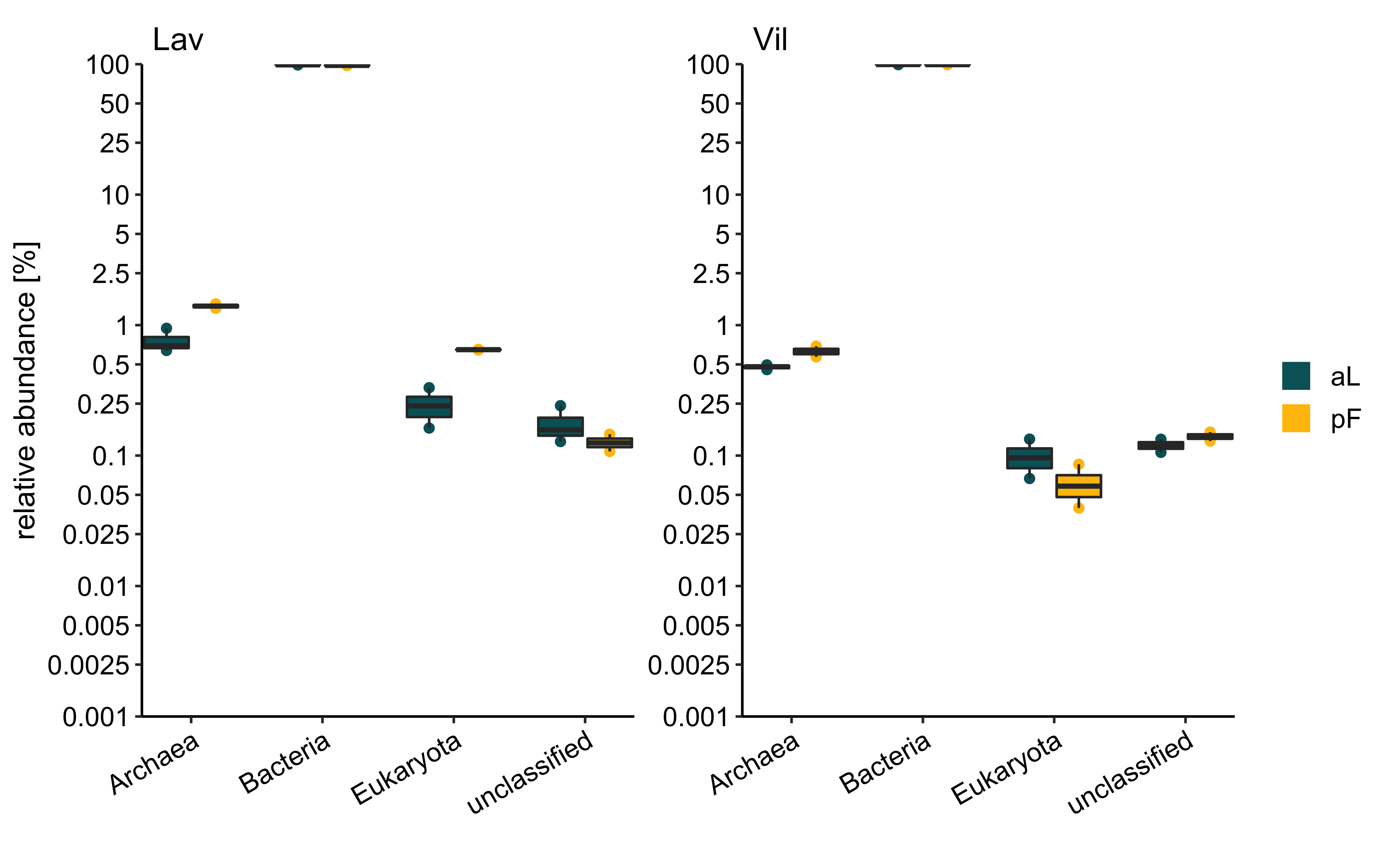

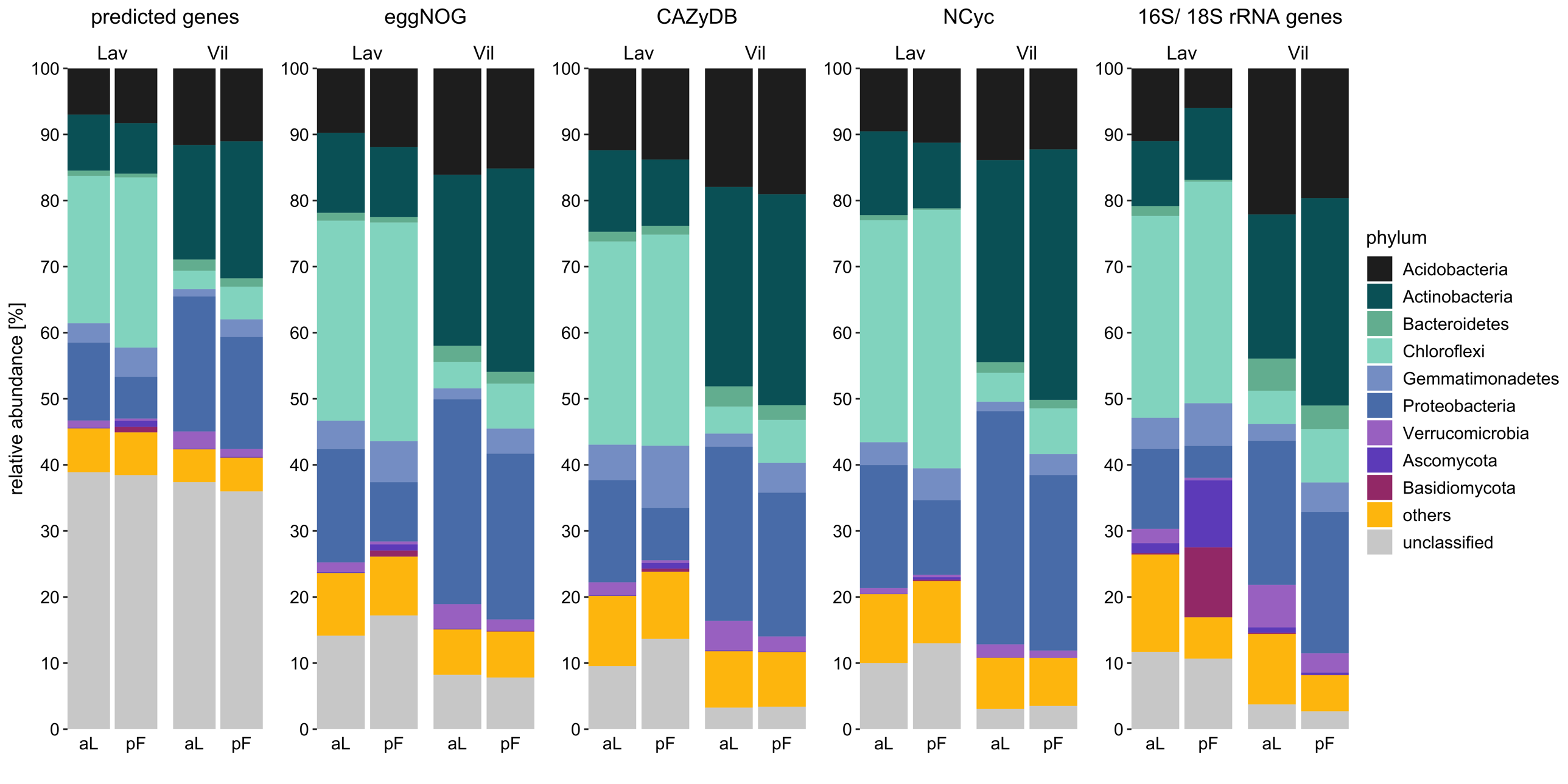


(C)

(B)

(A)

(F)

(E)

(D)

**Supplementary Figure 2.** Shannon-H diversity index based on the read abundance of different genes.

**
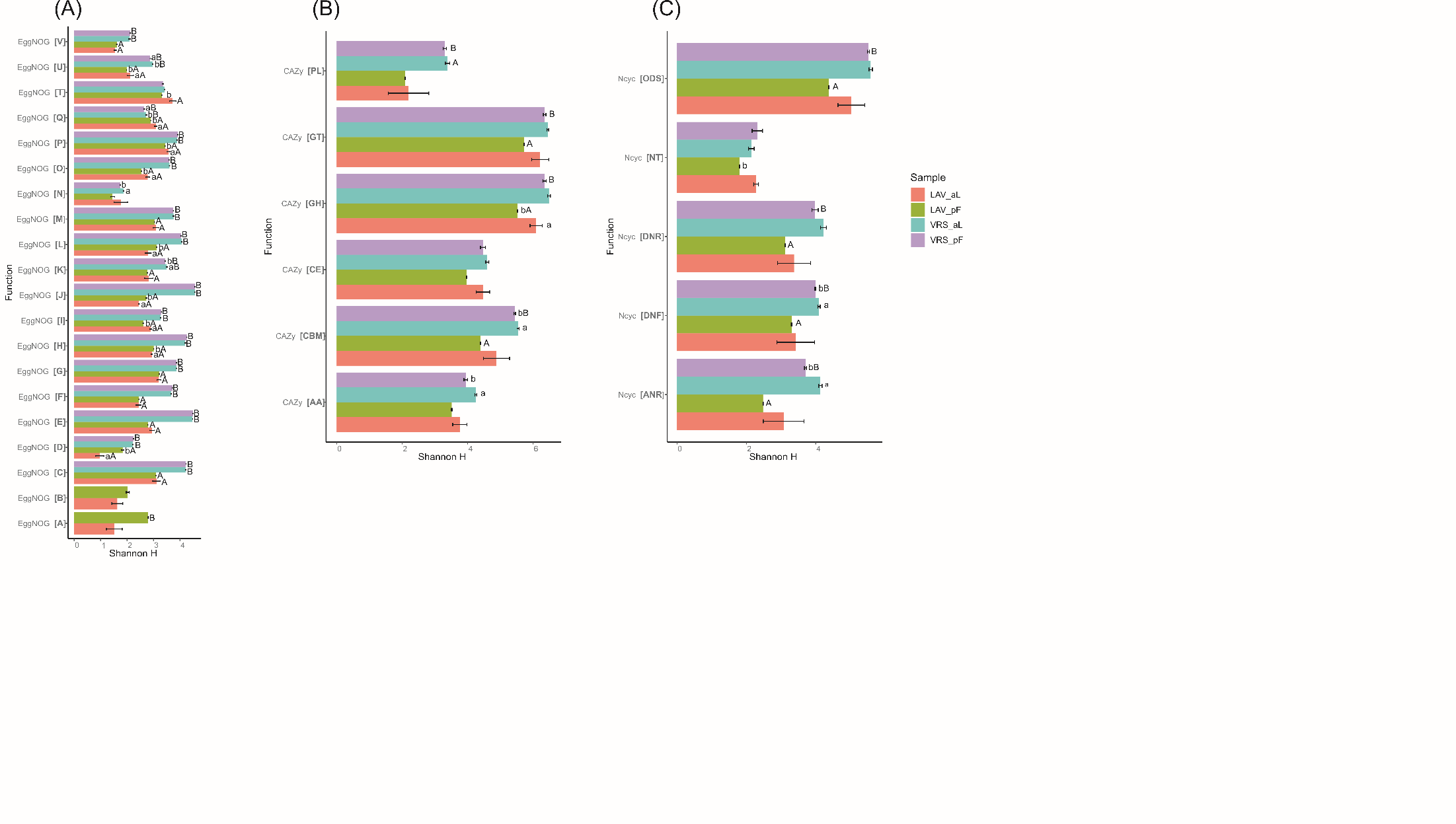
**

**(A)**: EggNOG functional categories: [A], RNA processing and modification; [B], chromatin structure and metabolism; [C], energy production and conversion; [D], cell cycle control, cell division, and chromosome partitioning; [E], amino acid transport and metabolism; [F], nucleotide transport and metabolism; [G], carbohydrate transport and metabolism; [H], coenzyme transport and metabolism; [I], lipid transport and metabolism; [J], translation, ribosomal structure, and biogenesis; [K], transcription; [L], replication, recombination, and repair; [M], cell wall/membrane/envelope biogenesis; [N], cell motility; [O], post-translational modification, protein turnover, and chaperones; [P], inorganic ionic transport and metabolism; [Q], secondary metabolite biosynthesis, transport, and catabolism; [T], signal transduction mechanisms; [U], intracellular trafficking, secretion, and vesicular transport; [V], defense mechanisms. **(B)** CAZy families: [AA], auxiliary activities; [CBM], carbohydrate-binding modules; [CE], carbohydrate esterases; [GH], glycoside hydrolases; [GT], glycosyl transferases; [PL], polysaccharide lyases. **(C)**: NCyc families: [ANR], assimilatory nitrate reduction; [DNF], denitrification; [DNR], dissimilatory nitrate reduction; [NT], nitrification; [ODS], organic degradation and synthesis. LAV, Val Lavirun (alpine site); VRS, Villum Research Station (High Arctic site); aL, active layer; pF, permafrost. Different lowercase letters indicate significant (p < 0.05) differences between aL and pF collected from the same locality. Different capital letters indicate significant (p < 0.05) differences between aL samples and between pF samples collected from LAV and VRS.

**Supplementary Figure 3.** Functional structure of genes annotated to the different databases in alpine (Val Lavirun, LAV) and High Arctic (Villum Research Station, VRS) soil samples. Samples are visualized by principal coordinate analysis (PCoA). (A): eggNOG; (B): CAZy; (C): NCyc. aL, active layer; pF, permafrost.

(A)

(B)

(C)
